# Inferring HIV-1 transmission networks and sources of epidemic spread in Africa with deep-sequence phylogenetic analysis

**DOI:** 10.1038/s41467-019-09139-4

**Published:** 2019-03-29

**Authors:** Oliver Ratmann, M. Kate Grabowski, Matthew Hall, Tanya Golubchik, Chris Wymant, Lucie Abeler-Dörner, David Bonsall, Anne Hoppe, Andrew Leigh Brown, Tulio de Oliveira, Astrid Gall, Paul Kellam, Deenan Pillay, Joseph Kagaayi, Godfrey Kigozi, Thomas C. Quinn, Maria J. Wawer, Oliver Laeyendecker, David Serwadda, Ronald H. Gray, Christophe Fraser, PANGEA Consortium and Rakai Health Sciences Program, Helen Ayles, Rory Bowden, Vincent Calvez, Myron Cohen, Ann Dennis, Max Essex, Sarah Fidler, Daniel Frampton, Richard Hayes, Joshua T. Herbeck, Pontiano Kaleebu, Cissy Kityo, Jairam Lingappa, Vladimir Novitsky, Nick Paton, Andrew Rambaut, Janet Seeley, Deogratius Ssemwanga, Frank Tanser, Gertrude Nakigozi, Robert Ssekubugu, Fred Nalugoda, Tom Lutalo, Ronald Galiwango, Fred Makumbi, Nelson K. Sewankambo, Aaron A. R. Tobian, Steven J. Reynolds, Larry W. Chang, Dorean Nabukalu, Anthony Ndyanabo, Joseph Ssekasanvu, Hadijja Nakawooya, Jessica Nakukumba, Grace N. Kigozi, Betty S. Nantume, Nampijja Resty, Jedidah Kambasu, Margaret Nalugemwa, Regina Nakabuye, Lawrence Ssebanobe, Justine Nankinga, Adrian Kayiira, Gorreth Nanfuka, Ruth Ahimbisibwe, Stephen Tomusange, Ronald M. Galiwango, Sarah Kalibbali, Margaret Nakalanzi, Joseph Ouma Otobi, Denis Ankunda, Joseph Lister Ssembatya, John Baptist Ssemanda, Robert Kairania, Emmanuel Kato, Alice Kisakye, James Batte, James Ludigo, Abisagi Nampijja, Steven Watya, Kighoma Nehemia, Margaret Anyokot, Joshua Mwinike, George Kibumba, Paschal Ssebowa, George Mondo, Francis Wasswa, Agnes Nantongo, Rebecca Kakembo, Josephine Galiwango, Geoffrey Ssemango, Andrew D. Redd, John Santelli, Caitlin E. Kennedy, Jennifer Wagman

**Affiliations:** 1 Department of Mathematics, Imperial College London, London, SW72AZ UK; 2 Department of Infectious Disease, Epidemiology School of Public Health, Imperial College London, London, W21PG UK; 3 Department of Medicine, Johns Hopkins School of Medicine, Baltimore, MD 21205-2196 USA; 4 Rakai Health Sciences Program, Entebbe, P.O.Box 49 Uganda; 5 Oxford Big Data Institute, Li Ka Shing Centre for Health Information and Discovery, Nuffield Department of Medicine, Old Road Campus, University of Oxford, Oxford, OX3 7BN UK; 6 Division of Infection and Immunity, University College London, London, WC1E 6BT UK; 7 School of Biological Sciences, University of Edinburgh, Edinburgh, EH9 3FF UK; 8 College of Health Sciences, University of KwaZulu-Natal, Durban, 4041 South Africa; 9 European Molecular Biology Laboratory-European Bioinformatics Institute (EMBL-EBI), Wellcome Genome Campus, Hinxton, Cambridgeshire CB10 1SD UK; 10 Department of Medicine, Imperial College London, London, W12 0HS UK; 11 Africa Health Research Institute, Private Bag X7, Durban, 4013 South Africa; 12 Division of Intramural Research, National Institute of Allergy and Infectious Diseases, NIH, Bethesda, MD 20892-9806 USA; 13 Department of Epidemiology, Johns Hopkins Bloomberg School of Public Health, Baltimore, MD 21205 USA; 14 Makerere University School of Public Health, Kampala, 8HQG+3V Uganda; 15 Zambart Project, Lusaka, P.O. Box 50697 Zambia; 16 Oxford Genomics Centre, The Wellcome Centre for Human Genetics, University of Oxford, Oxford, OX3 7BN UK; 17 Ecole Normale Supérieure de Lyon, Lyon, 69007 France; 18 Department of Medicine, University of North Carolina, Chapel Hill, NC 27516 USA; 19 Harvard T.H. Chan School of Public Health AIDS Initiative, Harvard T.H. Chan School of Public Health, Boston, MA 02115 USA; 20 Botswana Harvard AIDS Institute Partnership, Gaborone, Private Bag BO 320 Botswana; 21 London School of Hygiene & Tropical Medicine, London, WC1E 7HT UK; 22 Department of Global Health, University of Washington, Seattle, WA 98104 USA; 23 MRC/UVRI, Entebbe, P.O.Box 49 Uganda; 24 Joint Clinical Research Centre, Kampala, P.o.Box 10005 Uganda; 25 Department of Immunology and Infectious Diseases, Harvard T.H. Chan School of Public Health, Boston, MA 02115 USA; 26 Medical Research Council, London, WC2B 4AN UK; 27 Mailman School of Public Health, Columbia University, New York, NY 10032 USA; 28 School of Medicine, University of California San Diego, San Diego, CA 92093 USA

## Abstract

To prevent new infections with human immunodeficiency virus type 1 (HIV-1) in sub-Saharan Africa, UNAIDS recommends targeting interventions to populations that are at high risk of acquiring and passing on the virus. Yet it is often unclear who and where these ‘source’ populations are. Here we demonstrate how viral deep-sequencing can be used to reconstruct HIV-1 transmission networks and to infer the direction of transmission in these networks. We are able to deep-sequence virus from a large population-based sample of infected individuals in Rakai District, Uganda, reconstruct partial transmission networks, and infer the direction of transmission within them at an estimated error rate of 16.3% [8.8–28.3%]. With this error rate, deep-sequence phylogenetics cannot be used against individuals in legal contexts, but is sufficiently low for population-level inferences into the sources of epidemic spread. The technique presents new opportunities for characterizing source populations and for targeting of HIV-1 prevention interventions in Africa.

## Abstract (web summary)

Here, Ratmann et al. show how viral deep sequencing data can be used to reconstruct HIV-1 transmission networks and to infer the direction of transmission in these networks.

## Introduction

Large generalized epidemics of human immunodeficiency virus type 1 (HIV-1) continue to cause substantial mortality and morbidity across much of sub-Saharan Africa^1^. Rates of new infections have been reduced by adoption of prevention measures, especially antiretroviral therapy and medical male circumcision^1,2^. Despite progress, incidence levels remain well above elimination thresholds^3^. There remains an urgent need to better understand the drivers of transmission such as differential transmission by sex and age groups, especially among young women who account for 74% of new infections among adolescents in sub-Saharan Africa^4^. This may enable better targeting of prevention measures to infected people who most likely act as sources of new infection, and thus reduce transmission amongst groups most likely to sustain the epidemic. HIV-1 evolves faster than transmissions occur, so that viral sequences obtained from an individual tend to be characteristic of that individual within weeks after infection^5,6^. Therefore, viral genetic data have the potential to yield novel insights into the drivers of transmission by identifying who may have been a transmitter, and then by generalizing these findings to identify risk factors that can be directly targeted for prevention^7,8^.

Currently, phylogenetic tools to identify sources of transmission are based on Sanger sequencing, which generates a single HIV-1 consensus sequence per virus sample from an individual^9–13^. Typically one sample per individual is sequenced, and so the entire viral population from one individual is reduced into a single consensus sequence, which is insufficient to determine in which direction infections occurred^14^. For this reason source attribution methods have required data on dates of infection^15–17^ or modelling assumptions on the epidemic^9,10,12,18,19^. An advantage of source attribution methods based on additional modelling assumptions is that they may be applied with relatively small sample sizes, although it can be hard to disentangle assumptions from conclusions. For example, in ref. ^12^, it was assumed that young women are predominantly infected by older men in KwaZulu-Natal, South Africa, and it is unclear to what extent the same conclusion is based on data^20^. There is consequently a need for broadly applicable source attribution methods that are not dependent on external modelling assumptions to provide independent evidence.

**Fig. 1:**
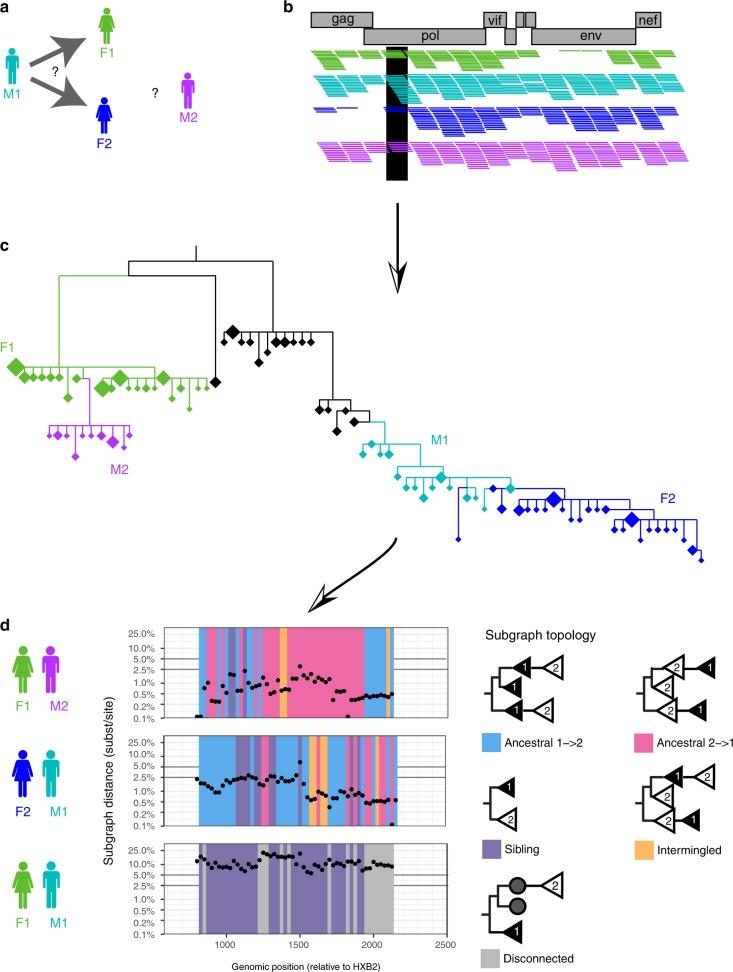
Inferring the direction of transmission from HIV-1 deep-sequence data. **a** The principles of deep-sequence viral phylogenetic analysis are illustrated on data from male M1 (turquoise) who initially reported partnership with female F1 (green), and later with female F2 (blue). We also included data from another male M2 whose virus was genetically close to that of F1, although a partnership was not reported (see Supplementary Figure 2). **b** Viral genomes from all individuals were deep-sequenced, generating short viral sequence fragments (reads) that cover the genome. Reads were mapped against HIV-1 reference sequences, and are shown as horizontal coloured lines. Genomic windows covering the whole genome were defined; one is highlighted in black. For each window, overlapping reads were extracted, aligned, and a phylogeny was reconstructed using standard methods. **c** Each phylogeny contained many unique reads per individual that tended to cluster in the phylogeny. This enabled us to reconstruct parts of the tree (subgraphs) in which virus was inferred to be in each individual (colours label individuals; diamonds indicate unique read fragments, and the size of diamonds reflects copy number). In the phylogeny shown, virus from M1 (turquoise) was phylogenetically ancestral to that from F2 (blue), suggesting that transmission occurred from M1 to F2. Similarly, virus from F1 (green) was phylogenetically ancestral to that from M2 (purple), suggesting that transmission occurred from F1 to M2. For ease of illustration, only a part of the entire reconstructed deep-sequence phylogeny is shown. HIV-1 reference sequences and virus from another phylogenetically distant individual that is in-between the F1−M2 and M1−F2 pair are shown in black. **d** Viral deep-sequence phylogenies were reconstructed for each 250 bp genomic window to determine the statistical support of inferences on transmission and the direction of transmission. For each pair of individuals, the scan plots show the shortest patristic distance between subgraphs of both individuals (*y*-axis) and the topological relationship between subgraphs of both individuals (colours) across the genome. Deep-sequence data of sufficient quality were available for the HIV-1 *gag* gene, and the genomic position on the *x*-axis indicates the start of each 250 bp read alignment

Here, we demonstrate that HIV-1 transmission networks and the direction of transmission within them can be reconstructed from deep-sequence data of a large population-based sample of infected individuals with phyloscanner^21^, a recently developed software package for viral phylogenetic inference from deep-sequence data. The accuracy in reconstructing the direction of transmission is sufficient to infer source populations, i.e. the most likely drivers of the epidemic, without assumptions on the epidemic. This finding turns into practice the theoretical prediction by Romero-Severson et al.^22^ that individuals should be represented by clusters (in short: subgraphs) of viral sequences in phylogenies when many sequence reads per individual are available, and that the phylogenetic ordering of subgraphs should allow inference of the likely direction of transmission between individuals. Figure 1 illustrates this principle. Leitner and Romero-Severson^23^ investigated which phylogenetic orderings of subgraphs (in short: subgraph topologies) can be expected among known transmission pairs. The primary aim of this study is the opposite, to establish what epidemiologic inferences can be made from observed patterns in deep-sequence phylogenies. Our population-level analysis is based on deep-sequence data that was cross-sectionally collected from 40 communities in the Rakai region of Southern Uganda. Rakai communities are predominantly small agrarian and semi-urban trading centres as well as fishing communities alongside Lake Victoria. The area was the initial epicentre of the HIV-1 epidemic in Eastern Africa, and today remains among the highest burdened districts in Uganda with an overall adult HIV prevalence that ranges from 9–26% among inland trading and agrarian communities to 38–43% among lakeside fishing communities^24,25^.

We report first that it is feasible to obtain population-based samples of HIV-1 deep-sequence data that represent a large proportion of infected individuals with unsuppressed virus in a local setting in Africa. Second, we demonstrate that deep-sequence phylogenetic analysis can be scaled from pairs in whom transmission has been suspected to population-based samples of HIV-1 epidemics. We reconstruct partial transmission networks in the absence of self-reported sexual contact information and identify pairs of individuals in whom transmission and the direction of transmission is phylogenetically inferred with high statistical support, which we call source−recipient pairs. Third, we assess the strength of deep-sequence phylogenetic inferences on direct transmission between two individuals (in short: linkage) in a large population-based sample, and the direction of transmission between two individuals via potentially unsampled intermediates. Our major finding is that the direction of transmission from a source case to a recipient could be frequently estimated with high statistical support, and that accuracy levels are sufficient for inferences into the drivers of epidemic spread at the population-level.

## Results

### Large deep-sequence data set of an African HIV-1 epidemic

**Fig. 2:**
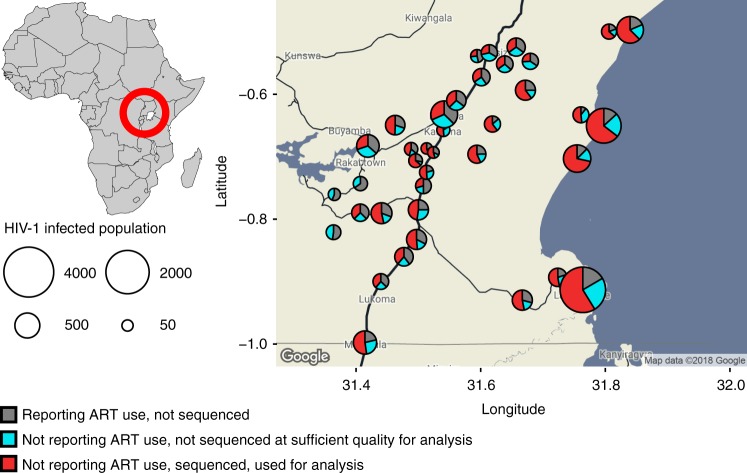
HIV-1 deep-sequencing in the Rakai Community Cohort, Uganda. Individuals aged 15–49 years were surveyed from August 2011 to January 2015 in 40 communities. In all, 5142 men and women were found positive (circles). Of those, 1264 self-reported using antiretrovirals (grey area of circles), and were not considered further as sequencing is challenging when virus is suppressed by treatment. Samples from 3878 individuals were deep-sequenced (see Methods). Of those, samples from 1226 (31.6%) individuals were not of sufficient quality for analysis (blue area of circles). Specifically, for phylogeny reconstruction, only paired-end merged reads of at least 250 base pairs (bp) in length were used, and subsequent deep-sequence inferences were performed on individuals whose reads covered the HIV-1 genome at a depth of at least 30 reads for 750 bp or more. Thus, samples from 2652 individuals (red area of circles) were used for molecular epidemiological analyses, corresponding to an estimated 45.1% of eligible and infected individuals with unsuppressed virus in RCCS communities

Between August 2011 and January 2015, 25,882 individuals aged 15–49 years were surveyed in 40 communities of the Rakai Community Cohort Study (RCCS) in Uganda (Table 1). The survey included the four largest fishing sites along Lake Victoria because of their high population-level HIV prevalence (~40%)^25^ and hypothesized role in epidemic spread. 5142 participants were HIV-positive. Reflecting previous guidelines on initiation of antiretroviral therapy (ART) during the observation period, 3878 (75.4%) infected study participants reported no ART use at time of survey. Self-reported ART use was previously validated as a proxy for actual ART use^26^, and 90% of individuals who reported using ART also had suppressed virus titres below 1000 copies per millilitre plasma blood^2^. This prompted us to focus on viral sequencing among individuals who did not report ART use. Deep-sequencing of the virus genomes was performed on 3758/3878 (96.9%) samples using the Gall et al. protocol^27^, generating thousands of short viral sequence fragments (reads) per individual. Sequencing success was comparatively modest^28^. We restricted our analysis to samples from 2652 individuals that satisfied minimum criteria on read length and depth for phylogeny reconstruction and subsequent inferences (see Methods and Supplementary Figure 1). Women and individuals of 35 years or more were under-represented in this data set when compared to infected participants, whereas individuals in fishing sites were over-represented. The overall sequence sampling fraction was high, 68.4% (2652/3878) among infected participants who did not report ART use (Fig. 2), and an estimated 65.6% (2652/4043) among infected participants with unsuppressed virus (see Methods). If we assume that individuals who were not present or did not participate at survey visits were infected with unsuppressed virus in proportion to the enrolled population, an additional 1837 individuals likely did not have suppressed viraemia, leading to an estimated sequence sampling fraction of 45.1% (2652/5880) among eligible, infected individuals with unsuppressed virus. Accounting for the previous finding that ~30% of individuals were infected by a person outside the cohort^11^, we thus expect that in approximately three of ten cases (0.451 × 0.7), our data contain the transmitter of a sequenced individual.

**Table 1:** Characteristics of the study population

	Eligible	Participated	HIV-1 positive	Reporting no ART use	Deep-sequenced	Part of phylogenetically inferred transmission chain	Highly supported phylogenetic linkage and direction of transmission
Total	37,645	25,882	5142	3878	2652	1334	554
Women	18,946	13,791	3149	2251	1447	686	279
Age							
15–24	9203 (24%)	5839 (23%)	718 (14%)	610 (16%)	403 (15%)	210 (16%)	91 (16%)
25–34	6158 (16%)	4905 (19%)	1463 (28%)	1104 (28%)	717 (27%)	356 (27%)	141 (25%)
35+	3585 (10%)	3047 (12%)	968 (19%)	537 (14%)	327 (12%)	120 (9%)	47 (8%)
Men	18,699	12,091	1993	1627	1205	648	275
Age							
15–24	7907 (21%)	4845 (19%)	237 (5%)	215 (6%)	163 (6%)	92 (7%)	33 (6%)
25–34	6317 (17%)	4052 (16%)	929 (18%)	817 (21%)	618 (23%)	351 (26%)	145 (26%)
35+	4475 (12%)	3194 (12%)	827 (16%)	595 (15%)	424 (16%)	205 (15%)	97 (18%)

ART, antiretroviral therapy

### Scaling deep-sequence phylogenetics to large data sets

**Fig. 3:**
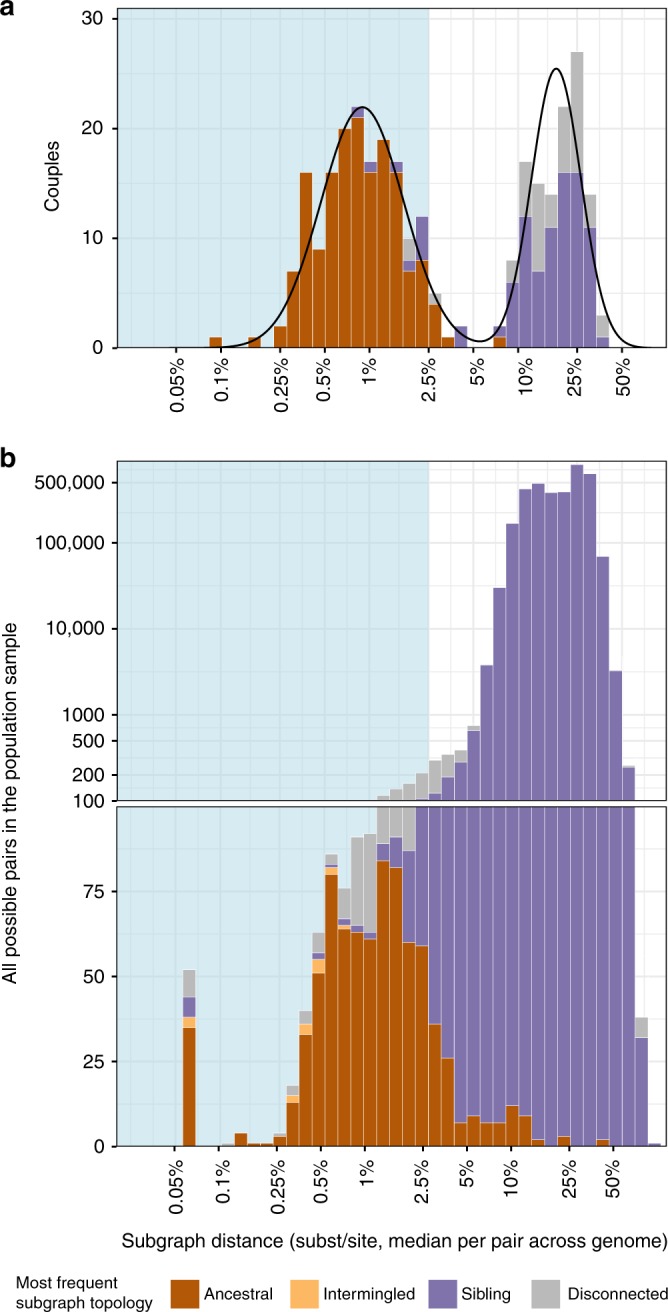
Deep-sequence phylogenetic data in the population-based sample. To highlight the characteristics of deep-sequence phylogenetic data in a population-based sample, we compared phylogenetic patterns among couples in whom both partners were positive to the patterns in the larger population-based sample. **a** Analysis of 331 couples. For each couple, their subgraph distances and subgraph topologies were calculated in each deep-sequence phylogeny across the genome as shown in Fig. 1d. Subgraph distances were standardized to the average evolutionary rate of the HIV-1 *gag* and *polymerase* genes (see Methods). Information from all deep-sequence phylogenies was summarized by median distance and the most frequent subgraph topology (colours). The distribution of median distances had a clear bimodal shape, separating couples into two groups that were either phylogenetically closely or distantly related. The distribution of median distances was well described by a two-component lognormal mixture model (black lines). 95% of couples in the first component had distances below 0.025 substitutions per site (light blue area) and 99% of couples in the first component had distances below 0.05 substitutions per site. We used these thresholds to classify couples into phylogenetically close and distant. 93.3% of phylogenetically close couples also had mostly ancestral subgraphs. **b** Analysis of 3,515,226 possible pairs in the population-based sample. For visualization purposes, smaller numbers are displayed on natural scale and larger numbers on log scale. The distribution of median distances was not bimodal, and subgraph distances did not clearly separate pairs of individuals into closely or distantly related pairs. 48/814 (5.9%) pairs with mostly ancestral subgraphs were phylogenetically distant as defined by the couples’ analysis. One hundred and eighteen phylogenetically close pairs had mostly intermingled or sibling subgraphs and were missed by subgraph ancestry, indicating that all types of subgraph topologies in combination with subgraph distance should be used for inference of population-level transmission networks

We first investigated the types of deep-sequence phylogenetic patterns that arise in known epidemiologic relationships. Our population-based sample comprised 331 concordant HIV-1-positive couples who self-identified as sexual partners. Based on previous partner analyses^16,17^, we expected that virus was transmitted in approximately 70% of couples, and that the remaining couples were separately infected by other individuals. Figure 1d illustrates a typical scan of deep-sequence phylogenies across the genome for three male−female pairs. In each phylogeny, subgraphs of reads from two individuals could either be ancestral to each other (pink if virus of the female was ancestral and blue if virus of the male was ancestral), siblings (purple), intermingled (yellow), or disconnected by one or more other individuals (grey, see Methods for full definitions and Supplementary Tables 1–3 for command line specifications of the phyloscanner software). In addition, the shortest patristic distance between subgraphs of reads from two individuals (in short: subgraph distance) reflected genetic similarity of their viruses (*y*-axis). Figure 3a summarizes these deep-sequence phylogenetic patterns across known couples. We found, first, that the distribution of subgraph distances separating partners was bimodal (Fig. 3a, showing the median distance per pair across all their phylogenies after standardizing for differences in evolutionary rates across the genome). Most couples were either phylogenetically closely related or distantly related, with intermediate distances being very rare. This suggested that transmission likely occurred among phylogenetically closely related couples, and allowed us to define distance thresholds below which transmission was likely and above which transmission could be ruled out in this population (respectively <0.025 substitutions per site and >0.05 substitutions per site, see Fig. 3a). Additional analysis of whole-genome consensus sequences further supported these findings and thresholds (Supplementary Note 2 and ref. ^29^). Second, we found that the large majority (166/178, 93.3%) of phylogenetically close couples also had ancestral subgraphs in most deep-sequence phylogenies, indicating in line with Leitner and Romero-Severson^23^ that ancestral subgraph topologies are strongly over-represented among true transmission pairs.

**Fig. 4:**
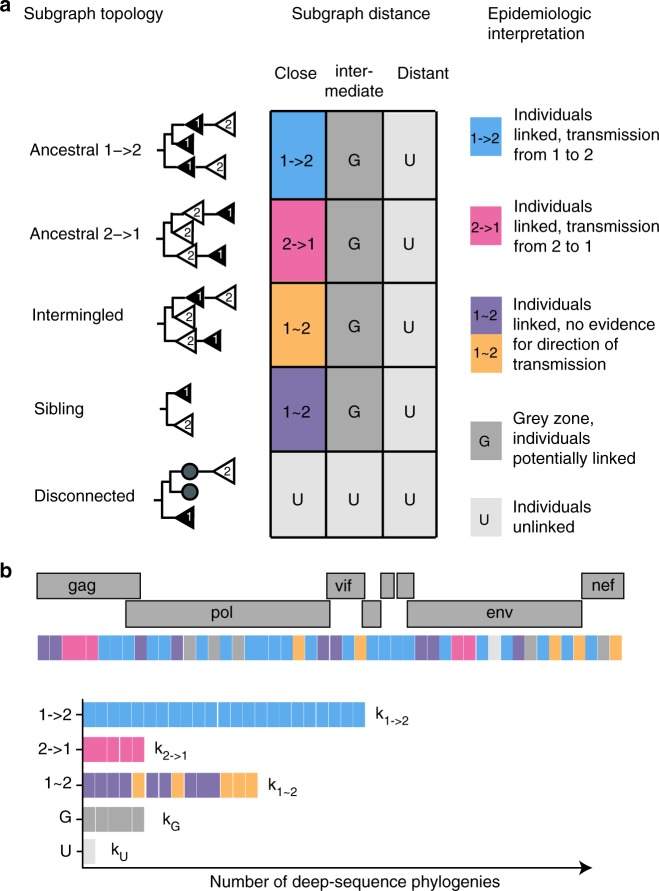
Epidemiological interpretation of deep-sequence phylogenetic data. **a** The 5 × 3 contingency table describes how deep-sequence phylogenetic patterns between two individuals were epidemiologically interpreted. Viral phylogenetic patterns between two individuals were summarized in terms of subgraph distance and subgraph topologies. There are five possible subgraph topologies between two individuals. All subgraphs of person 1 can be disconnected from the subgraphs of person 2 by another individual. If subgraphs of two individuals are adjacent, i.e. not disconnected by another individual, they can be consistently ancestral to each other in the same direction, intermingled in that some subgraphs are ancestral in one direction and others in the opposite direction, or siblings. The subgraph distance between viral subgraphs was stratified into ‘close’ (<0.025 substitutions per site), ‘intermediate’ (0.025–0.05 substitutions per site), and ‘distant’ (>0.05 substitutions per site) based on the couples’ analysis shown in Fig. 3a. Epidemiologic interpretations are indicated in colours. When only one sequence per individual is available, subgraphs of individuals correspond to the tips in a phylogeny, are either disconnected or siblings, and thus the direction of transmission is not inferable. **b** To determine the statistical support in inferences on transmission and the direction of transmission, analyses were repeated across the genome and the observed relationship types 1 → 2, 2 → 1, 1 ~ 2, G, U were counted (respectively denoted by *k*_1 → 2_, *k*_2 → 1_, *k*_1 ~ 2_, *k*_G_, *k*_U_). To avoid overconfidence, an adjustment was made to account for the fact that overlapping windows are not statistically independent (see Supplementary Note 1). Evidence for no transmission between individuals 1 and 2 was estimated by $\hat \mu _{12} = k_{\mathrm{U}}/n$; evidence for transmission between 1 and 2 was estimated by $\hat \lambda _{12} = (k_{1 \to 2} + k_{1\sim 2} + k_{2 \to 1})/n$; and evidence for transmission from 1 to 2 given that transmission occurred between 1 and 2 was estimated by ${\hat{\mathrm \delta }}_{12} = k_{1 \to 2}/(k_{1 \to 2} + k_{2 \to 1})$; see Methods for further details

Crucially, molecular epidemiologic analyses aim to infer unknown epidemiologic relationships from observed phylogenetic patterns in a population-based sample. This is a harder analytical problem compared to characterizing phylogenetic patterns among known epidemiologic relationships as in Fig. 3a, because only a tiny proportion of all pairs of individuals in a population-based sample are transmission pairs. We calculated the same phylogenetic patterns among all 3,515,226 possible pairs in our sample of 2562 individuals (see Methods), and summarized them in Fig. 3b as for the couples. With the exception of the 331 couples, sexual contacts were not known among any other of the ~3.5 m possible pairs. We found that ancestral subgraph topologies centred among pairs who were phylogenetically close: of 814 pairs with mostly ancestral subgraphs, 694 (85.3%) had phylogenetically close virus below our threshold for likely direct transmission (0.025 substitutions per site). However, 48 (5.9%) pairs had divergent virus above our threshold for ruling out direct transmission (0.05 substitutions per site). In addition, ancestry missed 118 (14.5%, 118/(694 + 118)) phylogenetically close pairs that had intermingled or sibling subgraphs in most of their deep-sequence phylogenies. Therefore, we used all types of subgraph topologies in combination with subgraph distance for inference of transmission networks from deep-sequence data. It is possible to approximate the likelihood of deep-sequence phylogenetic patterns under mathematical models of within-host viral evolution and transmission^30^. However, such models do not fully reproduce empirical observations such as preferential transmission of founder viruses^31^, and can be computationally prohibitive at large scales. For these reasons we adopted a statistical approach that is based on counting phylogenetic patterns across the genome, and calculating the proportion of deep-sequence phylogenies in support of no linkage $({\hat \mu _{ij}})$, linkage $({\hat \lambda _{ij}})$, and direction of transmission given linkage $({\hat \delta _{ij}})$; see Fig. 4 and Methods. Starting with subgraph distance, direct transmission could be ruled out for 3,513,800/3,515,226 (99.96%) pairs, leaving only 1426 potential transmission pairs. Next, we also considered information in subgraph topologies. This left 1191 potential transmission pairs that formed 446 transmission networks in the population-based sample of 2562 individuals, i.e. groups of individuals that had predominantly phylogenetically close and topologically adjacent (ancestral, intermingled or sibling) subgraphs.

**Fig. 5:**
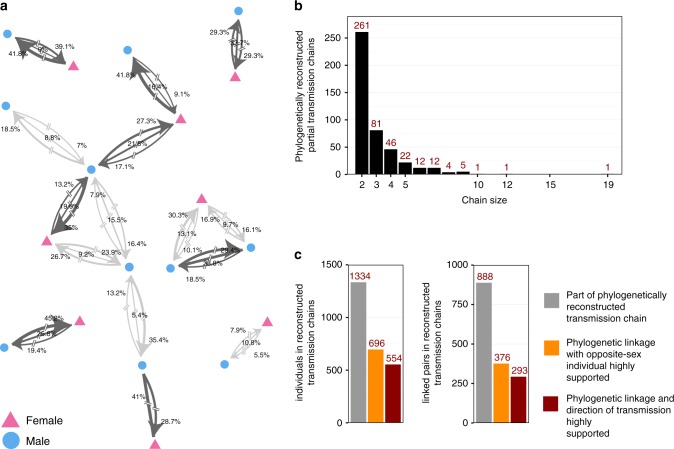
Phylogenetically reconstructed transmission networks. Four hundred and forty-six transmission networks comprising 1334 individuals and 888 linkages could be reconstructed from the population-based sample. **a** Illustrative set of six transmission networks with nodes indicating gender. In comparison to phylogenetic clustering analyses, deep-sequence phylogenetic analysis provided evidence about the direction of transmission. Edges connecting two individuals were labelled with the statistical support for transmission in the indicated direction (for directed edges), or for transmission with no evidence for direction (for undirected edges), calculated as the proportion of deep-sequence phylogenies supporting each case (see Fig. 4). The sum of the three weights quantified the phylogenetic support for direct transmission on a scale between 0 and 1 ($\hat \lambda _{ij}$, see Fig. 4). Pairs of individuals with high support for direct transmission were highlighted in dark grey ($\hat \lambda _{ij} \hskip 1.5pt > \hskip 1.5pt 0.6$). All edges were broken to indicate the possibility of unsampled intermediates. **b** Sizes of reconstructed transmission chains. The majority of transmission chains (261/446, 58.5%) were pairs, though 36 chains had more than five individuals. **c** Numbers of individuals (left) and linked pairs (right) in reconstructed transmission chains. Many linked pairs were weakly supported or between individuals of the same sex, which indicated the presence of unobserved intermediates or common sources. In all, 376 male−female pairs had high support $({\hat \lambda _{ij} \hskip 1.5pt > \hskip 1.5pt 0.6})$ (orange bars), and of those, the direction of transmission could be inferred with high support $({\hat \delta _{ij} \hskip 1.5pt > \hskip 1.5pt 0.6})$ in 293/376 (77.9%) pairs (burgundy bars)

Unlike typical phylogenetic clusters^11,12,32,33^, these transmission networks contained information on the direction of transmission (Fig. 5). Two hundred and sixty-one networks comprised just two individuals, while 36 had more than five individuals. As expected given the uncertainty in our inferences, larger networks included cycles of possible transmission flows and recipients with more than one probable source case, implying that multiple transmission chains were consistent with our phylogenetic data. We next identified the most likely transmission chains using graph theory (see Methods). This retained 888 phylogenetic linkages in 446 most likely transmission chains, of which 351 linkages had low statistical support ($\hat \lambda _{ij} \le 0.6$, see Fig. 4 and Methods for choice of threshold) and 537 linkages had high statistical support $({\hat \lambda _{ij} \hskip 1.5pt > \hskip 1.5pt 0.6})$.

### Viral deep-sequence data cannot prove HIV-1 transmission

We hypothesized that many of the 537 highly supported phylogenetic linkages were false discoveries in that transmission did not occur directly between the paired individuals. Our population-based sample did not capture all members of ongoing transmission chains, and so transmission likely occurred via unsampled intermediates in some cases. 80/537 (14.9%) of highly supported phylogenetic linkages were between two women even though HIV-1 is predominantly sexually transmitted in Africa, and extremely rarely transmitted sexually between women^34^. Considering that there were almost twice as many possible male−female combinations than female−female combinations, we calculate in Supplementary Note 3 that up to 35.4% of phylogenetically close male−female pairs of the population-based sample may not represent direct transmission events. Figure 4b illustrates this fundamental problem further: subgraph distances and topologies were not sufficient to clearly separate pairs of individuals from the population sample into two groups of closely related or distantly related pairs.

In prior work, Romero-Severson et al.^22^ proposed that direct transmission can be established with near certainty when viral sequences from two individuals are heavily intermingled in deep-sequence phylogenies. This prediction, while based on theoretical evolutionary principles and simulation, implies that deep-sequence phylogenies could be used in criminal cases of HIV-1 transmissions, and thus has important public health and human rights implications.

**Fig. 6:**
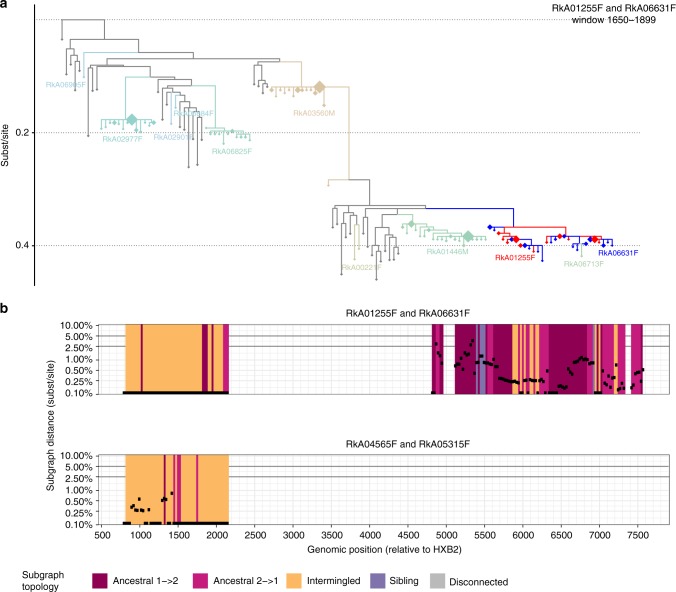
Direct transmission cannot be established when HIV-1 sequences from two individuals are intermingled in deep-sequence phylogenies. It was previously proposed that certain patterns in deep-sequence phylogenies—intermingled subgraphs of two individuals as shown in panel (**a**) in red and blue—rule out the presence of unobserved common sources and/or intermediates, and could thus prove that direct transmission occurred between two individuals. We revisited this prediction on our data, and found two female−female pairs with mostly intermingled and near identical subgraphs across the genome. These data indicate that such deep-sequence phylogenetic relationships cannot exclude the possibility of unsampled common sources or intermediates. **a** One deep-sequence phylogeny is shown for one female−female pair to illustrate their typical phylogenetic relationships. Reads from the two female−female pairs are shown in red and blue, are intermingled, and often nearly identical. The phylogenetically most closely related individuals that acted as controls are highlighted in colours, and reference sequences are shown in grey. One additional female (RkA06713F) was phylogenetically close to both females, though too poorly sampled to resolve phylogenetic relationship. The other individuals were phylogenetically distant or disconnected from the two females by HIV-1 reference sequences, with no relationship to the two females inferred. Deep-sequence phylogenies of all other windows are shown in Supplementary Data 1. **b** Phyloscan plot of subgraph distances (*y*-axis) and subgraph topologies (colour) across the genome for both female−female pairs. In the majority of deep-sequence phylogenies, both pairs had intermingled subgraphs that were also near identical

We revisited this hypothesis in our data, and found 34 phylogenetically close pairs with intermingled subgraphs across the majority of the genome. In two instances, the phylogenetically linked individuals were female (Fig. 6, corresponding deep-sequence phylogenies are reported in Supplementary Data 1), suggesting they were likely infected by a common unobserved male partner. Based on this, the phylogenetic linkages in transmission networks that we inferred from our deep-sequence data may indicate—but cannot prove—direct transmission. The difference between the theoretical expectations of Romero-Severson et al.^22^ and our observations may be explained by limited phylogenetic resolution in our reads, or may reflect greater complexity in HIV-1 evolutionary dynamics^35^.

These findings put into context that 81 (15.1%) of the 537 highly supported phylogenetic linkages were between two men. Given that the relative proportion of same-sex linkages were equivalent between men and women, our phylogenetic transmission networks provide no evidence of extensive sub-epidemics amongst men who have sex with men in rural Rakai although we cannot rule out the possibility that these may exist due to potential undersampling of widely stigmatized key populations^36^.

### The direction of transmission can be frequently inferred

We further analysed the remaining 376 highly supported male−female linkages to infer the direction of transmission (i.e. who might have infected whom, potentially via unsampled intermediates). Amongst the population-based sample, we inferred the phylogenetically likely source for 293/376 (77.9%) of linked male−female pairs (Fig. 5, $\hat \delta _{ij} \hskip 1.5pt > \hskip 1.5pt 0.6$, see Methods for choice of thresholds). In comparison, 176/376 (46.8%) of highly supported male−female linkages were between couples, and the phylogenetically likely source could be inferred in 133/176 (75.6%) couples. Inferences of these source−recipient pairs did not depend strongly on our cut-off choices (Supplementary Table 4).

### Inferring the direction of transmission has a small error

We cross-validated our findings on the direction of transmission using HIV-1 testing history and clinical data that provided independent evidence that one direction of transmission was much more likely than the other. In 36 pairs (18 couples and 18 pairs between whom sexual contact was not known), one individual tested HIV-1 negative after the other had already tested positive, and the negative individual subsequently seroconverted. The phylogenetically inferred source ($\hat \lambda _{ij} \hskip 1.5pt > \hskip 1.5pt 0.6$ and $\hat \delta _{ij} \hskip 1.5pt > \hskip 1.5pt 0.6$) was consistent with clinical evidence in 27/31 pairs, inconsistent in 4/31 pairs, and could not be inferred reliably in 5/31 pairs (Table 2; corresponding deep-sequence phylogenies are reported in Supplementary Data 2). The false discovery rate for estimating the direction of transmission amongst pairs with epidemiologically known direction of transmission was therefore 12.9% with 95% confidence interval [5.1–28.9%].

**Table 2:** Error rates in inferring the direction of HIV-1 transmission

Epidemiological evidence for direction of transmission	Phylogenetically linked pairs who reported sexual contact (couples)	Other phylogenetically linked pairs	Total
History of HIV-1 test results^a^			
Total	18	18	36
Direction consistent with clinical evidence	14	13	27
Direction ambiguous	2	3	5
Direction inconsistent with clinical evidence	2	2	4
False discovery rate	12.5% [3.5–36.0%]	13.3% [3.7–37.8%]	12.9% [5.1–28.9%]
Discrepancy in CD4 count^b^			
Total	17s	18	35
Direction consistent with clinical evidence	11	8	19
Direction ambiguous	6	5	11
Direction inconsistent with clinical evidence	0	5	5
False discovery rate	0%	38.5% [17.7–64.5%]	20.8% [9.2–40.5%]
Combined false discovery rate	7.4% [2.1–23.4%]	25% [12.7–43.4%]	16.3% [8.8–28.3%]

^a^Partner 1 tested HIV-negative, while partner 2 tested HIV-positive at or before the same time, and partner 1 was subsequently found HIV-positive

^b^Partner 1 had first CD4 measurement >800 cells per mm^3^, while partner 2 had a CD4 measurement <400 cells per mm^3^ within 2 years of the first CD4 measurement of partner 1

In 35 pairs, one individual had a CD4 cell count above 800 cells per mm^3 ^blood, indicative of being close to time of infection, while their partner was already immuno-compromised with a CD4 cell count below 400 cells per mm^3^ blood. The phylogenetically inferred source was consistent with clinical evidence in 19/35 pairs, inconsistent in 5/35 pairs, and could not be inferred reliably in 11/35 pairs. In two of the five inconsistent cases, CD4 data were only weakly indicative of the direction of transmission, and it is possible that we overestimated error rates for these pairs with CD4 data to 20.8% [9.2–40.5%] (Supplementary Note 4).

Amongst all pairs, the false discovery rate was 16.3% [8.8–28.3%]. Error rates varied slightly depending on the exact configuration of parameters in the phyloscanner analyses, though not substantially (Supplementary Tables 5–6). Similar error rates were observed in phylogenetic analysis of 454 deep-sequence data over a 320 bp region of the *env* gene among 33 couples with known direction of transmission and confirmed linked infection in the HPTN 052 trial^37^. Our findings are based on deep-sequencing of a population-based sample, and thus extend previous results to population-level inferences among individuals between whom sexual contact is not necessarily known a priori.

## Discussion

A central application of pathogen sequencing is to identify how infectious diseases continue to spread in human populations, and how new infections can be averted most effectively^38–41^. Most molecular epidemiologic studies are based on analysis of Sanger sequences, and typically identify clusters of genetically related infections in an effort to characterize ongoing transmission sources^11,32,33,42^. These approaches fail to distinguish sources from recipients of transmission within such clusters, making epidemiological inferences relevant to public health intervention challenging^7^. In contrast, deep-sequence phylogenetic analyses are based on thousands of reads per individual, and thereby provide more information into the epidemiologic relationship of individuals beyond distance measures, through the topological ordering between subgraphs of viral reads from individuals. Prior work assessed the potential of deep-sequence phylogenetic analyses on simulations and on known transmission pairs for whom at least five viral sequences were available per individual^22,23,43^. Here, we demonstrate that large population-based samples of standard deep-sequence output can be used to infer directed transmission networks of generalized HIV-1 epidemics in sub-Saharan Africa with phyloscanner^21^. Combining the patristic distance between viral subgraphs and their topological ordering in deep-sequence phylogenies, our analysis uncovered 446 partially sampled HIV-1 transmission networks in Rakai comprising 1334 individuals.

We were not able to rule out the possibility that sources were indirectly linked to recipients through unobserved individuals (i.e. intermediate partners) with deep-sequence phylogenetic analysis. One third (161/537) of phylogenetically highly supported linkages were between individuals of the same  gender, in line with incomplete sequence coverage. We also found two pairs with phylogenetic patterns previously considered strong enough to virtually exclude the possibility of common sources or recipients, but in whom both individuals were female. These findings have important implications for criminal prosecution of people living with HIV in at least 72 countries with laws penalizing HIV transmission^14,44^: even with deep-sequencing, transmission of HIV-1 cannot be proven between two individuals. Thus, communicating the limitations of deep-sequencing data is essential to prevent its misuse in criminal prosecutions. For example, we opted to visually interrupt linkages in phylogenetic transmission networks (Fig. 5), in order to highlight the possibility of unsampled cases along inferred source−recipient relationships.

We found that when many reads from different individuals are analysed together, they tend to form subgraphs with consistent ordering in deep-sequence phylogenies from across the genome. This observation enabled us to infer the source of transmission in 77.9% of 376 phylogenetically linked male−female pairs. The accuracy of our viral phylogenetic inferences regarding directionality was validated on 71 male−female pairs with clinical data that suggested transmission in one direction, with an overall false discovery rate of 16.6% [9.1–28.7%], and was thus not substantially different in a population-based sample compared to analysis of couples with known direction of transmission^37^. At this error rate, phyloscanner and similar approaches^21,37,43^ allow inferences into population-level transmission networks and the epidemiologic sources of ongoing viral spread from sequence data alone.

Our study has several weaknesses. First, sequence sampling of the infected population in RCCS communities remained incomplete. Phylogenetic inferences are expected to improve with higher sampling fraction^45^, though in practice, complete sequence sampling is hard to achieve. This study enrolled participants before immediate provision of ART was recommended in national guidelines, so that a relatively large proportion of infected individuals did not report ART use at first study visit, and could be sequenced. To perform similar phylogenetic analyses of ongoing viral spread in sub-Saharan Africa in the future, it is thus important to collect and store samples prior to ART initiation, and to investigate alternative sequencing protocols^46^. Second, relatively modest deep-sequencing quality compromised the length of deep-sequence reads^28^. Analyses were based on relatively short read alignments of 250 bp that primarily covered the *gag* gene, rather than the whole genome (Supplementary Figure 1). It is thus plausible that deep-sequence phylogenetic analyses may be more accurate than reported in this study as deep-sequence output with longer reads and greater coverage is becoming available^47^. Third, we found that inferring the direction of transmission became more challenging as the virus was increasingly closely related within individuals. We thus predict that the direction of transmission may be less frequently inferable in situations when the virus spreads more rapidly between persons, as in high-risk sexual networks among men having sex with men^9,15^, or among injecting drug users^48^. For the same reason, sources of infections may be less accurately and/or less frequently inferable for pathogens that generate within-host viral diversity at a slower pace than HIV-1 ^39,49,50^.

Whole-genome deep-sequencing is now the tool of choice in clinical practice and epidemiologic investigation for a broad range of bacterial infectious disease pathogens, and increasingly used for viral pathogens, and especially HIV-1 ^8,38,39,49,50^. Here we establish that HIV-1 phylogenetic analyses can be scaled to large population-based samples of deep-sequence data, and that the direction of transmission can be frequently inferred in reconstructed HIV-1 transmission networks. At present, more than 15,000 individuals have been deep-sequenced and linked to demographic records across sub-Saharan Africa in order to understand who is at the core and driving new infections where the burden of HIV-1 is highest, how the epidemic regenerates from older to younger generations, and how spread can be most effectively interrupted in generalized epidemics^7,8^. The phyloscanner method is applicable to these data, and we hypothesize that this innovation will help identify the key drivers of HIV-1 transmission in regions that are hardest hit by the virus, and in turn facilitate tailoring of interventions to achieve epidemic control.

## Methods

### Sample selection

Data for this study come from the Rakai Community Cohort Study (RCCS), a population-based study of HIV-1 incidence in Rakai, District Uganda. Procedures for the RCCS have been described in detail elsewhere^2^. Briefly, the RCCS conducts a census in all communities to identify eligible individuals 2 weeks before the survey. Eligible individuals include those able to give consent and between the ages of 15 and 49 years. Eligible individuals who provide written informed consent are administered a survey on their demographs, sexual behaviours and health-care seeking practices. Individuals are also asked to name their cohabitating sexual partners in order to identify couples, and to provide a serum sample for HIV-1 testing and future laboratory studies, including HIV-1 viral sequencing. Data for this particular study were collected between 2011 and 2015 from 40 agrarian, trading and fishing communities.

### Ethics

The study was independently reviewed and approved by the Ugandan Virus Research Institute, Scientific Research and Ethics Committee, Protocol GC/127/13/01/16; the Ugandan National Council of Science and Technology; and the Western Institutional Review Board, Protocol 200313317. All study participants provided written informed consent at baseline and follow-up visits using institutional review board-approved forms.

### Sampling fraction

To estimate the number of infected participants with unsuppressed virus, we first calculated the expected number of infected participants who did not use antiretrovirals at time of survey, and had thus unsuppressed virus. Participant reported ART use was previously validated as a proxy of actual ART use with a specificity of 99%^26^, giving 3878/0.99 individuals. To this, we added the expected number of participants who reported ART use but did not have suppressed virus. Ten per cent of participants reporting ART use had plasma viral loads above 1000 copies/ml plasma blood^2^, giving 1264 × 0.9 individuals, and 4043 in total. The sampling fraction was therefore estimated at 2652/4043 (65.6%) among infected participants with unsuppressed virus.

### HIV-1 deep-sequencing

Serum samples from HIV-1 seropositive persons who did not self-report ART use over the analysis period were shipped to University College London Hospital, London, United Kingdom for viral RNA extraction. RNA extraction was automated on QIAsymphony SP workstations with the QIAsymphony DSP Virus/Pathogen Kit (Cat. No. 937036, 937055; Qiagen, Hilden, Germany), followed by one-step reverse transcription polymerase chain reaction (RT-PCR)^27^. Deep-sequencing was performed on Illumina MiSeq and HiSeq instruments in the DNA pipelines core facility at the Wellcome Trust Sanger Institute, Hinxton, United Kingdom.

### Assembly of HIV-1 reads

Deep-sequencing reads were assembled with the shiver sequence assembly software^51^. Where no contigs could be generated with IVA^52^, contigs were generated with SPAdes and metaSPAdes v3.10 ^53,54^, after excluding reads classified as Homo sapiens by Kraken v0.10.5-beta^55^. Contigs with at least 300 bp matching known HIV-1 diversity were used for shiver analysis.

### Read selection

Phyloscanner version 1.1.2 ^21^ was used to merge paired-end reads, and only merged reads of at least 250 bp in length were retained for phylogeny reconstruction. Subsequent deep-sequence inferences were performed on individuals whose reads covered the HIV-1 genome at a depth of at least 30 reads for 750 bp or more. Individuals who did not have sequencing output meeting these criteria were excluded.

### Deep-sequence phylogenetic analysis

It proved computationally intractable to reconstruct viral trees from all deep-sequence reads of all individuals simultaneously. To address this challenge, samples were divided into batches of 50−75 individuals, and phyloscanner was run on all possible pairs of batches to assess deep-sequence phylogenetic relationships in all pairs of individuals in the population-based sample. The phyloscanner command line specification for this first analysis stage is given in Supplementary Tables 1 and 2. Shell scripts were used to handle calculations in parallel, and are available upon request. From stage 1 output, we identified potentially phylogenetically close pairs and, from those, networks of pairs that were connected through at least one common, phylogenetically close individual. Networks were extended to include spouses of partners in networks, couples in no network, and the ten most closely related individuals from stage 1 as controls. For computational considerations, reads of individuals that differed at one nucleotide position were merged. In a second analysis stage, phyloscanner was used to confirm potential transmission pairs by considering also the topological configuration of subgraphs in deep-sequence phylogenies, and to resolve the ordering of transmission events within transmission networks. The phyloscanner command line specification for stage 2 is given in Supplementary Table 3. In this stage, reads of individuals that differed at one nucleotide position were not merged.

### Phylogenetic relationships of virus from two individuals

The basis of viral phylogenetic analysis with phyloscanner are subgraphs, sets of tips and internal nodes of a phylogeny that are attributed to one individual with a parsimony-based algorithm^21^. A single individual can have multiple subgraphs in one tree. The following statistics were calculated to characterize the phylogenetic relationship between two individuals *i* and *j* in one phylogeny:

- Subgraph distance between *i* and *j* (Δ_*ij*_): The distance between any two subgraphs *u*, *v* is the shortest patristic distance between any nodes or tips of *u* and *v* and Δ_*ij*_ is the minimum patristic distance between subgraphs *u* from *i* and *v* from *j*. Deep-sequence phylogenies from different parts of the genome had markedly different branch lengths, reflecting evolutionary rate variation across the genome. Prior to calculating subgraph distances, we standardized phylogenies by multiplying branch lengths with the ratio of expected branch lengths in the genomic window from which the tree was reconstructed, divided by the expected branch lengths in the *gag* and *polymerase* genes (Supplementary Table 2).
- Adjacency of *i* and *j* (*A*_*ij*_): True if the shortest path between at least one subgraph *u* from *i* and *v* from *j* is not attributed to any sampled individual other than *i* and *j*, and false otherwise.
- Paths from *i* to (*P*_*ij*_): number of subgraphs from *j* which have as ancestor a subgraph from *i*.

Analyses were then based on the following phylogenetic relationship types between two individuals *i* and *j* in a viral tree:

- Phylogenetically unlinked (*U*_*ij*_*)*: *A*_*ij*_ = 0 or Δ_*ij*_ > 0.05 substitutions per site.
- Phylogenetic linkage grey zone (*G*_*ij*_): *A*_*ij*_ = 1 and Δ_*ij*_ ∈ [0.025−0.05 substitutions per site].
- Phylogenetically linked and *i* source (*i* → *j*): *A*_*ij*_ = 1 and *P*_*ij*_ ≥ 1 and *P*_*ji*_ = 0 and Δ_*ij*_ < 0.025 substitutions per site.
- Phylogenetically linked and *j* source (*j* → *i*): *A*_*ij*_ = 1 and *P*_*ji*_ ≥ 1 and *P*_*ij*_ = 0 and Δ_*ij*_ < 0.025 substitutions per site.
- Phylogenetically linked with no evidence for direction of transmission (*i* ~ *j*): *A*_*ij*_ = 1 and *P*_*ji*_ ≥ 1 and *P*_*ij*_ ≥ 1 and Δ_*ij*_ < 0.025 substitutions per site (intermingled), or *A*_*ij*_ = 1 and *P*_*ji*_ = 0 and *P*_*ij*_ = 0 and Δ_*ij*_ < 0.025 substitutions per site (sibling).

### Evidence for transmission and direction of transmission

To capture uncertainty in inferences, relationship types between reads from two individuals were evaluated on a large number of deep-sequence phylogenies that corresponded to sliding and overlapping read alignments (as shown in Fig. 1d). For each pair of individuals, the number of deep-sequence phylogenies in which *i* and *j* had one of the above five relationship types were counted (as shown in Fig. 4). The raw counts were adjusted for overlap in read alignments from which the deep-sequence phylogenies were constructed as described in Supplementary Note 1, and are denoted by *k*_U_ (unlinked), *k*_G_ (grey zone), *k*_*i*_ $_{\rightarrow}$ _*j*_ (*i* source), *k*_*j*_ $_{\rightarrow}$ _*i*_ (*j* source), *k*_*i* ~ *j*_ (no evidence for direction). After adjusting for overlap, the counts were interpreted as phylogenetic independent observations, leading to Binomial probability models for each count. Evidence for direct transmission (*λ*_*ij*_) was based on the count *k*_L_ = *k*_*i*_ $_{\rightarrow}$ _*j*_ + *k*_*j*_ $_{\rightarrow}$ _*i*_ + *k*_*i* ~ *j*_ ≥ 0, and binomial model (likelihood)

1 $$p\left( {k_{\mathrm L},n{\mathrm{|}}\lambda _{ij}} \right) = \frac{{{\mathrm{\Gamma }}(n + 1)}}{{{\mathrm{\Gamma }}(k_{\mathrm L} + 1){\mathrm{\Gamma }}(n - k_{\mathrm L} + 1)}}\lambda _{ij}^{k_{\mathrm L}}(1 - \lambda _{ij})^{n - k_{\mathrm L}},$$

where *n* = *k*_*i*_ $_{\rightarrow}$ _*j*_ + *k*_*j*_ $_{\rightarrow}$ _*i*_ + *k*_*i* ~ *j*_ + *k*_G_ + *k*_U_ > 0 and Γ is the Gamma function, with maximum likelihood estimate $\hat \lambda _{ij} = k_{\mathrm L}/n$. Evidence for ruling out direct transmission (*μ*_*ij*_) was based on *k*_U_ and total *n* as above. Evidence for the direction of transmission given linkage (*δ*_*ij*_) was based on *k*_*i*_ $_{\rightarrow}$ _*j*_ and total *k*_*i*_ $_{\rightarrow}$ _*j*_ + *k*_*j*_ $_{\rightarrow}$ _*i*_. Posterior density estimates of *λ*_*ij*_, *μ*_*ij*_ and *δ*_*ij*_ are available analytically when a Beta prior density on these parameter is chosen. We here chose a flat Beta prior density with scale and shape parameters set to 1, so that e.g. the posterior density for direct transmission is

2 $$p\left( {\lambda _{ij}{\mathrm{|}}k_{\mathrm L},n} \right) = \frac{{{\mathrm{\Gamma }}(n + 1)}}{{{\mathrm{\Gamma }}(k_{\mathrm L} + 1){\mathrm{\Gamma }}(n - k_{\mathrm L} + 1)}}\lambda _{ij}^{k_{\mathrm L}}(1 - \lambda _{ij})^{n - k_{\mathrm L}}.$$

The confidence intervals shown in Supplementary Notes 2 and 4 are 95% highest density intervals of Eq. (2). In principle, the parameters of the Beta prior could be chosen to reflect additional data such as seroconversion histories; however, care should be taken to specify informative priors based on variables such as age differences or age-specific disease prevalence^20^, in order to avoid circular inferences on who may have infected whom.

### Most likely transmission chains

Pairs of individuals between whom transmission was not excluded (when $\hat \mu _{ij} > 0.6$) defined a set of connected graphs, which we call (partially observed) transmission networks. For each network, we defined its adjacency matrix with entries $\hat \tau _{ij} = k_{i \to j} + k_{i\sim j}/2$ for *i* ≠ *j* and $\hat \tau _{ij} = 0$. Every spanning tree *c* of a network defines a possible transmission chain, and was associated with a transmission flow score over its directed edges, $\hat \tau _c = \mathop {\prod }\nolimits_{ij \in c} \hat \tau _{ij}$. The most likely transmission chain, defined by $\hat c^{{\mathrm {ML}}} = {\mathrm{argmax}}_c\,\hat \tau _c$, was calculated with Edmonds’s algorithm as implemented in the RBGL R package, version 1.55.1 ^56^.

### Classification of linked pairs and sources

Pairs in most likely transmission chains were classified as (epidemiologically) linked when $\hat \lambda _{ij} = k_{\mathrm L}/n > c$ where *n* as above and *c* = 0.6, and otherwise as potentially linked. The threshold *c* was determined as follows. Under model (1), *k*_L_ ~ Binomial (*n*, *λ*_*ij*_), where *λ*_*ij*_ indicates the strength of phylogenetic evidence for linkage. The threshold *c* was motivated by the condition that the posterior probability for *λ*_*ij*_ > 50% should be larger than *α* = 80% or alternatively *α* = 95%, i.e.

3 $$p\left( {\lambda _{ij} > 0.5{\mathrm{|}}k_{\mathrm L},n} \right) > \alpha .$$

We simplified this criterion by choosing *c* ∈ (0, 1) such that Eq. (3) holds for all *k*_L_ > *nc* for a typical whole-genome analysis. For the Rakai analysis, read alignments had a length of 250 bp, resulting in *n* = 35 non-overlapping alignments and deep-sequence phylogenies, and so with Eq. (2), we obtain *c* = 0.57 for *α* = 80% and *c* = 0.64 for *α* = 95%. The thresholds were similar for analyses based on read alignments of length 350 bp, resulting in *n* = 25 deep-sequence phylogenies, and *c* = 0.59 for *α* = 80% and *c* = 0.67 for *α* = 95%. This suggested choosing as default values *c* = 0.6 for *α* = 80% and *c* = 0.66 for *α* = 95%, with the present analysis based on *c* = 0.6 for all linkage and direction classifications.

### Reporting Summary

Further information on experimental design is available in the Nature Research Reporting Summary linked to this article.

## Supplementary information

Supplementary Information

Description of Additional Supplementary Files

Supplementary Data 1

Supplementary Data 2

Reporting Summary

## Data Availability

The deep-sequence phylogenies and basic individual-level data analysed during the current study are available in the Dryad repository, 10.5061/dryad.7h46hg2. HIV-1 reads are available on reasonable request through the PANGEA consortium (www.pangea-hiv.org) or the corresponding author. Please contact project manager Lucie Abeler-Dörner (lucie.abeler-dorner@bdi.ox.ac.uk) for further details. Additional individual-level data are available on reasonable request to RHSP or the corresponding author.

## Code availability

Code is available from https://github.com/BDI-pathogens/phyloscanner (version 1.1.2) and https://github.com/olli0601/Phyloscanner.R.utilities (version 0.7) under the GNU General Public License v3.0.
