# Supplementary material for: Inferring HIV-1 transmission networks and sources of epidemic spread in Africa with deep-sequence phylogenetic analysis
Source: Nat Commun. 2019 Mar 29;10:1411. doi: 10.1038/s41467-019-09139-4 (PMC6441045; doi:10.1038/s41467-019-09139-4)
Supplement: Supplementary file 1 — Supplementary Information [file 41467_2019_9139_MOESM1_ESM.pdf]

Inferring HIV-1 transmission networks and sources of epidemic spread in Africa with deep-sequence phylogenetic analysis

Ratmann et al.

Supplementary tables and figures

Supplementary Table 1. Specification of deep-sequence phylogenetic analysis at the population-level: inference of deep-sequence phylogenies.

Phyloscanner input parameter	Description	Value	Comments
**phyloscanner_make_trees.py**			
Input read file (no prefix)	Input read file	csv file	File specifying bam and reference files for each individual in one phyloscanner run. In total 1896 files were processed in parallel. This corresponded to batches of 50-75 individuals that systematically queried all possible pairwise phylogenetic relationship in the population sample. The aim of the stage 1 analysis (see Methods) was to identify all phylogenetically close pairs in the population sample.
--x-samtools	Samtools options	samtools	Phyloscanner default.
--x-mafft	Alignment options	mafft	Phyloscanner default.
--x-raxml	Phylogeny options	raxmlHPC-AVX -m GTRCAT --HKY85 -p 42	24 models were compared on 27 read alignments with jModelTest2, https://github.com/ddarriba/jmodeltest2[1]: 3 substitution models (all rates equal, unequal transitions/transversions, all rates unequal), 2 base frequencies (equal, unequal), 2 rate variation models (none, Γ4), 2 invariant site models (none, proportion invariant). HKY85+Γ4 had by far the largest sum of all model probabilities across all read alignments and was thus chosen for our analysis.
--alignment-of-other-refs	Background sequences	HIV1_compendium_AD_B_CPX_v2.fasta	Full-genome HIV-1 sequences in the 2012 compendium of the Los Alamos HIV sequence data base[2], that were of subtype A and D, plus HXB2 and CPX AF460972. HXB2 was used for setting default coordinates across the genome, and AF460972 was used for rooting each deep-sequence phylogeny. The alignment is included in the R package Phyloscanner.R.utilities.
--outgroupName	Root	REF_CPX_AF460972	Name of the root sequence in the background sequences file. As sensitivity analysis, a limited number of phyloscanner runs were conducted with group M root sequences. This did not have any measurable impact on tree length and node heights.
--pairwise-align-to	Sequence against which to map genome coordinates	REF_B_K03455	Name of HXB2 in the background sequences file.
--merge-paired-reads	Overlapping mates are merged into one read	Flag set	This value was set since sequencing output consisted of paired-end reads.
--discard-improper-pairs	Paired-end reads that are flagged as improperly paired are discarded	Flag not set	This function was not available at time of analysis, and is now generally recommended.
--quality-trim-ends	Phred quality score to trim ends of reads	23	This value was set to exclude poor quality ends of reads, as determined by the Phred score.
--min-internal-quality	Phred quality score to discard reads with more than one base below threshold after trimming	23	This value was set to excluded reads with poor internal quality, as determined by the Phred score.
--merging-threshold-a	Genetic similarity threshold for merging similar reads	1	Reads that differed by just one base or a one-base indel were merged in stage 1 (see Methods). This enabled us to reconstruct deep-sequence phylogenies from reads of approximately 75 individuals per run, and keeping a computational budget of at most 24 hours per deep-sequence phylogeny reconstruction.
--min-read-count	Minimum count of unique reads so they were included in read alignments	2	Unique reads that occurred, after merging, just once were ignored in stage 1 (see Method). This enabled us to reconstruct deep-sequence phylogenies from reads of approximately 75 individuals per run, and keeping a

			computational budget of at most 24 hours per deep-sequence phylogeny reconstruction.
--check-recombination	Perform triplet recombination check	Flag not set	Computationally too expensive for the read alignments as specified above. No recombination checks were performed.
--dont-check-duplicates	Compare reads between individuals to find duplicates	Flag set	The resulting list of potential duplicates was used to discard potential contaminants at a later stage.
--windows	Start and end coordinates of genomic windows	From 800 to 9400 in 125bp increments of 250bp windows	The window length was chosen so that 75% of subjects were retained in analysis. Windows were incremented by 125bp, which we considered sufficient to identify individuals with phylogenetically close subgraphs.
--num-bootstraps	Number of bootstrap trees reconstructed per read alignment	None	Rather than bootstrapping non-overlapping read alignments, we opted instead to reconstruct deep-sequence phylogenies from tightly overlapping read alignments. This procedure aimed at capturing in addition to phylogenetic uncertainty also uncertainty in deep sequencing and alignment reconstruction.

Supplementary Table 2. Specification of deep-sequence phylogenetic analysis at the population-level: inference of phylogenetically close individuals.

**Phyloscanner input parameter**	**Description**	**Value**	**Comments**
**NormalisationLookupWriter.R**			
--norm.file.name	Reference table of tree summary statistics across the genome	hiv.hxb2.norm.constants.rda	To capture changes in evolutionary rates across the HIV-1 genome, Group M sequences in the 2012 compendium alignment of the Los Alamos HIV sequence data base[2] were selected, trimmed to 300bp regions that shifted across the genome by 1bp, phylogenies were reconstructed with RAxML[3] using default options, and several tree summaries were calculated (median pairwise distance, mean pairwise distance, maximum pairwise distance, sum of branch lengths). This file is part of the R package Phyloscanner.R.utilities. Branch lengths of each deep-sequence phylogeny were multiplied with a normalization factor derived from one of these statistics. Specifically, we calculated the average statistic in a reference gene, and then calculated the ratio of that statistic at any base pair divided by the average in the reference gene.
--norm.var	Tree summary statistic used.	median pairwise distance	Phyloscanner default.
--standardize	Normalise summary statistic so that its average on the concatenated *gag+pol* gene equals one.	Flag set	Sets the reference gene to the *gag+pol* gene region. This implied that the evolutionary distances shown in scanplots and reported in the main text can be interpreted as average distances expected in the *pol* gene.
**parsimony_based_blacklister.R**			
--multifurcation Threshold	Threshold to collapse branches in NGS phylogenies into polytomies.	1e-5	RAxML returns strictly bifurcating trees with minimum-length branches that in fact imply multifurcations. The minimum length can vary, and we set the threshold to the typical minimum branch length value given by RAxML[3].
--sankoffK	K parameter in Sankoff cost matrix	20	This value was chosen by testing different values of *k* on the whole dataset and examining the distribution of multiple infections that they give. From this analysis, we recommend setting the value to the reciprocal of a pairwise genetic diversity (in substitutions per site) that would be unrealistic to see in an infection with a single source. Based on the analysis reported in Figure 3A, that value would be 0.05 substitutions per site.
--rawThreshold	Subgraphs with fewer read counts are flagged	10	Divergent within-host subgraphs containing just one read could be contaminants, and should be excluded from further analysis. We opted for a threshold of 10 after analyzing the

	as potential contaminants and discarded.		frequency of divergent subgraphs with few reads, see supplementary text S2.
--ratioThreshold	Subgraphs, whose tip count divided by that of another subgraph from the same subject is less than this threshold, are flagged as potential contaminants and discarded.	0	Additional and/or alternative threshold for excluding potential contaminants. We only used a threshold on the absolute number of reads in divergent subgraphs.
**downsample_reads.R**			
--maxReads PerHost	Downsample reads to at most this number if more reads are present	50	Reads were downsampled to reduce preferential assignment of well-sampled individuals as being ancestral to others. There is currently no strong evidence suggesting that this option is necessary for deep-sequence phylogenetic analysis.
--excludeUnder represented	Hosts with less than maxReadsPerHost are discarded	Flag not set	All individuals were kept as controls for pairs of individuals who met minimum read criteria specified at a later point below.
**split_hosts_to_subgraphs.R**			
--pruneBlacklist	Prune all blacklisted reads from NGS phylogeny before ancestral state reconstruction	Flag not set	All reads were retained to enable investigation of potential contaminants from final output.
--splitsRule	Algorithm for identifying distinct subgraphs among NGS reads of one individual.	Sankoff algorithm	Phyloscanner default.
--kParam	K parameter in Sankoff cost matrix	20	Same as argument --sankoffK above.
--proximity Threshold	Distance parameter that determines when ancestral states return to unsampled individuals	0	This value was set so that ancestral state reconstruction did not depend on phylogenetic branch lengths.
--readCounts MatterOnZeroB ranches	Ancestral state reconstruction at parents of zero-branch lengths depends on read counts of children.	Flag set	Generally recommended when there is considerable variation in duplicate read counts.
**summary_statistics.R**			
No additional input arguments.			
**classify_relationships.R**			
No additional input arguments.			
**TransmissionSummary.R**			
--minThreshold	Summarize pairwise relationships only when they are not disconnected in at least this many potentially overlapping windows.	1	Summarize all pairwise relationships in csv summary file.
--distance Threshold	Summarize pairwise relationships only when their subgraph distances are below this threshold.	Inf	Summarize all pairwise relationships in csv summary file.
--allowMulti Trans	If absent, directionality is only inferred between two subjects when both subjects have one subgraph, and the two subgraphs are ancestral.	Flag set	Set so that directionality between two subjects was also inferred when one or both subjects had more than one subgraph, and all subgraphs of one subject were ancestral to all subjects of the other individual. Generally recommended for HIV.

**phsc.read.processed.phyloscanner.output.in.directory.Rscript**			
--trmw.min. reads	Minimum number of reads for both individuals in one window.	30	A value of 100 is usually recommended. Here we chose a smaller value in order to retain for analysis 75% of individuals for whom deep-sequence data was available. The low value reflects relatively poor deep sequencing quality of our data.
--trmw.min.tips	Minimum number of tips for both individuals in one window.	1	Retain all pairwise relationships; in particular we consider also individuals with no sampled viral diversity.
--trmw.close.brl	Distance parameter to classify subgraphs as phylogenetically close.	0.035 substitutions per site	Based on the couples' analysis reported in Figure 3A, this threshold is 0.025 substitutions per site. To ensure all potentially phylogenetically close pairs were found in stage 1 analysis (see Methods), this value was initially set to 0.035 substitutions per site, and then set to 0.025 substitutions per site in stage 2 analyses.
--trmw.distant. brl	Distance parameter to classify subgraphs as phylogenetically distant.	0.08 substitutions per site	Based on the couples' analysis reported in Figure 3A, this threshold is 0.05 substitutions per site. To ensure all potentially phylogenetically close pairs were found in stage 1 analysis (see Methods), this value was initially set to 0.08 substitutions per site, and then set to 0.05 substitutions per site in stage 2 analyses.
--trmw.min.neff	Minimum number of effectively non-overlapping windows.	3	The phylogenetic relationship between any pair of individuals was not evaluated when data was available from read alignments covering less than 750nt of the HIV-1 genome.
--prior.keff	Hyperparameter on number of effectively non-overlapping windows of one type.	1	Corresponds to flat prior.
--confidence.cut	Confidence threshold for classification.	0.5	We used a cut-off of 60% in stage 2 analyses, see Methods. To ensure all potentially phylogenetically close pairs were found in stage 1 analysis (see Methods), this value was initially set to 50%.
--rel.XXX	Flags to generate output classifications.	Flags set	All output classifications were included for comparative analyses, though this is typically not necessary.

Supplementary Table 3. Specification of deep-sequence phylogenetic analysis at the population-level: inference of transmission networks.

**Phyloscanner input parameter**	**Description**	**Value**	**Comments**
Input read file (no prefix)	Input read file	csv file	File specifying bam and reference files for each individual in one phyloscanner run. From stage 1 (see Methods), potential networks of phylogenetically close individuals were identified using the criteria in Figure 4 and Methods. To these networks, we added as controls reads from the next 10 phylogenetically closest individuals in stage 1 output. If networks contained only one of two partners who were known to have long-term sexual contact, the second person was added to the network. This resulted in 345 separate phyloscanner runs.
--merging-threshold-a	Genetic similarity threshold for merging similar reads	0	All distinct reads from one individual were kept to retain the entire sampled viral diversity for measuring subgraph relationships. This was a safe option to retain signal and incurred significant computational workload.
--min-read-count	Minimum count of unique reads so they were included in read alignments	1	All distinct reads from one individual were kept to retain the entire sampled viral diversity for measuring subgraph relationships. This was a safe option to retain signal and increased computational workload further.
--windows	Start and end coordinates of genomic windows	From 800 to 9400 in 25bp increments of 250bp windows	The window length was chosen so that 75% of mapped reads were retained in analysis. Windows were incremented by 25bp to capture 99% of mapped reads >250bp in at least one window. In comparison to bootstrap replicates on the

			same read alignment, overlapping windows accounted for uncertainty in read sequencing and the construction of read alignments.
--confidence.cut	Confidence threshold for classification.	0.6	See Methods.

Supplementary Table 4. Inference of phylogenetic transmission networks, sensitivity analyses.

	**Phylogenetically inferred transmission chains**		**Male-female pairs in inferred transmission chains**			
	Men and women	Links	Phylogenetic linkage highly supported		Phylogenetic linkage and source highly supported	
	(#)	(#)	(#)	(%)**	(#)	(%)***
**Subgraphs with fewer read counts are flagged as potential contaminants and discarded (--rawThreshold).**						
10 *	1334	888	376	42.3%	293	77.9%
20	1336	889	377	42.4%	290	76.9%
**Minimum number of reads for both individuals in one window (--trmw.min.reads).**						
10	1366	907	377	41.6%	293	77.7%
20	1362	914	378	41.4%	299	79.1%
30 *	1334	888	376	42.3%	293	77.9%
50	1307	867	374	43.1%	289	77.3%
**Threshold to collapse branches in deep-sequence phylogenies into polytomies (--multifurcation Threshold).**						
1e-05 *	1334	888	376	42.3%	293	77.9%
1e-03	1336	889	377	42.4%	294	78.0%
**Downsample reads to at most this number if more reads are present (--maxReadsPerHost).**						
30	1328	881	374	42.5%	298	79.7%
50 *	1334	888	376	42.3%	293	77.9%
100	1339	891	387	43.4%	311	80.4%
1000	1355	910	410	45.1%	326	79.5%
**Prune all blacklisted reads from NGS phylogeny before ancestral state reconstruction (--pruneBlacklist).**						
No *	1334	888	376	42.3%	293	77.9%
Yes	1329	884	375	42.4%	288	76.8%
**K parameter in Sankoff cost matrix (--sankoffK, --kParam)**						
10	1350	911	382	41.9%	296	77.5%
20 *	1334	888	376	42.3%	293	77.9%
**Proximity parameter in Sankoff cost matrix**						
0 substitutions per site *	1334	888	376	42.3%	293	77.9%
0.025 substitutions per site	1268	838	377	45.0%	290	76.9%
**Directionality is only inferred between two subjects when both subjects have one subgraph, and the two subgraphs are ancestral (--allowMultiTrans).**						
No	1330	885	376	42.5%	293	77.9%

Yes *	1334	888	376	42.3%	293	77.9%
**Ancestral state reconstruction at parents of zero-branch lengths depends on read counts of children (----readCounts MatterOnZeroBranches).**						
No	1337	891	378	42.4%	287	75.9%
Yes *	1334	888	376	42.3%	293	77.9%
**Distance parameter to classify subgraphs as phylogenetically close (--trmw.close.brl).**						
0.01 substitutions per site	1284	845	198	23.4%	153	77.3%
0.015 substitutions per site	1313	869	274	31.5%	218	79.6%
0.02 substitutions per site	1326	883	336	38.1%	258	76.8%
0.025 substitutions per site *	1334	888	376	42.3%	293	77.9%
0.03 substitutions per site	1331	887	423	47.7%	334	79.0%
0.035 substitutions per site	1338	891	452	50.7%	351	77.7%
0.04 substitutions per site	1339	892	471	52.8%	369	78.3%
**Confidence cut-off on phyloscanner linkage and direction scores**						
0.5	1334	888	434	48.9%	417	96.1%
0.55	1334	888	407	45.8%	356	87.5%
0.6 *	1334	888	376	42.3%	293	77.9%
0.65	1334	888	356	40.1%	244	68.5%
0.7	1334	888	328	36.9%	192	58.5%
0.75	1334	888	295	33.2%	130	44.1%
0.8	1334	888	258	29.1%	89	34.5%

* Input specification used in validation and central analysis. ** Proportion of links in inferred transmission chains. *** Proportion of male-female pairs between whom phylogenetic linkage was highly supported.

Supplementary Table 5. Inference of phylogenetically likely transmitters among couples, sensitivity analyses.

	**Phylogenetically linked male-female pairs in population sample with clinical evidence for transmission in one direction, including couples**				
	Inferred direction consistent	Direction not inferred	Inferred direction not consistent	False Discovery Rate	
	(#)	(#)	(#)	(point estimate)	(95% confidence interval)
**Subgraphs with fewer read counts are flagged as potential contaminants and discarded (--rawThreshold).**					
10 *	25	8	2	7.40%	[2.1%-23.4%]
20	18	7	2	10%	[2.8%-30.1%]
**Minimum number of reads for both individuals in one window (--trmw.min.reads).**					
10	19	8	3	13.60%	[4.7%-33.3%]
20	17	8	4	19%	[7.7%-40%]
30 *	25	8	2	7.40%	[2.1%-23.4%]
50	18	6	5	21.70%	[9.7%-41.9%]
**Threshold to collapse branches in deep-sequence phylogenies into polytomies (--multifurcation Threshold).**					
1e-05 *	25	8	2	7.40%	[2.1%-23.4%]
1e-03	17	8	2	10.50%	[2.9%-31.4%]
**Downsample reads to at most this number if more reads are present (--maxReadsPerHost).**					
30	19	5	3	13.60%	[4.7%-33.3%]

50 *	25	8	2	7.40%	[2.1%-23.4%]
100	17	8	2	10.50%	[2.9%-31.4%]
1000	21	7	3	12.50%	[4.3%-31%]
**Prune all blacklisted reads from NGS phylogeny before ancestral state reconstruction (--pruneBlacklist).**					
No *	25	8	2	7.40%	[2.1%-23.4%]
Yes	25	8	2	7.40%	[2.1%-23.4%]
**K parameter in Sankoff cost matrix (--sankoffK, --kParam)**					
10	19	6	2	9.50%	[2.7%-28.9%]
20 *	25	8	2	7.40%	[2.1%-23.4%]
**Proximity parameter in Sankoff cost matrix**					
0 substitutions per site *	25	8	2	7.40%	[2.1%-23.4%]
0.025 substitutions per site	17	7	3	15%	[5.2%-36%]
**Directionality is only inferred between two subjects when both subjects have one subgraph, and the two subgraphs are ancestral (--allowMultiTrans).**					
No	17	8	2	10.50%	[2.9%-31.4%]
Yes *	25	8	2	7.40%	[2.1%-23.4%]
**Ancestral state reconstruction at parents of zero-branch lengths depends on read counts of children (----readCounts MatterOnZeroBranches).**					
No	18	7	2	10%	[2.8%-30.1%]
Yes *	25	8	2	7.40%	[2.1%-23.4%]
**Distance parameter to classify subgraphs as phylogenetically close (--trmw.close.brl).**					
0.01 substitutions per site	10	6	1	9.10%	[0.5%-37.7%]
0.015 substitutions per site	14	5	2	12.50%	[3.5%-36%]
0.02 substitutions per site	16	10	1	5.90%	[0.3%-27%]
0.025 substitutions per site *	25	8	2	7.40%	[2.1%-23.4%]
0.03 substitutions per site	21	7	3	12.50%	[4.3%-31%]
0.035 substitutions per site	21	8	4	16%	[6.4%-34.7%]
0.04 substitutions per site	22	8	4	15.40%	[6.2%-33.5%]
**Confidence cut-off on phyloscanner linkage and direction scores**					
0.5	31	1	6	16.20%	[7.7%-31.1%]
0.55	28	5	3	9.70%	[3.3%-24.9%]
0.6 *	25	8	2	7.40%	[2.1%-23.4%]
0.65	23	10	2	8%	[2.2%-25%]
0.7	17	17	1	5.60%	[0.3%-25.8%]
0.75	10	20	1	9.10%	[0.5%-37.7%]
0.8	8	19	0	0%	[0%-32.4%]

Supplementary Table 6. Inference of phylogenetically likely transmitters in the population-based sample, sensitivity analyses.

	**Phylogenetically linked male-female pairs in population sample with clinical evidence for transmission in one direction, including couples**				
	Inferred direction consistent	Direction not inferred	Inferred direction not consistent	False Discovery Rate	
	(#)	(#)	(#)	(point estimate)	(95% confidence interval)

**Subgraphs with fewer read counts are flagged as potential contaminants and discarded (--rawThreshold).**					
10 *	46	16	9	16.4%	[8.9%-28.3%]
20	48	16	8	14.3%	[7.4%-25.7%]
**Minimum number of reads for both individuals in one window (--trmw.min.reads).**					
10	49	13	8	14.0%	[7.3%-25.3%]
20	45	15	12	21.1%	[12.5%-33.3%]
30 *	46	16	9	16.4%	[8.9%-28.3%]
50	48	14	12	20.0%	[11.8%-31.8%]
**Threshold to collapse branches in deep-sequence phylogenies into polytomies (--multifurcation Threshold).**					
1e-05 *	46	16	9	16.4%	[8.9%-28.3%]
1e-03	46	16	9	16.4%	[8.9%-28.3%]
**Downsample reads to at most this number if more reads are present (--maxReadsPerHost).**					
30	49	11	11	18.3%	[10.6%-29.9%]
50 *	46	16	9	16.4%	[8.9%-28.3%]
100	46	15	10	17.9%	[10%-29.8%]
1000	54	14	10	15.6%	[8.7%-26.4%]
**Prune all blacklisted reads from NGS phylogeny before ancestral state reconstruction (--pruneBlacklist).**					
No *	46	16	9	16.4%	[8.9%-28.3%]
Yes	46	17	8	14.8%	[7.7%-26.6%]
**K parameter in Sankoff cost matrix (--sankoffK, --kParam)**					
10	48	13	10	17.2%	[9.6%-28.9%]
20 *	46	16	9	16.4%	[8.9%-28.3%]
**Proximity parameter in Sankoff cost matrix**					
0 substitutions per site *	46	16	9	16.4%	[8.9%-28.3%]
0.025 substitutions per site	45	16	10	18.2%	[10.2%-30.3%]
**Directionality is only inferred between two subjects when both subjects have one subgraph, and the two subgraphs are ancestral (--allowMultiTrans).**					
No	46	16	9	16.4%	[8.9%-28.3%]
Yes *	46	16	9	16.4%	[8.9%-28.3%]
**Ancestral state reconstruction at parents of zero-branch lengths depends on read counts of children (----readCounts MatterOnZeroBranches).**					
No	49	14	8	14.0%	[7.3%-25.3%]
Yes *	46	16	9	16.4%	[8.9%-28.3%]
**Distance parameter to classify subgraphs as phylogenetically close (--trmw.close.brl).**					
0.01 substitutions per site	26	8	5	16.1%	[7.1%-32.6%]
0.015 substitutions per site	35	7	9	20.5%	[11.2%-34.5%]
0.02 substitutions per site	43	16	8	15.7%	[8.2%-28%]
0.025 substitutions per site *	46	16	9	16.4%	[8.9%-28.3%]
0.03 substitutions per site	53	15	12	18.5%	[10.9%-29.6%]
0.035 substitutions per site	56	19	12	17.6%	[10.4%-28.4%]
0.04 substitutions per site	59	20	13	18.1%	[10.9%-28.5%]
**Confidence cut-off on phyloscanner linkage and direction scores**					
0.5	60	2	19	24.1%	[16%-34.5%]
0.55	52	11	13	20.0%	[12.1%-31.3%]
0.6 *	46	16	9	16.4%	[8.9%-28.3%]
0.65	44	18	8	15.4%	[8.0%-27.5%]
0.7	37	26	6	14.0%	[6.6%-27.3%]
0.75	25	32	3	10.7%	[3.7%-27.2%]
0.8	20	31	1	4.8%	[0.2%-22.7%]

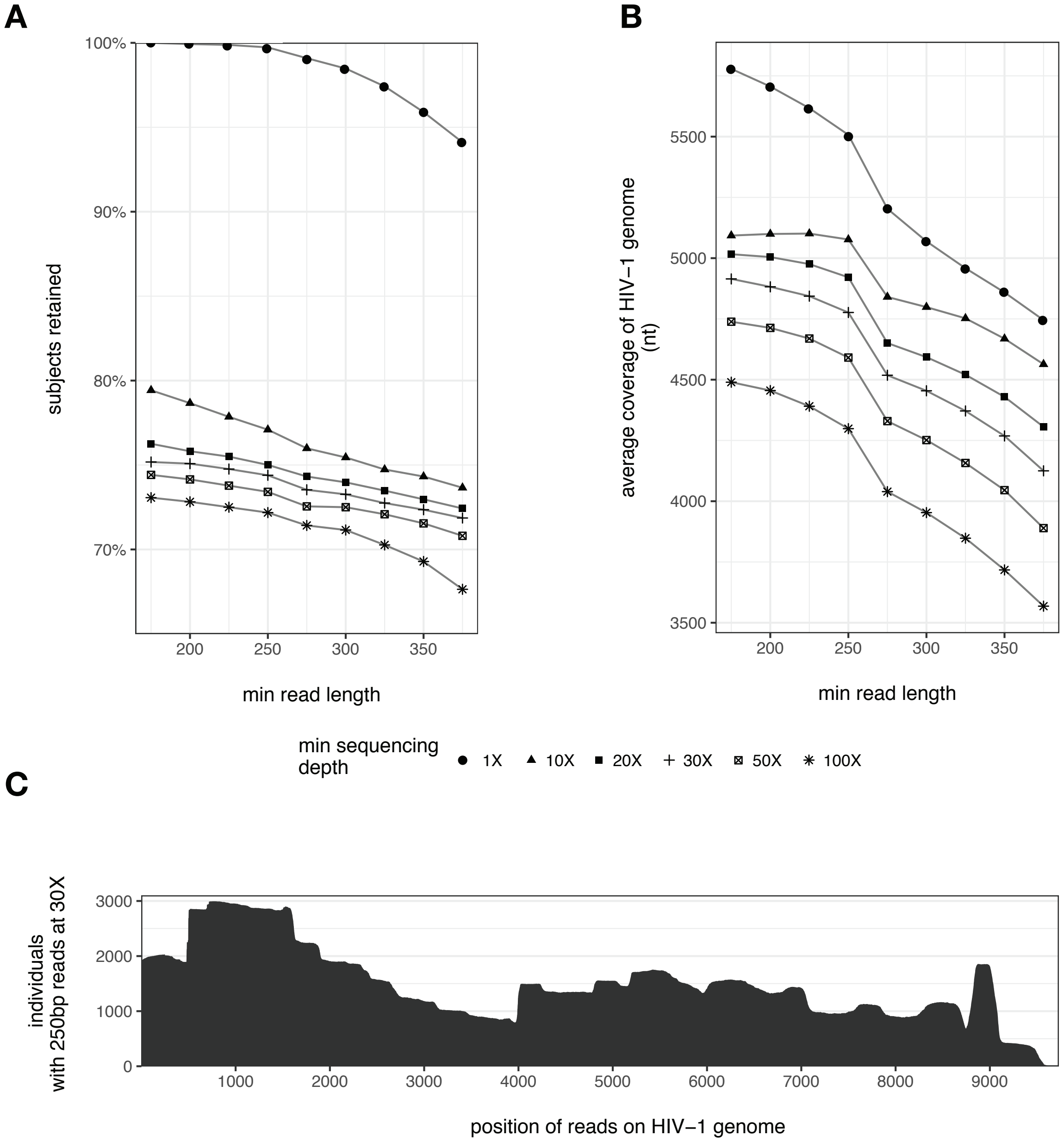

Supplementary Figure 1. Characteristics of deep sequencing output of HIV-1 samples from Rakai District, Uganda. Deep sequencing was performed in high throughput on *Illumina* MiSeq and HiSeq instruments after automated extraction of viral RNA and amplification with a universal HIV-1 primer set[4]. Reads were mapped against de-novo reference sequences with shiver[5]. (**A**) The number of study subjects with deep sequencing output over at least 750nt of the HIV-1 genome decreased relatively steadily as a function of stricter requirements on the minimum sequencing depth at any position (symbols), and as a function of stricter requirements on the minimum length of reads increased (x-axis). 773 individuals were poorly sequenced with a read depth less than 10X. Approximately 3,000 individuals were retained at a minimum read depth of 10X to 30X. Slightly more individuals were lost to further analysis when the minimum read length was increased from 250nt to 275nt, as compared to other 25nt increases in minimum read length. (**B**) Coverage of the HIV-1 genome dropped more markedly between a minimum read length of 250nt and 275nt. This drop corresponded to situations when one of the two reads of a RNA template could be almost fully sequenced (length >250nt), but the second read failed to be

sequenced in the opposite direction such that the two mates did not overlap, and did not produce a read of at least 275nt. We therefore set the minimum required read length to 250nt. (**C**) Considering individuals that could be deep sequenced at 30X with reads of at least 250nt over a minimum coverage of 750nt of the HIV-1 genome, most had reads covering the HIV-1 *gag* gene. Overall, in comparison to clinical samples from European HIV-1 subtype B patients, sequencing output on our African samples was of lower quality[6]. The minimum length of reads (250bp) was set lower compared to deep-sequence phylogenetic analyses on European samples (350bp), and chosen as described above by trading off against individuals retained. In general, phylogenetic reconstruction accuracy decays strongly with shorter read lengths[7], suggesting that a stronger phylogenetic signal into HIV-1 transmission networks could likely have been obtained if data had been of similar quality as obtained in Europe.

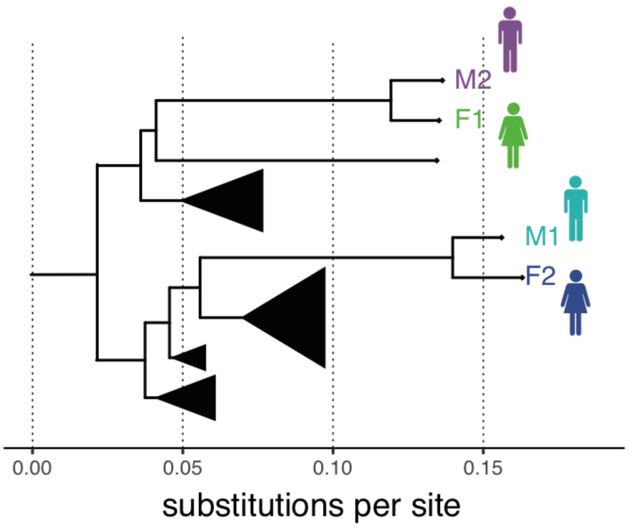

Supplementary Figure 2. Phylogenetic analysis from consensus sequences of the four selected individuals for whom deep-sequence phylogenetic analysis is illustrated in figure 1.

Inferring HIV-1 transmission networks and sources of epidemic spread in Africa with deep-sequence phylogenetic analysis

Supplementary Note 1. Calculation of phyloscanner scores

Consider the following example in which two individuals i and j had reads that overlapped ten genomic windows. Following the specification used on Rakai data, windows were 250nt long and slid by 25nt increments across the HIV-1 genome, with coordinates relative to HXB2 as shown in Supplementary Figure 3.

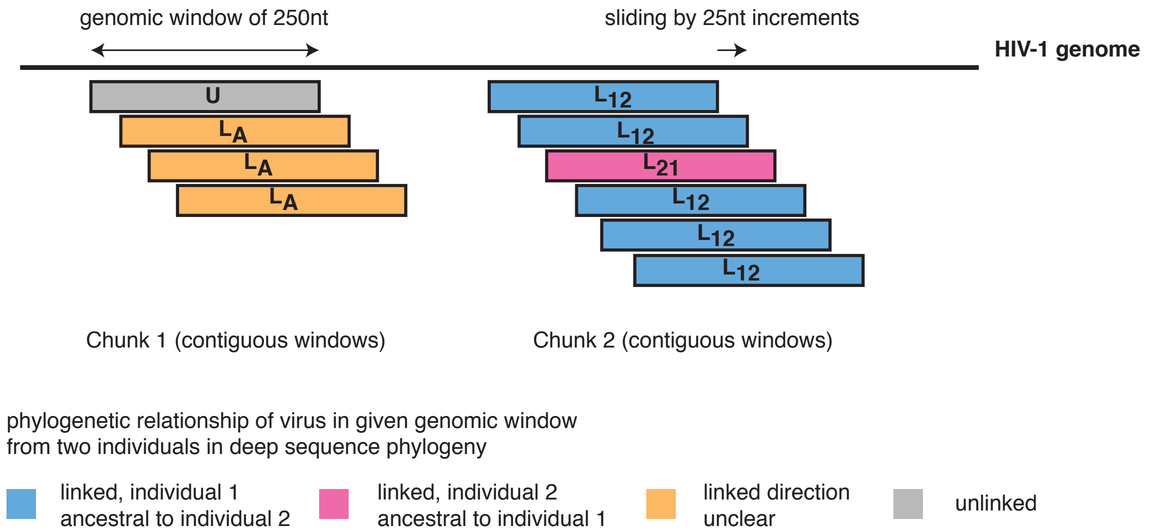

Supplementary Figure 3. Overlapping genomic windows. Phylogenetic trees were reconstructed for many genomic windows across the HIV-1 genome, which incremented by 25bp. If reads from individuals did not meet minimum quality criteria in a window, pairwise phylogenetic relationships between that and any other individual were not performed, leading to missing data. A series of contiguous pairwise phylogenetic relationships is referred to as a chunk. Subgraph topologies are indicated in colours.

For each window, phyloscanner constructs read alignments of 250nt in length, uses RAxML to infer corresponding deep-sequence phylogenies, identifies within-host subgraphs in these phylogenies, and characterizes their distance and topological relationship[8]. As illustrated in colours, for each genomic window, pairs are assigned to one of the five categories:

Symbol	Description	Definition (see Methods)
U	Phylogenetically unlinked.	$A_{ij} = 0$ or $\Delta_{ij} > 0.05$ substitutions per site
G	Greyzone phylogenetically linked.	$A_{ij} = 1, \Delta_{ij} \in [0.025 - 0.05$ substitutions per site]

L_{12}	Phylogenetically linked, with subgraphs from 1 ancestral to those of 2	$A_{ij} = 1, \Delta_{ij} < 0.025$ substitutions per site, $P_{ij} \geq 1$, $P_{ji} = 0$
L_{21}	Phylogenetically linked, with subgraphs from 2 ancestral to those of 1	$A_{ij} = 1, \Delta_{ij} < 0.025$ substitutions per site, $P_{ij} = 0$, $P_{ji} \geq 1$
L_A	Phylogenetically linked, with intermingled or sibling subgraphs,	$A_{ij} = 1, \Delta_{ij} < 0.025$ substitutions per site, $P_{ij} \geq 1$. $P_{ji} \geq 1$ or $A_{ij} = 1, \Delta_{ij} < 0.025$ substitutions per site, $P_{ij} = 0$, $P_{ji} = 0$

Observed pairwise relationships are then counted while adjusting for overlap in read alignments with the following algorithm.

Algorithm

Denote the unadjusted counts in order by $\tilde{k}_U, \tilde{k}_G, \tilde{k}_{ij}, \tilde{k}_{ji}, \tilde{k}_A$, and their sum by $\tilde{n}$.

1. Identify genomic chunks c of consecutive genomic windows in which i and j have reads.
2. Calculate the effective number of non-overlapping windows in chunk c,
$$n_c = \frac{\max_{w \in c}(E_w) + 1 - \min_{w \in c}(S_w)}{E_w + 1 - S_w}$$
where S_w, E_w are the first and last nucleotide positions in window w respectively. The numerator is the length of chunk c, and the denominator is the length of one window.
3. Calculate the effective number of non-overlapping windows in chunk c that are of type t,
$$k_{tc} = \frac{\tilde{k}_{tc}}{\tilde{n}_c / n_c}$$
where $\tilde{k}_{tc}$ is the number of overlapping windows of type t in chunk c, and $\tilde{n}_c$ is the number of overlapping windows in chunk c.

Sum to obtain $n = \sum_c n_c$, and $k_t = \sum_c k_{tc}$ for all relationship types t.

In the example above, there are two chunks. Chunk 1 consists of 4 read alignments spanning 325nt, and contributes 1.3 effectively independent observations. Similary, chunk 2 consists of 6 read alignments spanning 375nt, and contributes 1.5 effectively independent observations:

Chunk	Genomic windows	Length (nt)	Effectively independent observations
Chunk 1	4	325	1.3 = 325/250
Chunk 2	6	375	1.5 = 375/250
Total	10	700	2.8

The adjusted counts are:

Chunk	Adjusted counts				
	L_{12}	L_{21}	L_A	G	D
Chunk 1	0	0	$^3/_4$ * 1.3	0	$^1/_4$ * 1.3
Chunk 2	$^5/_6$ * 1.5	$^1/_6$ * 1.5	0	0	0
Total	1.25	0.25	0.975	0	0.325

The relative phylogenetic evidence for i and j being epidemiologically unlinked, and infection from i to j, and vice versa were thus:

Strength of phylogenetic evidence (point estimates)		
$\hat{\mu}_{ij}$	$\hat{\lambda}_{ij}$	$\hat{\delta}_{ij}$
0.325/2.8 = 0.12	(1.25+0.975+0.25)/2.8 = 0.88	1.25/(1.25+0.25) = 0.83

Inferring HIV-1 transmission networks and sources of epidemic spread in Africa with deep-sequence phylogenetic analysis

Supplementary Note 2. Inferring phylogenetic linkage from deep-sequence data compared to consensus sequences

We compared the agreement between phylogenetic linkage analysis from deep-sequence data and consensus sequence data on the couples' data set ($n = 331$ couples). Our primary aim was to assess concordance in estimating phylogenetic linkage on an empirical data set in which linkage is relatively unambiguous to characterize.

Deep-sequence phylogenetic analysis of couples

Supplementary Figure 4 summarizes deep-sequence viral phylogenetic analysis on the couples. Supplementary Figure 4A shows the number of deep-sequence phylogenies that were evaluated per couple (y-axis), after adjusting for overlap in read alignments. Subgraph topologies between spouses are indicated in colours. Couples did not necessarily both have sequencing output in any one genomic window, and for this reason the number of phylogenetic repeat observations per couple varied considerably (varying heights of bars). Supplementary Figure 4B illustrates median subgraph distances (dots) and empirical 95% confidence interval of subgraph distances per couple, where the median was taken across deep-sequence phylogenies, and after phylogenetic distances were rescaled to reflect typical distances observed in the HIV-1 *pol* gene (see Methods). Very large confidence intervals indicate that in some phylogenies, the subgraphs of couples were very close while in other phylogenies, their subgraphs were highly divergent, which may indicate read contamination, artifacts in tree reconstruction, recombination, or the presence of divergent and cocirculating viral variants in one or both individuals. Supplementary Figure 4C shows the linkage score $\hat{\lambda}_{ij}$ along with Bayesian 95% credibility intervals, which is based on subgraph distances and subgraph topologies as described in Methods. Supplementary Figure 4D shows the direction score $\hat{\delta}_{ij}$ along with corresponding Bayesian 95% credibility intervals.

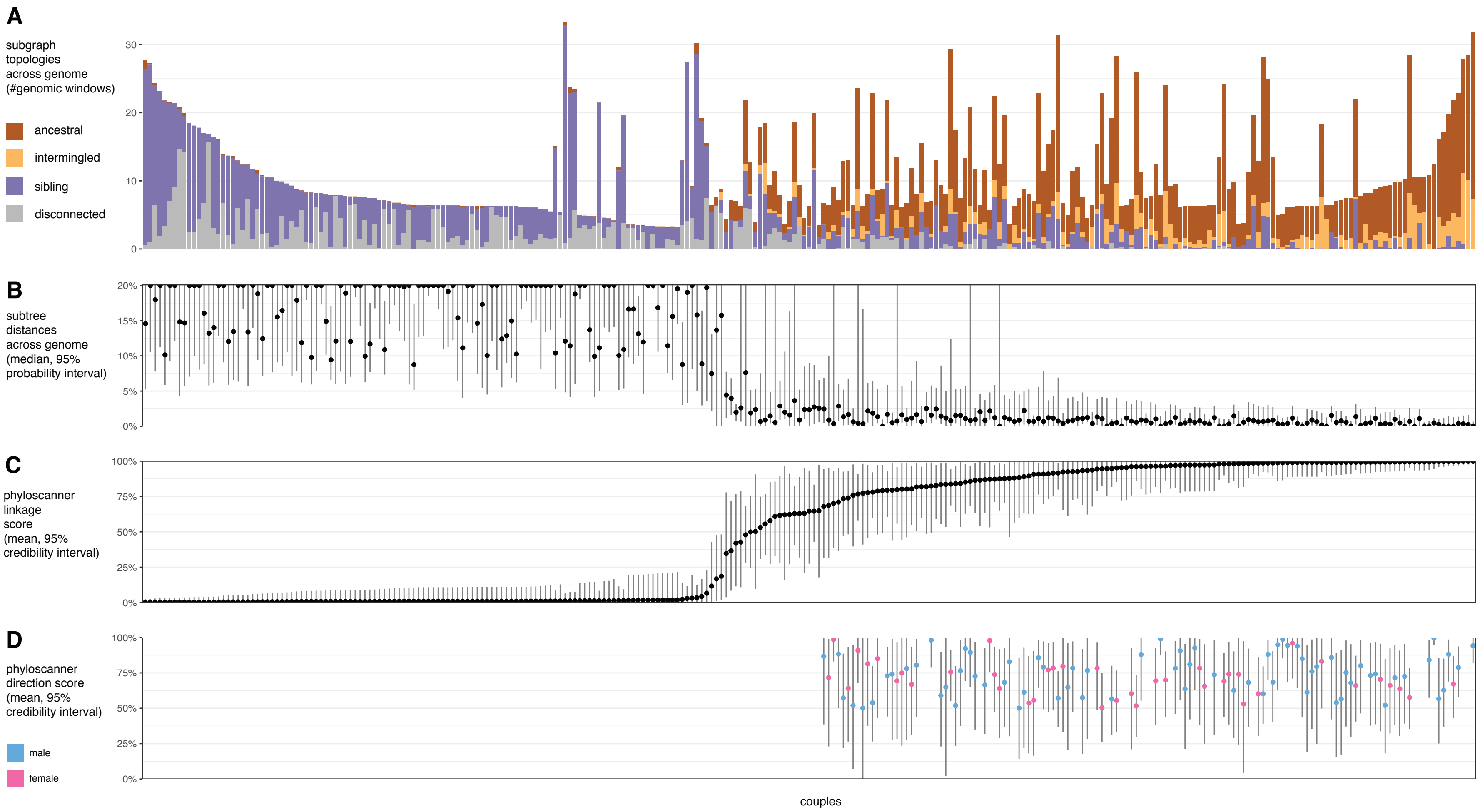

Supplementary Figure 4. Viral phylogenetic relationships among 331 couples in Rakai District, Uganda, inferred from deep-sequence data. Please see text for details.

We further investigated wether one or both spouses harboured highly divergent virus, which could indicate dual infection or recombination. To this end, we catalogued for each spouse subgraphs that were highly divergent from the majority subgraph that contained most reads of that spouse in any phylogeny. Within-host subgraphs were considered highly divergent if they were more than 0.05 substitutions per site apart from the majority subgraph, based on the results shown in Figure 3A. Divergent subgraphs were further characterized by read number (1, 2-9, 10+). Supplementary Figure 5 illustrates that spouses frequently had divergent subgraphs of just one read, which could be due to read contamination and/or artifacts in tree reconstruction. 42 of 331 couples (12.7%) had at least one spouse with divergent subgraphs of at least 2 reads in more than 33% of deep-sequence phylogenies (after adjusting counts for overlap in genomic windows as described in Supplementary Note 1). 12 (3.6%) of 331 couples had divergent subgraphs of at least 2 reads in more than 66% of deep-sequence phylogenies.

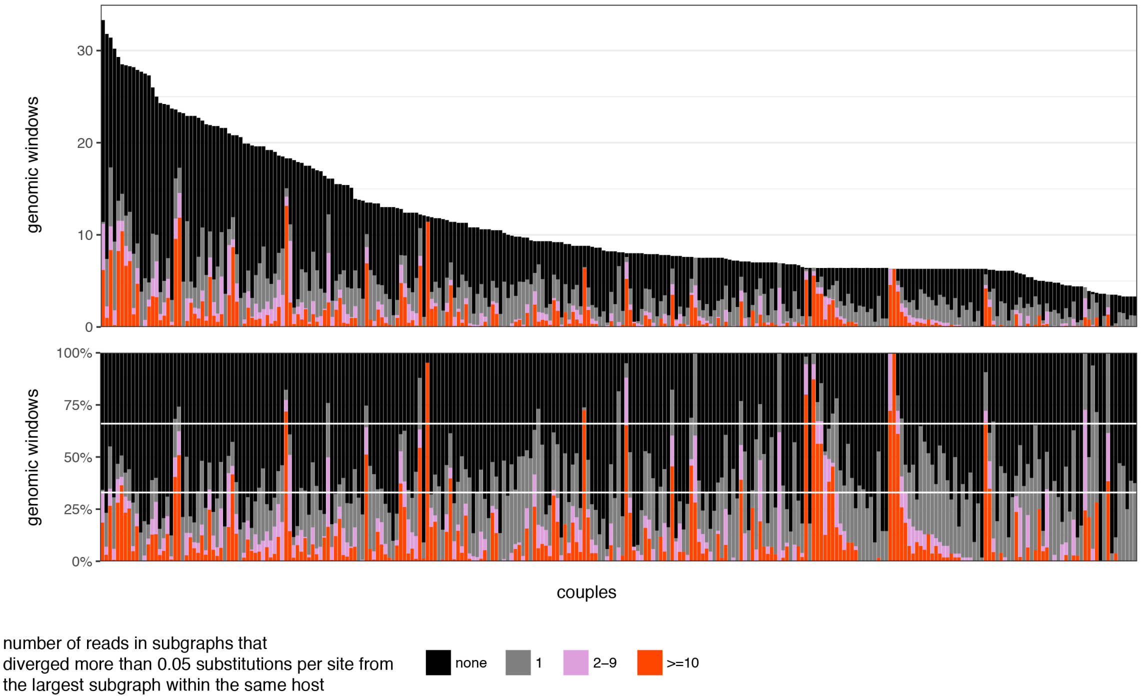

Supplementary Figure 5. Counts and frequency of divergent virus within spouses. For each of the 331 couples with deep-sequence data (x-axis), deep-sequence phylogenies with divergent subgraphs in one or both spouses were counted, and are shown by the number of reads within them (colour). The number was adjusted for overlap of genomic windows (Supplementary Note 1). Overall, spouses frequently had divergent clades of just one read, indicative of read contamination. For the 6 couples that were classified linked using deep sequencing data but not linked using consensus sequences, at least one spouse had divergent subgraphs in at least 33% of (effective) deep-sequence phylogenies.

Generation of consensus sequences

Consensus sequences were generated from mapped read alignments by determining the majority nucleotide call at each base position of the HIV-1 genome, as described in Ref.[6].

Concordance between phylogenetic distances in deep-sequence phylogenies with genetic distances between consensus sequences

For consensus sequences, genetic distances were calculated under three evolutionary models, Tamura-Nei-1993, Tamura-Nei-1993 with Gamma correction, and raw genetic distance using the ape package in R[9,10]. Phylogenetic linkage classification of the spouses from consensus sequences was identical under all three distance matrices, and results are reported for raw genetic distances.

Supplementary Figure 6 illustrates the bivariate relationship between the raw genetic distances obtained from consensus sequences versus median subgraph distances obtained from deep-sequence data. Shown in orange are the 42 couples for whom one or both individuals had divergent subgraphs of at least 2 reads in more than 33% of deep-sequence phylogenies. Overall, the two distance measures were highly correlated (Spearman log rank correlation coefficient $\rho = 0.87$).

To describe the relationship between both distance measures, polynomial splines were fitted to the data after excluding 42 couples with divergent subgraphs and 32 couples with identical subgraphs. A polynomial spline of order 4 provided the best fit and is shown as a line in Supplementary Figure 6.

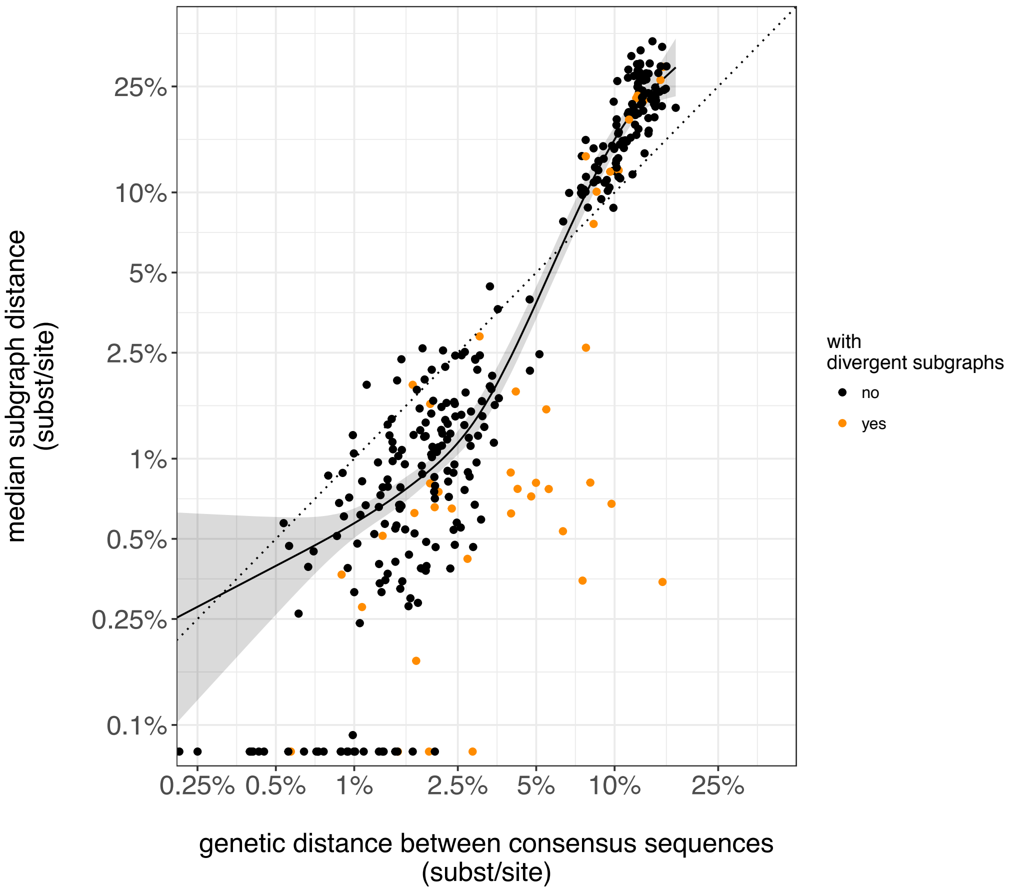

Supplementary Figure 6. Concordance between median subgraph distances of couples in deep-sequence phylogenies and genetic distances between consensus sequences. Data from 311 couples were available to compare the two distance measures. For each couple, deep-sequence phylogenies were rescaled to account for variation in mutation rates across the genome, and the subgraph distance between couples was determined in all their deep-sequence phylogenies. Genetic distances were determined as described in the text. The plots show the bivariate relationship between median subgraph distances (with median taken over all phylogenies of a couple) and genetic distance between consensus sequences. Couples for whom one or both spouses had divergent subgraphs are shown in orange. For visualization purposes, couples with identical deep-sequence reads in 50% of deep-sequence phylogenies are shown on a horizontal line below 0.1% substitutions per site. The curve shows the best-fitting polynomial transformation between the two distance measures. The two distance measures were highly correlated (Spearman log rank correlation coefficient ρ = 0.87).

Phylogenetic linkage classification

Using deep-sequence data, couples were classified as phylogenetically linked as fully described in the main text by:

- identifying most likely transmission chains in the whole population sample,
- determining if couples were directly linked in a transmission chain,
- classifying a couple as phylogenetically linked with high support when the linkage score exceeded a particular threshold, here 60% ($\hat{\lambda}_{ij} > 0.6$; see Methods).

Using consensus sequences, couples were classified as phylogenetically linked by:

- identifying if the spouse was the genetically closest individual in the whole population sample,
- classifying a couple as phylogenetically linked when their genetic distance did not exceed a particular threshold.

The distance threshold for classifying couples as phylogenetically linked from consensus sequences was based on the transformation function shown in Supplementary Figure 6. Supplementary Table 7 lists corresponding distance thresholds, and further investigation was based on a threshold of 0.025 substitutions per site on subgraph distances (see results in Figure 3A) and the corresponding threshold of 0.041 substitutions per site on genetic distances between consensus sequences.

Supplementary Table 7. Conversion between subgraph distances in scaled deep-sequence phylogenies and genetic distances between consensus sequences

	**substitutions per site scaled for HIV-1 *pol* gene**								
**subgraph distances in scaled deep-sequence phylogenies**	0.01	0.015	0.02	0.025	0.03	0.035	0.04	0.045	0.05
**genetic distance between consensus sequences**	0.022	0.031	0.036	0.041	0.044	0.048	0.051	0.054	0.056

Between the two approaches, phylogenetic linkage classification agreed for 297/331 (89.7%) of couples (Supplementary Table 8). 26 couples were classified linked using consensus sequences but not linked using deep sequencing data. Of those, linkage in 5 couples was excluded because in the overall transmission network, linkage with other individuals was more likely based on our phylogenetic data; linkage in 3 couples was excluded because one of the two individuals had divergent subgraphs; and linkage in 16 couples was excluded because support for phylogenetic linkage was intermediate but not high enough, with $\hat{\lambda}_{ij}$ between 40-60%. This left 2 couples for whom we could not find an immediate explanation why consensus sequences indicated linkage but deep-sequence data did not. For all 8 couples that were classified linked using deep sequencing data but not linked using consensus sequences, at least one spouse had divergent subgraphs in at least 33% of deep-sequence phylogenies. Supplementary Figure 7 shows the couples for whom the two phylogenetic analyses disagreed, confirming that these couples were at the border of the classification

thresholds that we used in our analysis. Supplementary Figure 8 illustrates subgraph distances, subgraph topologies and within-host subgraph divergence for 6 of the 8 couples that were classified as linked only when using deep sequencing data. Most couples (except B and F) had highly variable subgraph distances across the genome. These tended to coincide with genomic regions without divergent within-host subgraphs, suggesting that the closely related subgraph still present in their partner was either not sequenced, or lost in the quasi-species. In couples B and F, the closely related subgraphs were sequenced in both spouses, implying small subgraph distances across the sequenced genome but large genetic distance from consensus sequences.

Supplementary Table 8. Comparison of phylogenetic linkage classification based on deep sequencing data and consensus sequences among 331 couples from Rakai District, Uganda.

**Phylogenetic linkage classification among long-term sexual partners**			**Phyloscanner probability of phylogenetic linkage** *			**Proportion of deep-sequence phylogenies with divergent subgraphs in at least one spouse** **		
			(mean and 95% empirical confidence interval across couples)			(mean and 95% empirical confidence interval across couples)		
	Deep sequence			Deep sequence			Deep sequence	
Consensus sequence	Not linked	Linked	Consensus sequence	Not linked	Linked	Consensus sequence	Not linked	Linked
Not linked	129	8	Not linked	3% [0%-53%]	77% [67%-90%]	Not linked	12% [0%-61%]	68% [38%-96%]
Linked	26	168	Linked	53% [32%-96%]	90% [67%-100%]	Linked	12% [0%-47%]	13% [0%-64%]
			* Posterior mode estimate for being phylogenetically linked, see Methods.			** Divergent subgraphs in one individual were defined as subgraphs more than 0.05 substitutions per site apart from the individual's main subgraph, which contained at least 2 unique reads.		

In summary, we found that phylogenetic linkage estimates from consensus sequences and deep-sequence reads were strongly concordant, in 297/331 (89.7%) of couples. For the majority of the remaining cases, we either found intermediate but not high support for linkage in deep-sequence phylogenies (16/34 (47.1%) of couples), or evidence of highly divergent subgraphs in one or both individuals (11/34 (32.4%) of couples), which typically implied high support for phylogenetic linkage based on deep-sequence reads.

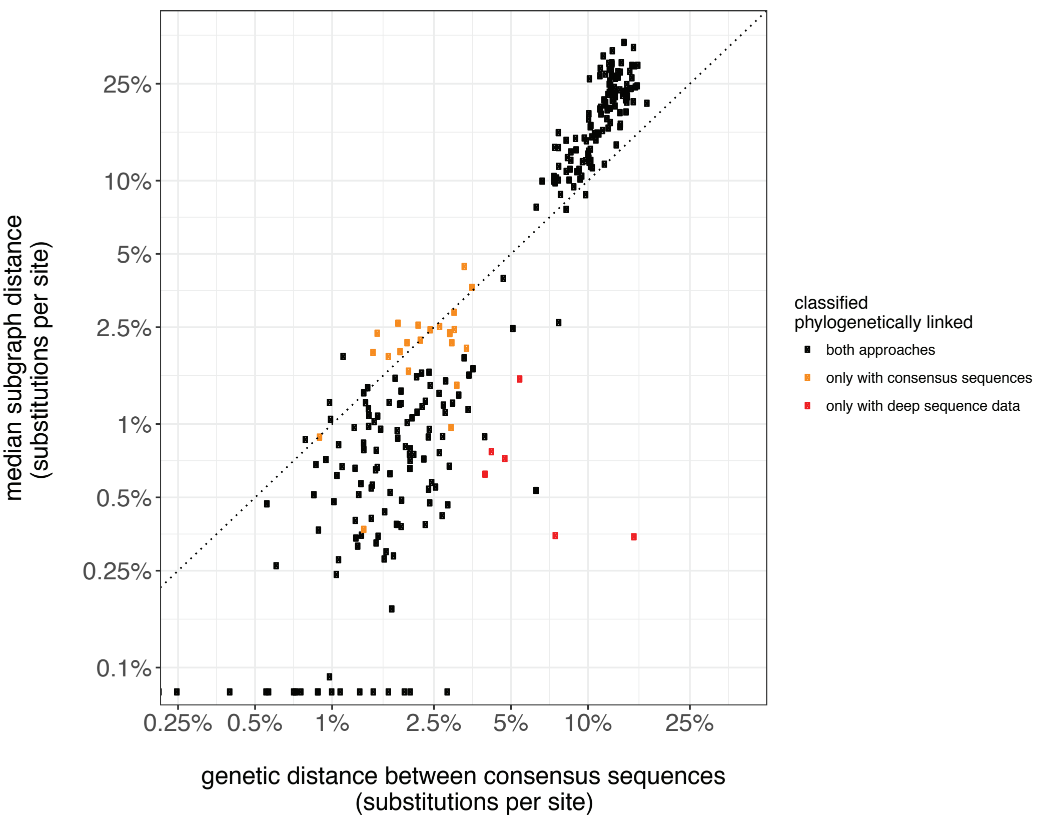

Supplementary Figure 7. Couples for whom linkage classification based on consensus and deep-sequence analysis disagreed. The dotted line shows y=x.

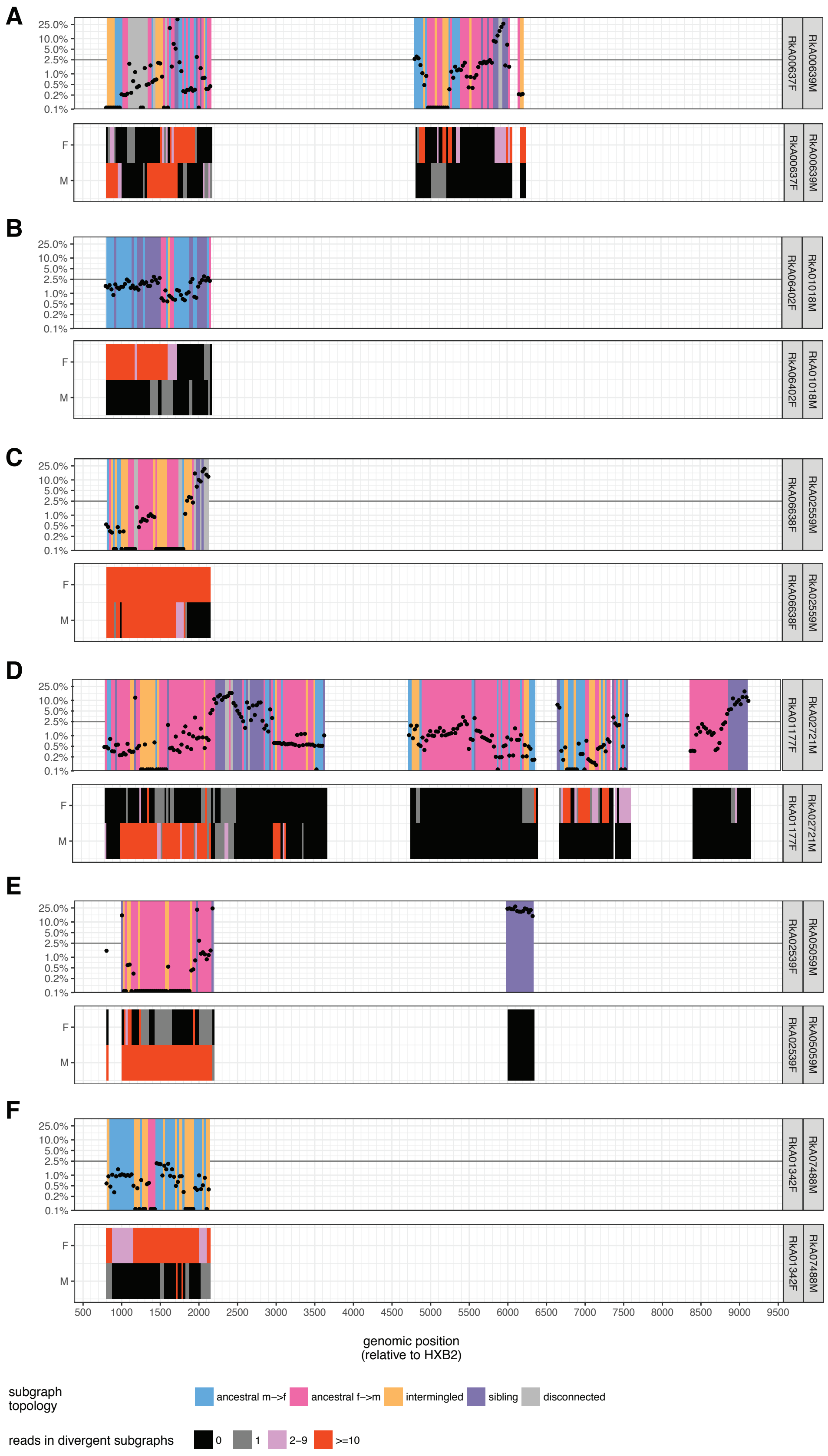

Supplementary Figure 8. Subgraph distance, topology and divergence among couples that were phylogenetically linked using deep sequencing data, but not linked using consensus sequences.

Inferring HIV-1 transmission networks and sources of epidemic spread in Africa with deep-sequence phylogenetic analysis

Supplementary Note 3. Error rates in inferring phylogenetic linkage from deep-sequence data in the population-based sample

HIV-1 is predominantly sexually transmitted, and extremely rarely sexually transmitted between women[11]. This allowed us to characterize error rates in phylogenetic inference of direct transmission between males and females in the population sample.

Denote the number of phylogenetically linked female-female pairs by L_{ff}. For S_f sequenced females and S_m sequenced males, there are S_f*(S_f-1)/2 pairs of sequenced females, and the probability of inferring a phylogenetically linked female-female pair is

$$\frac{L_{ff}}{S_f * (S_f - 1)/2}.$$

If we assume that the probability of incorrectly inferring a phylogenetically linked male-female pair is the same as the above probability of inferring a phylogenetically linked female-female pair, the number of linked male-female pairs between whom transmission did not occur can thus be estimated by

$$\hat{F}^C_{mf} = \frac{L_{ff}}{S_f * (S_f - 1)/2} * S_f * S_m,$$

Suppose that L_{mf} male-female pairs were inferred to be phylogenetically linked. An estimate of the false discovery rate is

$$\hat{\rho}^C_{mf} = \frac{\hat{F}^C_{mf}}{L_{mf}}.$$

This probably overestimates the true false discovery rate because two individuals would have to be missing from the sequence sample to incorrectly infer phylogenetic linkage in a male-female pair, where only one male would have to be missing from the sequence sample to incorrectly infer phylogenetic linkage in a female-female pair. Supplementary Table 9 lists estimates of $\hat{\rho}^C_{mf}$ for a range of distance thresholds.

Supplementary Table 9. Estimated error rates in inferring direct transmission from deep sequencing data in Rakai, Uganda.

	**Threshold on subgraph distances to define phylogenetically linked individuals in combination with subgraph topology** (in substitutions per site)					
	**0.01**	**0.015**	**0.02**	**0.025**	**0.03**	**0.035**
**Phylogenetically linked female-female pairs**	25	43	61	80	99	117
**Phylogenetically linked male-female pairs between whom transmission did not occur (estimated)**	42	72	102	133	165	195
**Phylogenetically linked male-female pairs**	198	274	336	376	423	452
**False discovery rate ***	21%	26.10%	30.20%	35.40%	39%	43.10%

* Assuming equal false positive rates among female-female pairs and male-female pairs, see text.

Inferring HIV-1 transmission networks and sources of epidemic spread in Africa with deep-sequence phylogenetic analysis

Supplementary Note 4: Limitations in inferring the direction of transmission from deep-sequence data

We investigated why the direction of transmission was incorrectly inferred with the phyloscanner method in the nine cases reported in table 2. Given the small number of pairs for whom the direction of transmission was inconsistent with clinical data, this analysis remains largely descriptive. The validation analysis was based on phylogenetically linked pairs of individuals with clinical evidence for the direction of transmission based on seroconversion dates and CD4 cell count measurements, and for whom phylogenetical linkage was inferred with high support. Prior to validation, the selection criteria were specified as follows:

- **Seroconversion data**. Partner 1 tested negative while partner 2 tested positive at or before the same time. Subsequently, partner 1 tested positive. Assuming that transmission occurred between the two individuals, seroconversion data indicates transmission from partner 2 to partner 1.
- **CD4 data**. Partner 1 had first CD4 measurement >800 cells per mm3 within two years of diagnosis, while partner 2 had a CD4 measurement <400 cells per mm3 within two years of diagnosis of partner 1. Assuming that transmission occurred between the two individuals, CD4 data indicates transmission from partner 2 to partner 1.

Detailed epidemiological and phylogenetic characterization of the validation data set.

Detailed timelines on seroconversion dates, CD4 counts, sequencing dates and phyloscanner output for the 55 phylogenetically linked pairs in the validation panel are shown in Supplementary Figures 9–12.

Post-hoc evaluation of the selection criteria by which the validation data set was formed.

We examined potential limitations in these selection criteria. For 36 phylogenetically linked pairs, data on the direction of transmission was available from the seroconversion history, and the direction of transmission could be inferred with phyloscanner in 31 pairs. In 16/31 of pairs, the time between the first positive date of the (epidemiologically inferred) source case and the (epidemiologically inferred) recipient was less than 1 month. Considering limited sensitivity of HIV-1 tests in early infection, it was thus possible (though not very likely) that infection could have occurred the other way round in these pairs. However, the odds ratio for incorrect phylogenetic inference among pairs with very small differences in first positive and last negative dates versus those with larger differences was

$$(2/14)/(2/13) = 0.93$$

and not significant (Fisher exact test).

For 35 phylogenetically linked pairs, data on the direction of transmission was available from the CD4 count history, and the direction of transmission could be inferred with phyloscanner in 24 pairs. In 5 pairs, the (epidemiologically inferred) source case had the selected CD4 measurement more than 1 year after the (epidemiologically inferred) recipient. In these pairs, the substantially lower CD4 cell count in the (epidemiologically inferred) source case could have arisen over the difference in measurement times, and it was thus possible that infection could have occurred the other way round. The odds ratio for incorrect phylogenetic inference among pairs with very large negative differences in CD4 measurement dates versus those with larger differences was

$$(2/3)/(3/16) = 3.56,$$

which was again not statistically significant (Fisher exact test, p-value 0.27). However, the magnitude of the odds ratio suggests that it may have been more appropriate to consider pairs with CD4 measurement dates within 1 year of diagnosis as basis for defining the validation data set. Pairs labelled 17, 18, 36, 44, 50, 65, 90, 108 in Supplementary Figures 9–12 did not meet these more stringent selection criteria. The true direction of transmission in pairs 18, 90 could be consistent with phyloscanner inference.

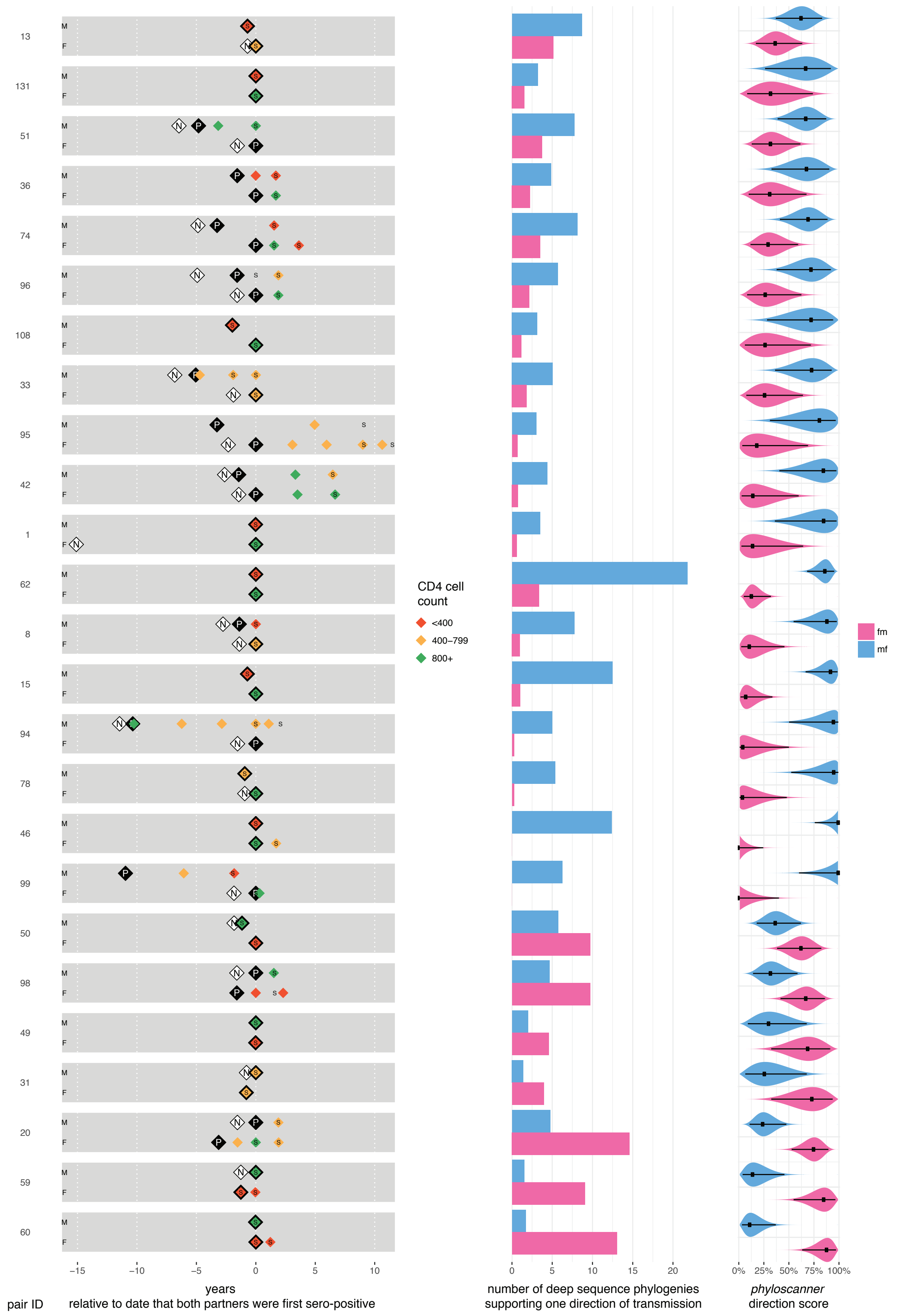

Supplementary Figure 9. Phylogenetically linked couples for whom the phylogenetically inferred direction of transmission was consistent with clinical data. Please see text for details.

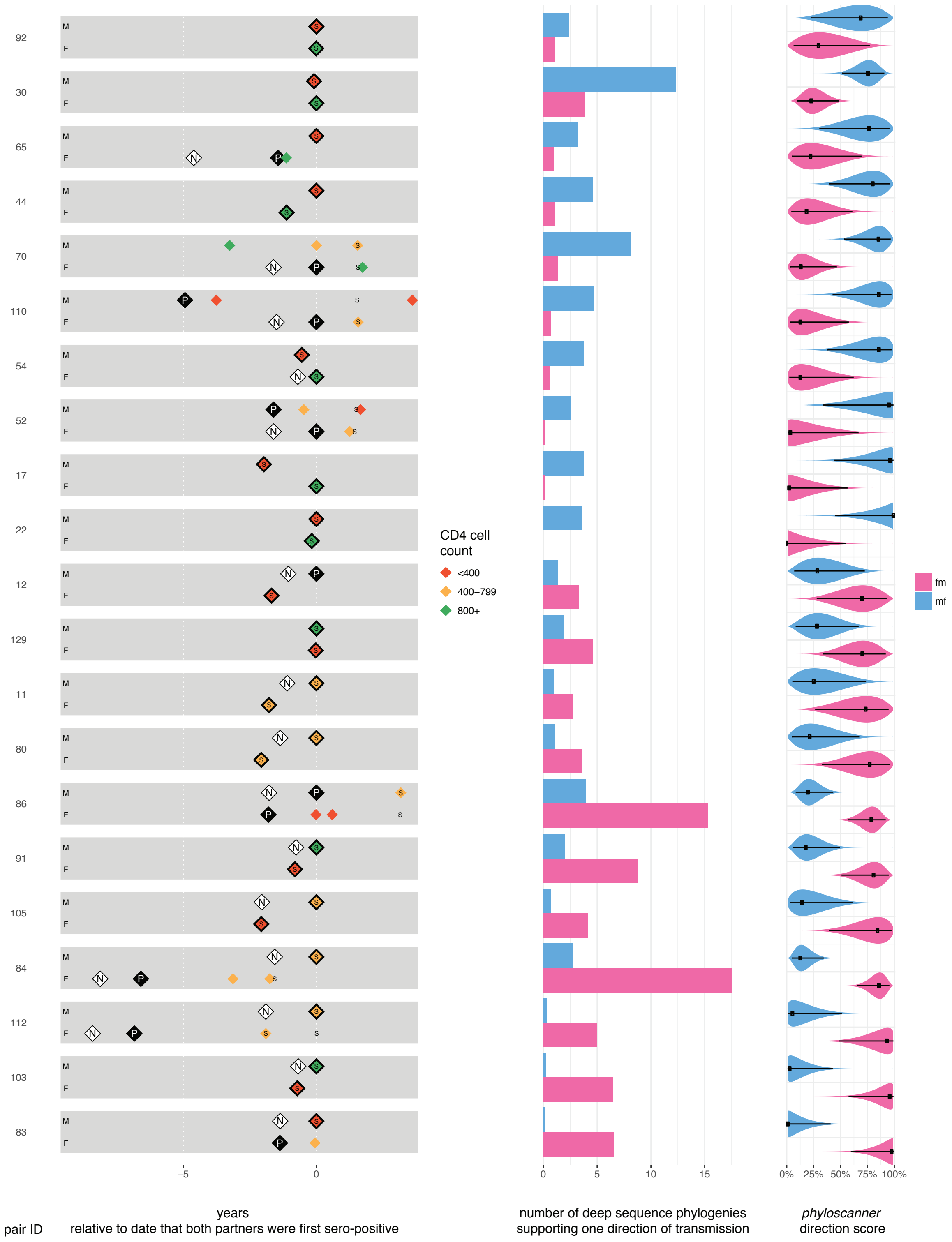

Supplementary Figure 10. Phylogenetically linked casual pairs for whom the phylogenetically inferred direction of transmission was consistent with clinical data. Please see text for details.

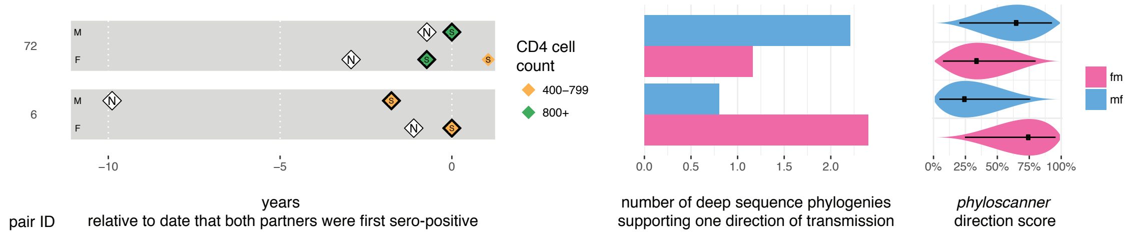

Supplementary Figure 11. Phylogenetically linked couples for whom the phylogenetically inferred direction of transmission was not consistent with clinical data. Please see text for details.

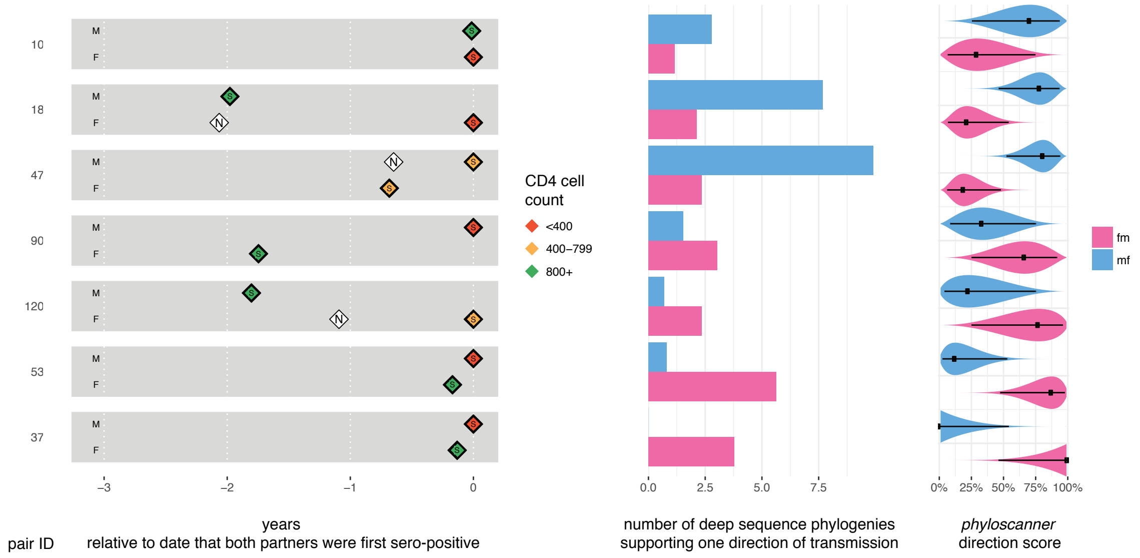

Supplementary Figure 12. Phylogenetically linked casual pairs for whom the phylogenetically inferred direction of transmission was not consistent with clinical data. Please see text for details.

Potential impact of sequence sampling times on phylogenetic inference into the direction of transmission.

Next, we examined wether particular patterns in sequence sampling times were associated with greater failure to correctly determine the direction of transmission. We hypothesized that true recipients who were sampled earlier might be more likely to appear as source in reconstructed deep-sequence phylogenies. The odds for incorrect phylogenetic inference of the source case were higher when the person, who was the recipient based on epidemiological data, was diagnosed first

$$(5/6)/(4/40) = 0.13,$$

and this was statistically significant (Fisher exact test, p-value 0.011). However, for the large majority individuals in the validation data set, sequencing was performed on the first positive sample (83 of 110). We therefore also considered the difference in times at which the blood sample for sequencing was taken. The odds for incorrect phylogenetic inference of the source case were again higher when the person, who was the recipient based on epidemiological data, was sequenced at an earlier date

$$(5/12)/(4/34) = 0.29,$$

though this was not statistically significant (Fisher exact test, p-value 0.116).

Potential shortcomings of the phyloscanner method on phylogenetic inference into the direction of transmission.

We further examined the deep-sequence phylogenies of the 9 phylogenetically linked pairs for whom the phylogenetically inferred direction of transmission was inconsistent with clinical data. In 10%-20% of those phylogenies, we found that reads from both partners which were essentially identical (subgraph distances below 10^{-6} substitutions per site) and basal in the corresponding subgraphs of both individuals (Supplementary Figure 13). In these cases, inferred ancestry should be in either direction with equal probability. However, due to consistently higher copy number of those reads in one individual, preference was systematically given for ancestral subgraph topologies in one of the two possible directions. This is likely a technical limitation that affected our inferences.

Summary

Supplementary Table 10 summarizes our investigations, indicating that potential reasons for why phylogenetic inference into the directon of transmission was inconsistent with clinical data could be isolated in 8/9 pairs.

Supplementary Table 10. Potential reasons on failure to infer direction of transmission from deep-sequence data.

Pair identifier	Known to have long-term sexual contact	Weak clinical indicator of direction of transmission	Epidemiologically identified recipient sampled before source	Technical limitations in inferring ancestry	Further comments
6	Yes	No	No	Yes	--
10	No	No	yes, a few days	No	No explanation on inconsistent phylogenetic inference
18	No	Yes	yes, 2 years	No	--
37	No	No	yes, two months		Deep sequencing relatively poor compared to most other samples
47	No	No	No	Yes	--
53	No	No	yes, two months	Yes	--
72	Yes	No	No	Yes	--
90	No	Yes	yes, two years	No	--
120	No	No	No	Yes	--

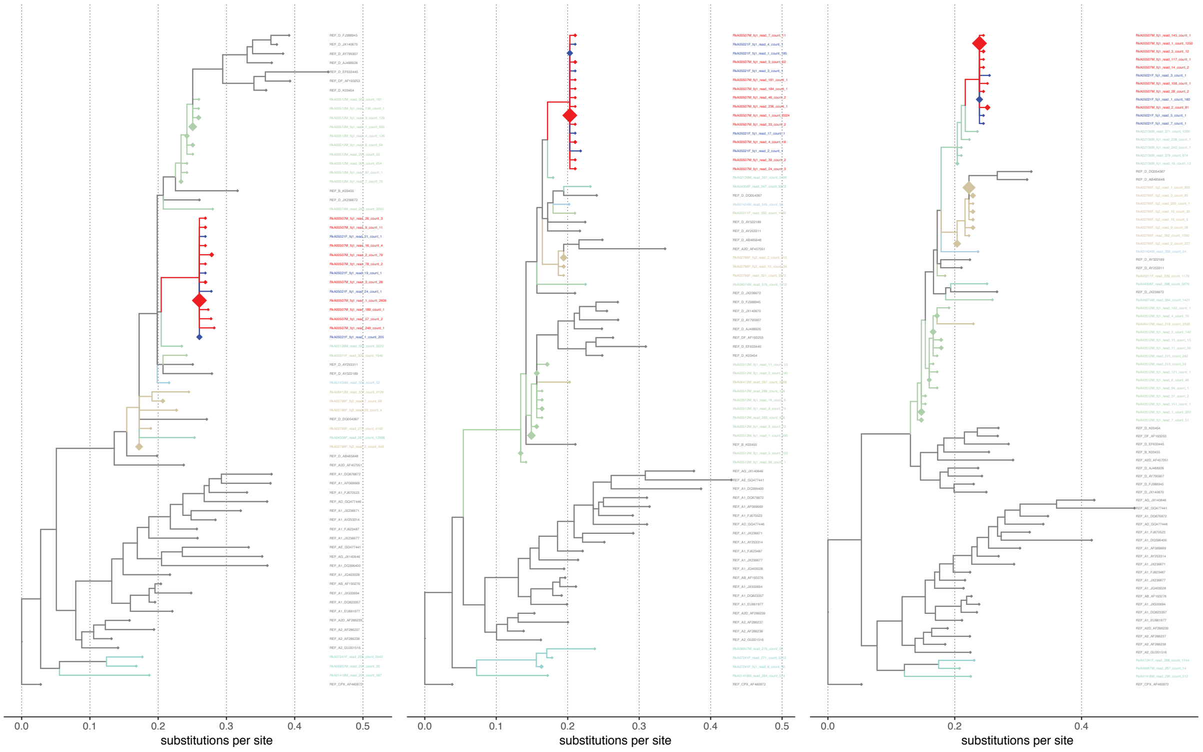

Supplementary Figure 13. Limitations in inferring ancestry between subgraphs with the phyloscanner method. Three consecutive deep-sequence phylogenies are shown, with subgraphs from the male partner (red) and female partner (blue) highlighted. Reads from both partners were basal in the corresponding subgraphs and essentially identical, suggesting that ancestry between the two individuals cannot be established in these phylogenies.

Supplementary References

1. Darriba, D., Taboada, G.L., Doallo, R. & Posada, D. jModelTest 2: more models, new heuristics and parallel computing. *Nat Methods* **9**, 772 (2012).
2. Kuiken, C. *et al.* HIV Sequence Compendium 2012. (ed. Theoretical Biology and Biophysics Group, L.A.N.L.) (NM, LA-UR 12-24653, 2012).
3. Stamatakis, A. RAxML version 8: a tool for phylogenetic analysis and post-analysis of large phylogenies. *Bioinformatics* **30**, 1312-3 (2014).
4. Gall, A. *et al.* Universal amplification, next-generation sequencing, and assembly of HIV-1 genomes. *J Clin Microbiol* **50**, 3838-44 (2012).
5. Wymant, C. *et al.* Easy and Accurate Reconstruction of Whole HIV Genomes from Short-Read Sequence Data. *bioRxiv* (2016).
6. Ratmann, O. *et al.* HIV-1 full-genome phylogenetics of generalized epidemics in sub-Saharan Africa: impact of missing nucleotide characters in next-generation sequences. *AIDS Res Hum Retroviruses* (2017).
7. Yang, Z. & Rannala, B. Molecular phylogenetics: principles and practice. *Nat Rev Genet* **13**, 303-14 (2012).
8. Wymant, C. *et al.* PHYLOSCANNER: Inferring Transmission from Within- and Between-Host Pathogen Genetic Diversity. *Mol Biol Evol* (2017).
9. Tamura, K. & Nei, M. Estimation of the number of nucleotide substitutions in the control region of mitochondrial DNA in humans and chimpanzees. *Mol Biol Evol* **10**, 512-26 (1993).
10. Paradis, E., Claude, J. & Strimmer, K. APE: Analyses of Phylogenetics and Evolution in R language. *Bioinformatics* **20**, 289-90 (2004).
11. Chan, S.K. *et al.* Likely female-to-female sexual transmission of HIV--Texas, 2012. *MMWR Morb Mortal Wkly Rep* **63**, 209-12 (2014).
