# Supplementary material for: Inferring HIV-1 transmission networks and sources of epidemic spread in Africa with deep-sequence phylogenetic analysis
Source: Nat Commun. 2019 Mar 29;10:1411. doi: 10.1038/s41467-019-09139-4 (PMC6441045; doi:10.1038/s41467-019-09139-4)
Supplement: Supplementary file 2 — Description of Additional Supplementary Files [file 41467_2019_9139_MOESM2_ESM.pdf]

Description of Additional Supplemenary Files

File: Supplementary Data 1

Descrition: Deep-sequence phylogenies of female-female pairs with near identical and intermingled subgraphs.

File: Supplementary Data 2

Descrition: Deep-sequence phylogenies of the nine male-female pairs for whom the phylogenetically inferred direction of transmission was inconsistent with clinical data.
