# Supplementary material for: Inferring HIV-1 transmission networks and sources of epidemic spread in Africa with deep-sequence phylogenetic analysis
Source: Nat Commun. 2019 Mar 29;10:1411. doi: 10.1038/s41467-019-09139-4 (PMC6441045; doi:10.1038/s41467-019-09139-4)
Supplement: Supplementary file 3 — Supplementary Data 1 [file 41467_2019_9139_MOESM3_ESM.pdf]

Deep sequence phylogenies
from two females RkA04565F, RkA05315F (run 38)
with near identical and intermingled subgraphs.

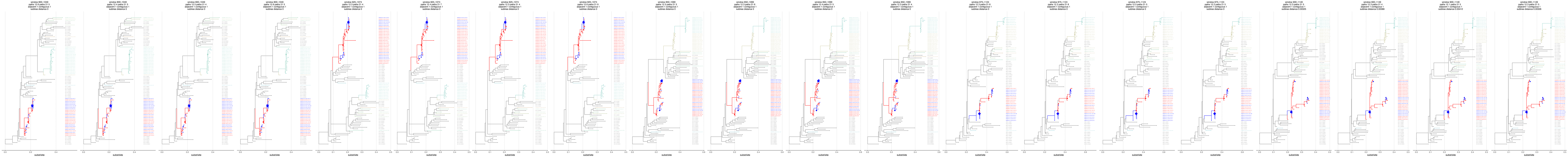
window 800–1049
paths 12 6 paths 21 5
adjacent 1 contiguous 1
subtree distance 0
window 800–1049
paths 12 4 paths 21 3
adjacent 1 contiguous 1
subtree distance 0
window 800–1049
paths 12 5 paths 21 4
adjacent 1 contiguous 1
subtree distance 0
window 800–1049
paths 12 6 paths 21 5
adjacent 1 contiguous 1
subtree distance 0
window 825–1074
paths 12 3 paths 21 4
adjacent 1 contiguous 1
subtree distance 0
window 825–1074
paths 12 4 paths 21 7
adjacent 1 contiguous 1
subtree distance 0
window 825–1074
paths 12 3 paths 21 4
adjacent 1 contiguous 1
subtree distance 0
window 825–1074
paths 12 6 paths 21 9
adjacent 1 contiguous 1
subtree distance 0
window 850–1099
paths 12 5 paths 21 3
adjacent 1 contiguous 1
subtree distance 0
window 850–1099
paths 12 3 paths 21 6
adjacent 1 contiguous 1
subtree distance 0
window 850–1099
paths 12 4 paths 21 5
adjacent 1 contiguous 1
subtree distance 0
window 850–1099
paths 12 3 paths 21 4
adjacent 1 contiguous 1
subtree distance 0
window 875–1124
paths 12 3 paths 21 8
adjacent 1 contiguous 1
subtree distance 0
window 875–1124
paths 12 2 paths 21 8
adjacent 1 contiguous 1
subtree distance 0
window 875–1124
paths 12 5 paths 21 5
adjacent 1 contiguous 1
subtree distance 0
window 875–1124
paths 12 2 paths 21 5
adjacent 1 contiguous 1
subtree distance 0
window 900–1149
paths 12 3 paths 21 5
adjacent 1 contiguous 1
subtree distance 0.00325
window 900–1149
paths 12 3 paths 21 4
adjacent 1 contiguous 1
subtree distance 0.00389
window 900–1149
paths 12 1 paths 21 5
adjacent 1 contiguous 1
subtree distance 0.00412
window 900–1149
paths 12 3 paths 21 5
adjacent 1 contiguous 1
subtree distance 0.00326

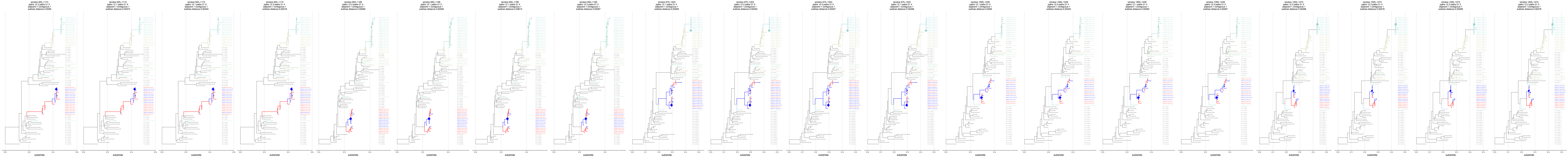
window 925–1174
paths 12 2 paths 21 3
adjacent 1 contiguous 1
subtree distance 0.0036
window 925–1174
paths 12 1 paths 21 3
adjacent 1 contiguous 1
subtree distance 0.00374
window 925–1174
paths 12 1 paths 21 3
adjacent 1 contiguous 1
subtree distance 0.00345
window 925–1174
paths 12 2 paths 21 4
adjacent 1 contiguous 1
subtree distance 0.00318
window 950–1199
paths 12 2 paths 21 2
adjacent 1 contiguous 1
subtree distance 0.00269
window 950–1199
paths 12 1 paths 21 2
adjacent 1 contiguous 1
subtree distance 0.00358
window 950–1199
paths 12 1 paths 21 4
adjacent 1 contiguous 1
subtree distance 0.00613
window 950–1199
paths 12 2 paths 21 3
adjacent 1 contiguous 1
subtree distance 0.00287
window 975–1224
paths 12 1 paths 21 4
adjacent 1 contiguous 1
subtree distance 0.00263
window 975–1224
paths 12 2 paths 21 2
adjacent 1 contiguous 1
subtree distance 0.00333
window 975–1224
paths 12 0 paths 21 5
adjacent 1 contiguous 1
subtree distance 0.00327
window 975–1224
paths 12 1 paths 21 4
adjacent 1 contiguous 1
subtree distance 0.00269
window 1000–1249
paths 12 1 paths 21 5
adjacent 1 contiguous 1
subtree distance 0.0056
window 1000–1249
paths 12 0 paths 21 4
adjacent 1 contiguous 1
subtree distance 0.00342
window 1000–1249
paths 12 1 paths 21 4
adjacent 1 contiguous 1
subtree distance 0.00262
window 1000–1249
paths 12 0 paths 21 5
adjacent 1 contiguous 1
subtree distance 0.00287
window 1025–1274
paths 12 2 paths 21 2
adjacent 1 contiguous 1
subtree distance 0.00268
window 1025–1274
paths 12 3 paths 21 4
adjacent 1 contiguous 1
subtree distance 0.00279
window 1025–1274
paths 12 2 paths 21 2
adjacent 1 contiguous 1
subtree distance 0.00299
window 1025–1274
paths 12 2 paths 21 2
adjacent 1 contiguous 1
subtree distance 0.00279
subst/site

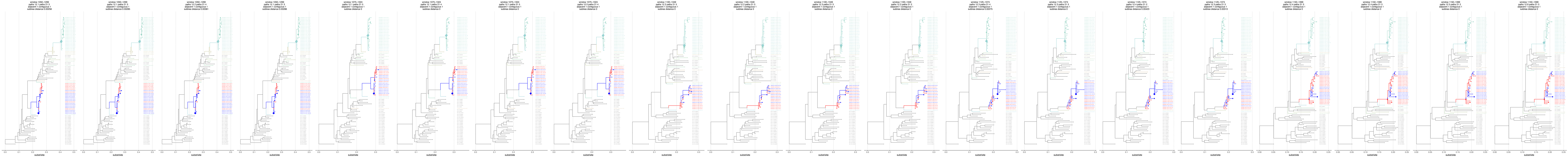
window 1050–1299
paths 12 1 paths 21 3
adjacent 1 contiguous 1
subtree distance 0.00258
window 1050–1299
paths 12 1 paths 21 3
adjacent 1 contiguous 1
subtree distance 0.00285
window 1050–1299
paths 12 2 paths 21 4
adjacent 1 contiguous 1
subtree distance 0.00381
window 1050–1299
paths 12 1 paths 21 3
adjacent 1 contiguous 1
subtree distance 0.00286
window 1075–1324
paths 12 1 paths 21 5
adjacent 1 contiguous 1
subtree distance 0
window 1075–1324
paths 12 1 paths 21 4
adjacent 1 contiguous 1
subtree distance 0
window 1075–1324
paths 12 1 paths 21 5
adjacent 1 contiguous 1
subtree distance 0
window 1075–1324
paths 12 0 paths 21 4
adjacent 1 contiguous 1
subtree distance 0
window 1100–1349
paths 12 2 paths 21 5
adjacent 1 contiguous 1
subtree distance 0
window 1100–1349
paths 12 2 paths 21 3
adjacent 1 contiguous 1
subtree distance 0
window 1100–1349
paths 12 3 paths 21 3
adjacent 1 contiguous 1
subtree distance 0
window 1100–1349
paths 12 2 paths 21 2
adjacent 1 contiguous 1
subtree distance 0
window 1125–1374
paths 12 3 paths 21 4
adjacent 1 contiguous 1
subtree distance 0.00275
window 1125–1374
paths 12 3 paths 21 3
adjacent 1 contiguous 1
subtree distance 0
window 1125–1374
paths 12 4 paths 21 2
adjacent 1 contiguous 1
subtree distance 0.00423
window 1125–1374
paths 12 3 paths 21 3
adjacent 1 contiguous 1
subtree distance 0.00313
window 1150–1399
paths 12 4 paths 21 3
adjacent 1 contiguous 1
subtree distance 0
window 1150–1399
paths 12 4 paths 21 3
adjacent 1 contiguous 1
subtree distance 0
window 1150–1399
paths 12 5 paths 21 2
adjacent 1 contiguous 1
subtree distance 0
window 1150–1399
paths 12 6 paths 21 3
adjacent 1 contiguous 1
subtree distance 0
subst/site

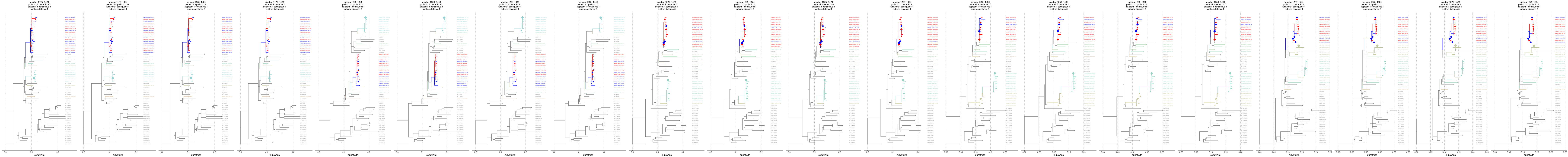

window 1175–1424
paths 12 2 paths 21 10
adjacent 1 contiguous 1
subtree distance 0
window 1175–1424
paths 12 4 paths 21 10
adjacent 1 contiguous 1
subtree distance 0
window 1175–1424
paths 12 3 paths 21 9
adjacent 1 contiguous 1
subtree distance 0
window 1175–1424
paths 12 2 paths 21 7
adjacent 1 contiguous 1
subtree distance 0
window 1200–1449
paths 12 2 paths 21 4
adjacent 1 contiguous 1
subtree distance 0
window 1200–1449
paths 12 2 paths 21 10
adjacent 1 contiguous 1
subtree distance 0
window 1200–1449
paths 12 2 paths 21 7
adjacent 1 contiguous 1
subtree distance 0
window 1200–1449
paths 12 1 paths 21 7
adjacent 1 contiguous 1
subtree distance 0
window 1225–1474
paths 12 2 paths 21 7
adjacent 1 contiguous 1
subtree distance 0
window 1225–1474
paths 12 3 paths 21 6
adjacent 1 contiguous 1
subtree distance 0
window 1225–1474
paths 12 1 paths 21 8
adjacent 1 contiguous 1
subtree distance 0
window 1225–1474
paths 12 1 paths 21 7
adjacent 1 contiguous 1
subtree distance 0
window 1250–1499
paths 12 1 paths 21 10
adjacent 1 contiguous 1
subtree distance 0
window 1250–1499
paths 12 3 paths 21 7
adjacent 1 contiguous 1
subtree distance 0
window 1250–1499
paths 12 1 paths 21 8
adjacent 1 contiguous 1
subtree distance 0
window 1250–1499
paths 12 1 paths 21 7
adjacent 1 contiguous 1
subtree distance 0
window 1275–1524
paths 12 1 paths 21 4
adjacent 1 contiguous 1
subtree distance 0
window 1275–1524
paths 12 2 paths 21 2
adjacent 1 contiguous 1
subtree distance 0
window 1275–1524
paths 12 3 paths 21 2
adjacent 1 contiguous 1
subtree distance 0
window 1275–1524
paths 12 1 paths 21 2
adjacent 1 contiguous 1
subtree distance 0
subst/site

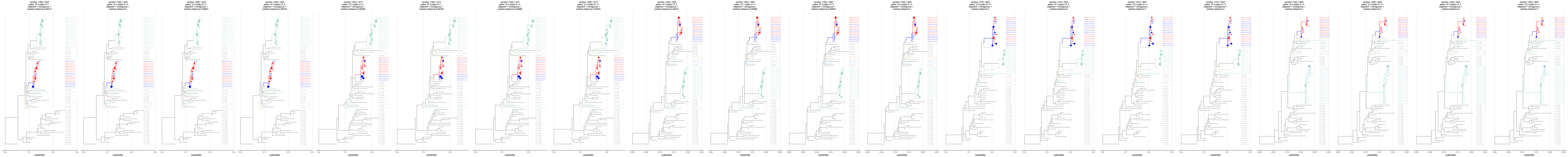
window 1300–1549
paths 12 3 paths 21 1
adjacent 1 contiguous 1
subtree distance 0.00514
window 1300–1549
paths 12 4 paths 21 3
adjacent 1 contiguous 1
subtree distance 0
window 1300–1549
paths 12 2 paths 21 2
adjacent 1 contiguous 1
subtree distance 0.00661
window 1300–1549
paths 12 2 paths 21 2
adjacent 1 contiguous 1
subtree distance 0.00479
window 1325–1574
paths 12 5 paths 21 0
adjacent 1 contiguous 1
subtree distance 0.00585
window 1325–1574
paths 12 2 paths 21 1
adjacent 1 contiguous 1
subtree distance 0.00763
window 1325–1574
paths 12 2 paths 21 2
adjacent 1 contiguous 1
subtree distance 0.00886
window 1325–1574
paths 12 2 paths 21 2
adjacent 1 contiguous 1
subtree distance 0.00555
window 1350–1599
paths 12 2 paths 21 2
adjacent 1 contiguous 1
subtree distance 0.00547
window 1350–1599
paths 12 1 paths 21 4
adjacent 1 contiguous 1
subtree distance 0.00892
window 1350–1599
paths 12 2 paths 21 3
adjacent 1 contiguous 1
subtree distance 0.00652
window 1350–1599
paths 12 3 paths 21 2
adjacent 1 contiguous 1
subtree distance 0
window 1375–1624
paths 12 3 paths 21 3
adjacent 1 contiguous 1
subtree distance 0
window 1375–1624
paths 12 2 paths 21 3
adjacent 1 contiguous 1
subtree distance 0
window 1375–1624
paths 12 2 paths 21 4
adjacent 1 contiguous 1
subtree distance 0
window 1375–1624
paths 12 4 paths 21 3
adjacent 1 contiguous 1
subtree distance 0
window 1400–1649
paths 12 2 paths 21 3
adjacent 1 contiguous 1
subtree distance 0
window 1400–1649
paths 12 2 paths 21 3
adjacent 1 contiguous 1
subtree distance 0
window 1400–1649
paths 12 2 paths 21 3
adjacent 1 contiguous 1
subtree distance 0
window 1400–1649
paths 12 2 paths 21 4
adjacent 1 contiguous 1
subtree distance 0

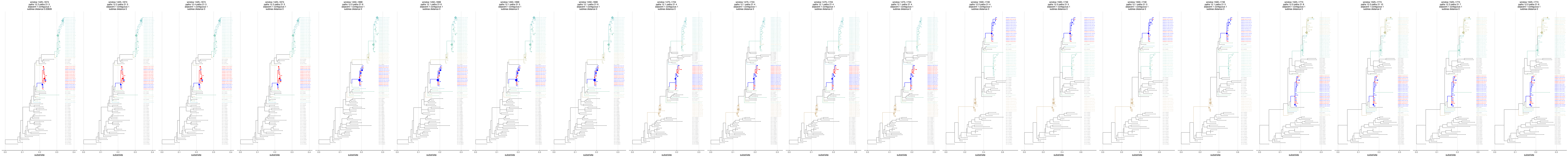

window 1425–1674
paths 12 2 paths 21 3
adjacent 1 contiguous 1
subtree distance 0.00828
window 1425–1674
paths 12 2 paths 21 3
adjacent 1 contiguous 1
subtree distance 0
window 1425–1674
paths 12 4 paths 21 3
adjacent 1 contiguous 1
subtree distance 0
window 1425–1674
paths 12 2 paths 21 3
adjacent 1 contiguous 1
subtree distance 0
window 1450–1699
paths 12 0 paths 21 6
adjacent 1 contiguous 1
subtree distance 0
window 1450–1699
paths 12 1 paths 21 6
adjacent 1 contiguous 1
subtree distance 0
window 1450–1699
paths 12 1 paths 21 5
adjacent 1 contiguous 1
subtree distance 0
window 1450–1699
paths 12 1 paths 21 6
adjacent 1 contiguous 1
subtree distance 0
window 1475–1724
paths 12 1 paths 21 4
adjacent 1 contiguous 1
subtree distance 0
window 1475–1724
paths 12 1 paths 21 4
adjacent 1 contiguous 1
subtree distance 0
window 1475–1724
paths 12 1 paths 21 4
adjacent 1 contiguous 1
subtree distance 0
window 1475–1724
paths 12 1 paths 21 4
adjacent 1 contiguous 1
subtree distance 0
window 1500–1749
paths 12 0 paths 21 4
adjacent 1 contiguous 1
subtree distance 0
window 1500–1749
paths 12 0 paths 21 5
adjacent 1 contiguous 1
subtree distance 0
window 1500–1749
paths 12 1 paths 21 3
adjacent 1 contiguous 1
subtree distance 0
window 1500–1749
paths 12 1 paths 21 5
adjacent 1 contiguous 1
subtree distance 0
window 1525–1774
paths 12 0 paths 21 8
adjacent 1 contiguous 1
subtree distance 0
window 1525–1774
paths 12 0 paths 21 10
adjacent 1 contiguous 1
subtree distance 0
window 1525–1774
paths 12 2 paths 21 7
adjacent 1 contiguous 1
subtree distance 0
window 1525–1774
paths 12 0 paths 21 6
adjacent 1 contiguous 1
subtree distance 0

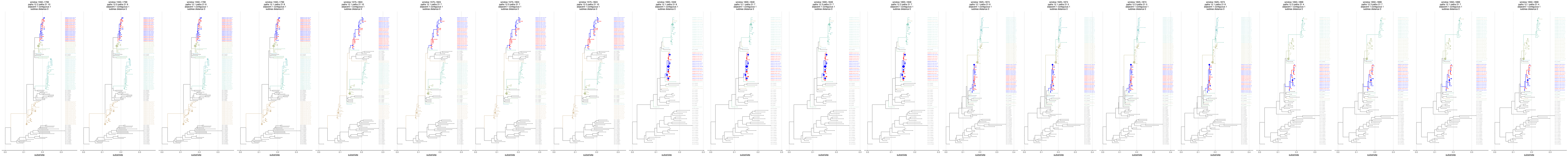
window 1550–1799
paths 12 2 paths 21 10
adjacent 1 contiguous 1
subtree distance 0
window 1550–1799
paths 12 0 paths 21 9
adjacent 1 contiguous 1
subtree distance 0
window 1550–1799
paths 12 1 paths 21 8
adjacent 1 contiguous 1
subtree distance 0
window 1550–1799
paths 12 1 paths 21 8
adjacent 1 contiguous 1
subtree distance 0
window 1575–1824
paths 12 1 paths 21 10
adjacent 1 contiguous 1
subtree distance 0
window 1575–1824
paths 12 1 paths 21 7
adjacent 1 contiguous 1
subtree distance 0
window 1575–1824
paths 12 0 paths 21 7
adjacent 1 contiguous 1
subtree distance 0
window 1575–1824
paths 12 0 paths 21 10
adjacent 1 contiguous 1
subtree distance 0
window 1600–1849
paths 12 1 paths 21 8
adjacent 1 contiguous 1
subtree distance 0
window 1600–1849
paths 12 1 paths 21 7
adjacent 1 contiguous 1
subtree distance 0
window 1600–1849
paths 12 3 paths 21 7
adjacent 1 contiguous 1
subtree distance 0
window 1600–1849
paths 12 2 paths 21 7
adjacent 1 contiguous 1
subtree distance 0
window 1625–1874
paths 12 1 paths 21 6
adjacent 1 contiguous 1
subtree distance 0
window 1625–1874
paths 12 1 paths 21 5
adjacent 1 contiguous 1
subtree distance 0
window 1625–1874
paths 12 3 paths 21 5
adjacent 1 contiguous 1
subtree distance 0
window 1625–1874
paths 12 1 paths 21 8
adjacent 1 contiguous 1
subtree distance 0
window 1650–1899
paths 12 3 paths 21 4
adjacent 1 contiguous 1
subtree distance 0
window 1650–1899
paths 12 3 paths 21 7
adjacent 1 contiguous 1
subtree distance 0
window 1650–1899
paths 12 1 paths 21 7
adjacent 1 contiguous 1
subtree distance 0
window 1650–1899
paths 12 1 paths 21 4
adjacent 1 contiguous 1
subtree distance 0
subst/site

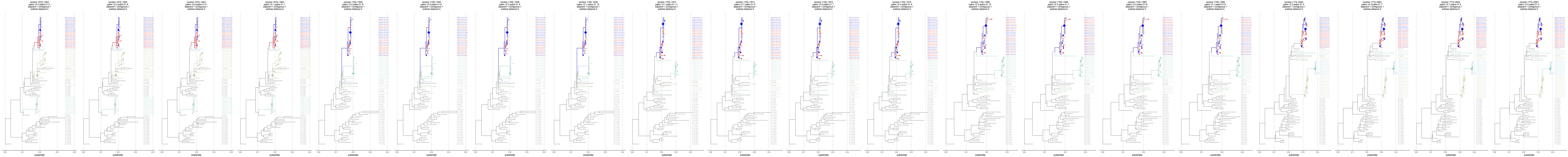
window 1675–1924
paths 12 4 paths 21 2
adjacent 1 contiguous 1
subtree distance 0
subst/site
window 1675–1924
paths 12 2 paths 21 9
adjacent 1 contiguous 1
subtree distance 0
subst/site
window 1675–1924
paths 12 6 paths 21 6
adjacent 1 contiguous 1
subtree distance 0
subst/site
window 1675–1924
paths 12 1 paths 21 7
adjacent 1 contiguous 1
subtree distance 0
subst/site
window 1700–1949
paths 12 2 paths 21 8
adjacent 1 contiguous 1
subtree distance 0
subst/site
window 1700–1949
paths 12 3 paths 21 8
adjacent 1 contiguous 1
subtree distance 0
subst/site
window 1700–1949
paths 12 2 paths 21 9
adjacent 1 contiguous 1
subtree distance 0
subst/site
window 1700–1949
paths 12 1 paths 21 13
adjacent 1 contiguous 1
subtree distance 0
subst/site
window 1725–1974
paths 12 1 paths 21 11
adjacent 1 contiguous 1
subtree distance 0
subst/site
window 1725–1974
paths 12 1 paths 21 9
adjacent 1 contiguous 1
subtree distance 0
subst/site
window 1725–1974
paths 12 3 paths 21 7
adjacent 1 contiguous 1
subtree distance 0
subst/site
window 1725–1974
paths 12 4 paths 21 6
adjacent 1 contiguous 1
subtree distance 0
subst/site
window 1750–1999
paths 12 0 paths 21 12
adjacent 1 contiguous 1
subtree distance 0
subst/site
window 1750–1999
paths 12 4 paths 21 7
adjacent 1 contiguous 1
subtree distance 0
subst/site
window 1750–1999
paths 12 3 paths 21 8
adjacent 1 contiguous 1
subtree distance 0
subst/site
window 1750–1999
paths 12 1 paths 21 9
adjacent 1 contiguous 1
subtree distance 0
subst/site
window 1775–2024
paths 12 3 paths 21 5
adjacent 1 contiguous 1
subtree distance 0
subst/site
window 1775–2024
paths 12 3 paths 21 7
adjacent 1 contiguous 1
subtree distance 0
subst/site
window 1775–2024
paths 12 1 paths 21 9
adjacent 1 contiguous 1
subtree distance 0
subst/site
window 1775–2024
paths 12 5 paths 21 4
adjacent 1 contiguous 1
subtree distance 0
subst/site

Deep sequence phylogenies
from two females RkA01255F, RkA06631F (run 275)
with near identical and intermingled subgraphs.

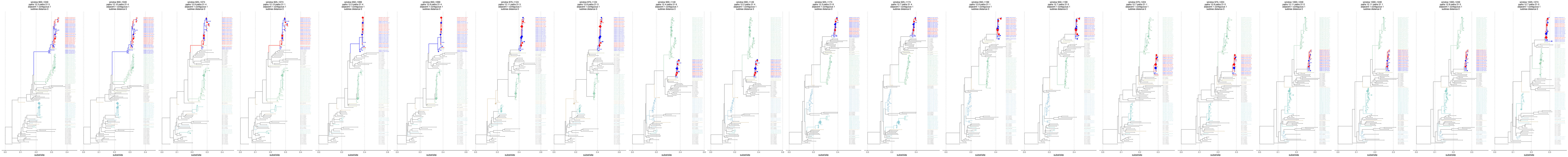
window 800–1049
paths 12 5 paths 21 5
adjacent 1 contiguous 1
subtree distance 0
window 800–1049
paths 12 10 paths 21 4
adjacent 1 contiguous 1
subtree distance 0
window 825–1074
paths 12 9 paths 21 4
adjacent 1 contiguous 1
subtree distance 0
window 825–1074
paths 12 13 paths 21 1
adjacent 1 contiguous 1
subtree distance 0
window 850–1099
paths 12 3 paths 21 4
adjacent 1 contiguous 1
subtree distance 0
window 850–1099
paths 12 8 paths 21 4
adjacent 1 contiguous 1
subtree distance 0
window 875–1124
paths 12 11 paths 21 5
adjacent 1 contiguous 1
subtree distance 0
window 875–1124
paths 12 8 paths 21 3
adjacent 1 contiguous 1
subtree distance 0
window 900–1149
paths 12 4 paths 21 6
adjacent 1 contiguous 1
subtree distance 0
window 900–1149
paths 12 2 paths 21 4
adjacent 1 contiguous 1
subtree distance 0
window 925–1174
paths 12 3 paths 21 8
adjacent 1 contiguous 1
subtree distance 0
window 925–1174
paths 12 7 paths 21 4
adjacent 1 contiguous 1
subtree distance 0
window 950–1199
paths 12 8 paths 21 1
adjacent 1 contiguous 1
subtree distance 0
window 950–1199
paths 12 7 paths 21 0
adjacent 1 contiguous 1
subtree distance 0
window 975–1224
paths 12 7 paths 21 1
adjacent 1 contiguous 1
subtree distance 0
window 975–1224
paths 12 8 paths 21 0
adjacent 1 contiguous 1
subtree distance 0
window 1000–1249
paths 12 11 paths 21 0
adjacent 1 contiguous 1
subtree distance 0
window 1000–1249
paths 12 11 paths 21 1
adjacent 1 contiguous 1
subtree distance 0
window 1000–1249
paths 12 8 paths 21 0
adjacent 1 contiguous 1
subtree distance 0
window 1025–1274
paths 12 7 paths 21 0
adjacent 1 contiguous 1
subtree distance 0

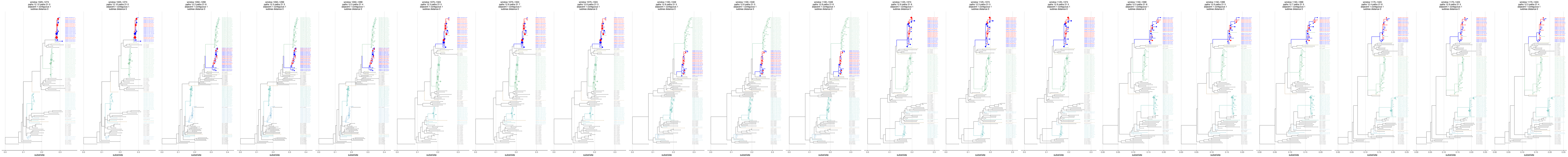
window 1025–1274
paths 12 12 paths 21 0
adjacent 1 contiguous 1
subtree distance 0
window 1025–1274
paths 12 10 paths 21 0
adjacent 1 contiguous 1
subtree distance 0
window 1050–1299
paths 12 2 paths 21 8
adjacent 1 contiguous 1
subtree distance 0
window 1050–1299
paths 12 5 paths 21 3
adjacent 1 contiguous 1
subtree distance 0
window 1050–1299
paths 12 5 paths 21 4
adjacent 1 contiguous 1
subtree distance 0
window 1075–1324
paths 12 5 paths 21 5
adjacent 1 contiguous 1
subtree distance 0
window 1075–1324
paths 12 6 paths 21 7
adjacent 1 contiguous 1
subtree distance 0
window 1075–1324
paths 12 6 paths 21 3
adjacent 1 contiguous 1
subtree distance 0
window 1100–1349
paths 12 6 paths 21 5
adjacent 1 contiguous 1
subtree distance 0
window 1100–1349
paths 12 6 paths 21 5
adjacent 1 contiguous 1
subtree distance 0
window 1100–1349
paths 12 8 paths 21 6
adjacent 1 contiguous 1
subtree distance 0
window 1125–1374
paths 12 6 paths 21 6
adjacent 1 contiguous 1
subtree distance 0
window 1125–1374
paths 12 5 paths 21 5
adjacent 1 contiguous 1
subtree distance 0
window 1125–1374
paths 12 6 paths 21 5
adjacent 1 contiguous 1
subtree distance 0
window 1150–1399
paths 12 5 paths 21 8
adjacent 1 contiguous 1
subtree distance 0
window 1150–1399
paths 12 6 paths 21 5
adjacent 1 contiguous 1
subtree distance 0
window 1150–1399
paths 12 7 paths 21 5
adjacent 1 contiguous 1
subtree distance 0
window 1175–1424
paths 12 4 paths 21 8
adjacent 1 contiguous 1
subtree distance 0
window 1175–1424
paths 12 6 paths 21 5
adjacent 1 contiguous 1
subtree distance 0
window 1175–1424
paths 12 5 paths 21 4
adjacent 1 contiguous 1
subtree distance 0
subst/site

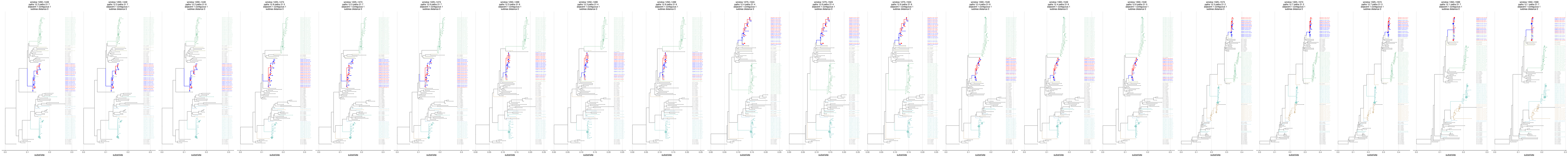

window 1200–1449
paths 12 2 paths 21 7
adjacent 1 contiguous 1
subtree distance 0
subst/site
window 1200–1449
paths 12 3 paths 21 7
adjacent 1 contiguous 1
subtree distance 0
subst/site
window 1200–1449
paths 12 2 paths 21 8
adjacent 1 contiguous 1
subtree distance 0
subst/site
window 1225–1474
paths 12 8 paths 21 5
adjacent 1 contiguous 1
subtree distance 0
subst/site
window 1225–1474
paths 12 5 paths 21 7
adjacent 1 contiguous 1
subtree distance 0
subst/site
window 1225–1474
paths 12 6 paths 21 7
adjacent 1 contiguous 1
subtree distance 0
subst/site
window 1250–1499
paths 12 3 paths 21 6
adjacent 1 contiguous 1
subtree distance 0
subst/site
window 1250–1499
paths 12 3 paths 21 4
adjacent 1 contiguous 1
subtree distance 0
subst/site
window 1250–1499
paths 12 6 paths 21 6
adjacent 1 contiguous 1
subtree distance 0
subst/site
window 1275–1524
paths 12 4 paths 21 4
adjacent 1 contiguous 1
subtree distance 0
subst/site
window 1275–1524
paths 12 6 paths 21 4
adjacent 1 contiguous 1
subtree distance 0
subst/site
window 1275–1524
paths 12 6 paths 21 6
adjacent 1 contiguous 1
subtree distance 0
subst/site
window 1300–1549
paths 12 4 paths 21 6
adjacent 1 contiguous 1
subtree distance 0
subst/site
window 1300–1549
paths 12 4 paths 21 6
adjacent 1 contiguous 1
subtree distance 0
subst/site
window 1300–1549
paths 12 6 paths 21 5
adjacent 1 contiguous 1
subtree distance 0
subst/site
window 1325–1574
paths 12 5 paths 21 2
adjacent 1 contiguous 1
subtree distance 0
subst/site
window 1325–1574
paths 12 7 paths 21 2
adjacent 1 contiguous 1
subtree distance 0
subst/site
window 1325–1574
paths 12 7 paths 21 3
adjacent 1 contiguous 1
subtree distance 0
subst/site
window 1350–1599
paths 12 1 paths 21 7
adjacent 1 contiguous 1
subtree distance 0
subst/site
window 1350–1599
paths 12 1 paths 21 7
adjacent 1 contiguous 1
subtree distance 0
subst/site

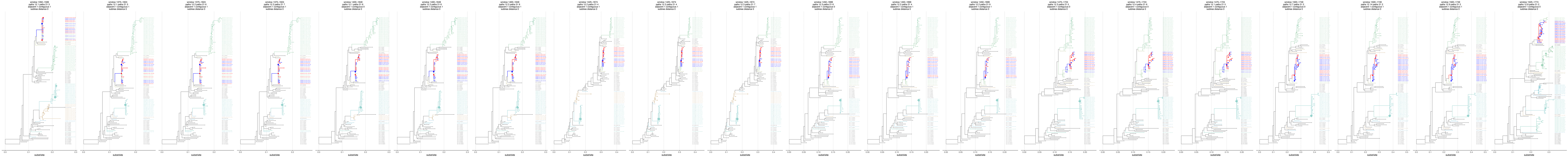
window 1350–1599
paths 12 1 paths 21 3
adjacent 1 contiguous 1
subtree distance 0
window 1375–1624
paths 12 1 paths 21 3
adjacent 1 contiguous 1
subtree distance 0
window 1375–1624
paths 12 2 paths 21 6
adjacent 1 contiguous 1
subtree distance 0
window 1375–1624
paths 12 2 paths 21 7
adjacent 1 contiguous 1
subtree distance 0
window 1400–1649
paths 12 1 paths 21 6
adjacent 1 contiguous 0
subtree distance 0
window 1400–1649
paths 12 2 paths 21 6
adjacent 1 contiguous 1
subtree distance 0
window 1400–1649
paths 12 2 paths 21 9
adjacent 1 contiguous 1
subtree distance 0
window 1425–1674
paths 12 2 paths 21 4
adjacent 1 contiguous 1
subtree distance 0
window 1425–1674
paths 12 2 paths 21 4
adjacent 1 contiguous 1
subtree distance 0
window 1425–1674
paths 12 3 paths 21 7
adjacent 1 contiguous 1
subtree distance 0
window 1450–1699
paths 12 3 paths 21 6
adjacent 1 contiguous 0
subtree distance 0
window 1450–1699
paths 12 2 paths 21 4
adjacent 1 contiguous 1
subtree distance 0
window 1450–1699
paths 12 2 paths 21 9
adjacent 1 contiguous 1
subtree distance 0
window 1475–1724
paths 12 3 paths 21 5
adjacent 1 contiguous 0
subtree distance 0
window 1475–1724
paths 12 4 paths 21 6
adjacent 1 contiguous 0
subtree distance 0
window 1475–1724
paths 12 1 paths 21 5
adjacent 1 contiguous 1
subtree distance 0
window 1500–1749
paths 12 7 paths 21 2
adjacent 1 contiguous 0
subtree distance 0
window 1500–1749
paths 12 14 paths 21 2
adjacent 1 contiguous 1
subtree distance 0
window 1500–1749
paths 12 9 paths 21 2
adjacent 1 contiguous 1
subtree distance 0
window 1525–1774
paths 12 8 paths 21 2
adjacent 1 contiguous 0
subtree distance 0
subst/site

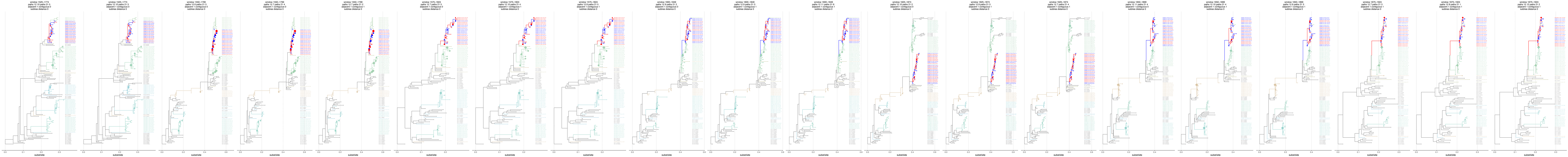
window 1525–1774
paths 12 10 paths 21 5
adjacent 1 contiguous 0
subtree distance 0
subst/site
window 1525–1774
paths 12 10 paths 21 3
adjacent 1 contiguous 0
subtree distance 0
subst/site
window 1550–1799
paths 12 6 paths 21 3
adjacent 1 contiguous 0
subtree distance 0
subst/site
window 1550–1799
paths 12 7 paths 21 4
adjacent 1 contiguous 0
subtree distance 0
subst/site
window 1550–1799
paths 12 7 paths 21 2
adjacent 1 contiguous 1
subtree distance 0
subst/site
window 1575–1824
paths 12 7 paths 21 3
adjacent 1 contiguous 0
subtree distance 0
subst/site
window 1575–1824
paths 12 10 paths 21 4
adjacent 1 contiguous 0
subtree distance 0
subst/site
window 1575–1824
paths 12 8 paths 21 5
adjacent 1 contiguous 1
subtree distance 0
subst/site
window 1600–1849
paths 12 9 paths 21 2
adjacent 1 contiguous 0
subtree distance 0
subst/site
window 1600–1849
paths 12 9 paths 21 3
adjacent 1 contiguous 1
subtree distance 0
subst/site
window 1600–1849
paths 12 11 paths 21 6
adjacent 1 contiguous 1
subtree distance 0
subst/site
window 1625–1874
paths 12 10 paths 21 2
adjacent 1 contiguous 0
subtree distance 0
subst/site
window 1625–1874
paths 12 8 paths 21 3
adjacent 1 contiguous 1
subtree distance 0
subst/site
window 1625–1874
paths 12 7 paths 21 4
adjacent 1 contiguous 1
subtree distance 0
subst/site
window 1650–1899
paths 12 11 paths 21 4
adjacent 1 contiguous 0
subtree distance 0
subst/site
window 1650–1899
paths 12 10 paths 21 4
adjacent 1 contiguous 1
subtree distance 0
subst/site
window 1650–1899
paths 12 6 paths 21 3
adjacent 1 contiguous 1
subtree distance 0
subst/site
window 1675–1924
paths 12 7 paths 21 3
adjacent 1 contiguous 1
subtree distance 0
subst/site
window 1675–1924
paths 12 8 paths 21 1
adjacent 1 contiguous 1
subtree distance 0
subst/site
window 1675–1924
paths 12 12 paths 21 2
adjacent 1 contiguous 1
subtree distance 0
subst/site

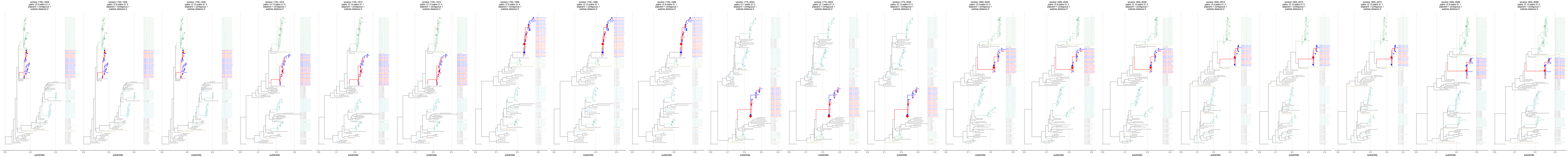
window 1700–1949
paths 12 6 paths 21 5
adjacent 1 contiguous 1
subtree distance 0
window 1700–1949
paths 12 8 paths 21 3
adjacent 1 contiguous 1
subtree distance 0
window 1700–1949
paths 12 10 paths 21 4
adjacent 1 contiguous 1
subtree distance 0
window 1725–1974
paths 12 13 paths 21 0
adjacent 1 contiguous 1
subtree distance 0
window 1725–1974
paths 12 14 paths 21 1
adjacent 1 contiguous 1
subtree distance 0
window 1725–1974
paths 12 13 paths 21 0
adjacent 1 contiguous 1
subtree distance 0
window 1750–1999
paths 12 9 paths 21 4
adjacent 1 contiguous 1
subtree distance 0
window 1750–1999
paths 12 10 paths 21 1
adjacent 1 contiguous 1
subtree distance 0
window 1750–1999
paths 12 9 paths 21 1
adjacent 1 contiguous 1
subtree distance 0
window 1775–2024
paths 12 7 paths 21 2
adjacent 1 contiguous 1
subtree distance 0
window 1775–2024
paths 12 7 paths 21 3
adjacent 1 contiguous 1
subtree distance 0
window 1775–2024
paths 12 12 paths 21 0
adjacent 1 contiguous 1
subtree distance 0
window 1800–2049
paths 12 6 paths 21 3
adjacent 1 contiguous 1
subtree distance 0
window 1800–2049
paths 12 8 paths 21 2
adjacent 1 contiguous 1
subtree distance 0
window 1800–2049
paths 12 10 paths 21 2
adjacent 1 contiguous 1
subtree distance 0
window 1825–2074
paths 12 9 paths 21 2
adjacent 1 contiguous 1
subtree distance 0
window 1825–2074
paths 12 10 paths 21 0
adjacent 1 contiguous 1
subtree distance 0
window 1825–2074
paths 12 13 paths 21 1
adjacent 1 contiguous 1
subtree distance 0
window 1850–2099
paths 12 9 paths 21 1
adjacent 1 contiguous 1
subtree distance 0
window 1850–2099
paths 12 13 paths 21 0
adjacent 1 contiguous 1
subtree distance 0
subst/site

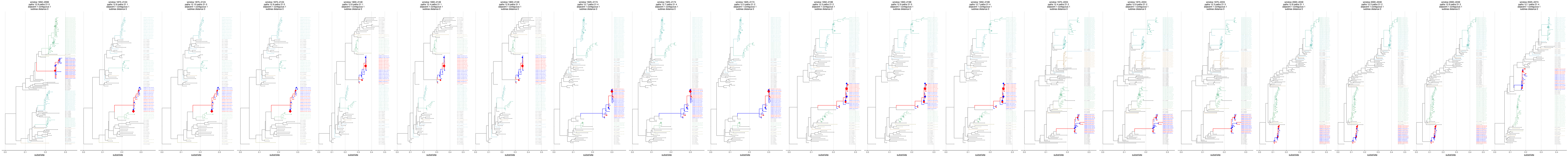
window 1850–2099
paths 12 8 paths 21 0
adjacent 1 contiguous 1
subtree distance 0
window 1875–2124
paths 12 8 paths 21 1
adjacent 1 contiguous 1
subtree distance 0
window 1875–2124
paths 12 10 paths 21 0
adjacent 1 contiguous 1
subtree distance 0
window 1875–2124
paths 12 9 paths 21 0
adjacent 1 contiguous 1
subtree distance 0
window 1900–2149
paths 12 8 paths 21 1
adjacent 1 contiguous 1
subtree distance 0
window 1900–2149
paths 12 4 paths 21 1
adjacent 1 contiguous 1
subtree distance 0
window 1900–2149
paths 12 8 paths 21 1
adjacent 1 contiguous 1
subtree distance 0
window 1925–2174
paths 12 7 paths 21 4
adjacent 1 contiguous 1
subtree distance 0
window 1925–2174
paths 12 7 paths 21 4
adjacent 1 contiguous 1
subtree distance 0
window 1925–2174
paths 12 5 paths 21 2
adjacent 1 contiguous 1
subtree distance 0
window 1950–2199
paths 12 5 paths 21 2
adjacent 1 contiguous 1
subtree distance 0
window 1950–2199
paths 12 9 paths 21 0
adjacent 1 contiguous 1
subtree distance 0
window 1950–2199
paths 12 7 paths 21 4
adjacent 1 contiguous 1
subtree distance 0
window 1975–2224
paths 12 4 paths 21 2
adjacent 1 contiguous 1
subtree distance 0
window 1975–2224
paths 12 9 paths 21 2
adjacent 1 contiguous 1
subtree distance 0
window 1975–2224
paths 12 3 paths 21 3
adjacent 1 contiguous 1
subtree distance 0
window 2000–2249
paths 12 8 paths 21 1
adjacent 1 contiguous 1
subtree distance 0
window 2000–2249
paths 12 5 paths 21 2
adjacent 1 contiguous 1
subtree distance 0
window 2000–2249
paths 12 6 paths 21 2
adjacent 1 contiguous 1
subtree distance 0
window 2025–2274
paths 12 1 paths 21 4
adjacent 1 contiguous 1
subtree distance 0

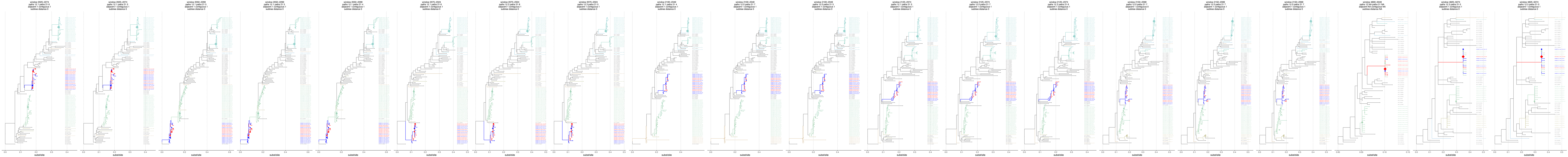
window 2025–2274
paths 12 1 paths 21 6
adjacent 1 contiguous 1
subtree distance 0
window 2025–2274
paths 12 1 paths 21 3
adjacent 1 contiguous 1
subtree distance 0
window 2050–2299
paths 12 1 paths 21 5
adjacent 1 contiguous 1
subtree distance 0
window 2050–2299
paths 12 1 paths 21 5
adjacent 1 contiguous 1
subtree distance 0
window 2050–2299
paths 12 1 paths 21 4
adjacent 1 contiguous 1
subtree distance 0
window 2075–2324
paths 12 1 paths 21 6
adjacent 1 contiguous 1
subtree distance 0
window 2075–2324
paths 12 2 paths 21 8
adjacent 1 contiguous 1
subtree distance 0
window 2075–2324
paths 12 3 paths 21 5
adjacent 1 contiguous 1
subtree distance 0
window 2100–2349
paths 12 1 paths 21 4
adjacent 1 contiguous 1
subtree distance 0
window 2100–2349
paths 12 0 paths 21 7
adjacent 1 contiguous 1
subtree distance 0
window 2100–2349
paths 12 0 paths 21 5
adjacent 1 contiguous 1
subtree distance 0
window 2125–2374
paths 12 1 paths 21 3
adjacent 1 contiguous 1
subtree distance 0
window 2125–2374
paths 12 0 paths 21 7
adjacent 1 contiguous 1
subtree distance 0
window 2125–2374
paths 12 2 paths 21 6
adjacent 1 contiguous 1
subtree distance 0
window 2150–2399
paths 12 0 paths 21 7
adjacent 1 contiguous 0
subtree distance 0
window 2150–2399
paths 12 0 paths 21 7
adjacent 1 contiguous 1
subtree distance 0
window 2150–2399
paths 12 0 paths 21 7
adjacent 1 contiguous 1
subtree distance 0
window 4800–5049
paths 12 NA paths 21 NA
adjacent NA contiguous NA
subtree distance NA
window 4825–5074
paths 12 3 paths 21 0
adjacent 1 contiguous 1
subtree distance 0
window 4825–5074
paths 12 5 paths 21 0
adjacent 1 contiguous 1
subtree distance 0

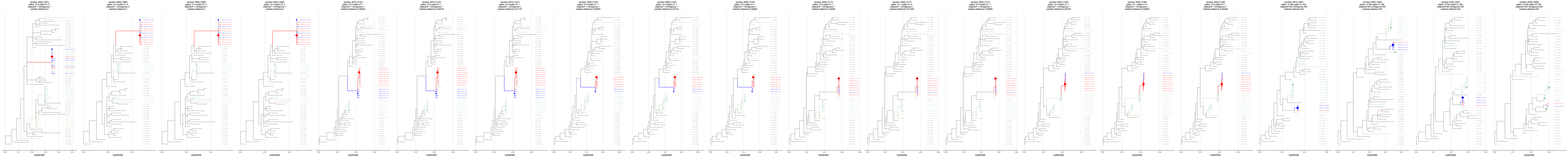
window 4825–5074
paths 12 6 paths 21 0
adjacent 1 contiguous 1
subtree distance 0
window 4850–5099
paths 12 4 paths 21 0
adjacent 1 contiguous 0
subtree distance 0
window 4850–5099
paths 12 4 paths 21 0
adjacent 1 contiguous 1
subtree distance 0
window 4850–5099
paths 12 5 paths 21 0
adjacent 1 contiguous 1
subtree distance 0
window 4875–5124
paths 12 0 paths 21 1
adjacent 1 contiguous 1
subtree distance 0.00634
window 4875–5124
paths 12 0 paths 21 1
adjacent 1 contiguous 1
subtree distance 0.02919
window 4875–5124
paths 12 0 paths 21 1
adjacent 1 contiguous 1
subtree distance 0.01127
window 4900–5149
paths 12 0 paths 21 1
adjacent 1 contiguous 1
subtree distance 0.00605
window 4900–5149
paths 12 0 paths 21 1
adjacent 1 contiguous 1
subtree distance 0.01614
window 4900–5149
paths 12 0 paths 21 1
adjacent 1 contiguous 1
subtree distance 0.00802
window 4925–5174
paths 12 0 paths 21 1
adjacent 1 contiguous 1
subtree distance 0.0056
window 4925–5174
paths 12 1 paths 21 0
adjacent 1 contiguous 1
subtree distance 0.01332
window 4925–5174
paths 12 0 paths 21 1
adjacent 1 contiguous 1
subtree distance 0.00606
window 4950–5199
paths 12 0 paths 21 1
adjacent 1 contiguous 1
subtree distance 0.00476
window 4950–5199
paths 12 1 paths 21 0
adjacent 1 contiguous 1
subtree distance 0.00803
window 4950–5199
paths 12 0 paths 21 1
adjacent 1 contiguous 1
subtree distance 0.00446
window 4975–5224
paths 12 NA paths 21 NA
adjacent NA contiguous NA
subtree distance NA
window 5000–5249
paths 12 NA paths 21 NA
adjacent NA contiguous NA
subtree distance NA
window 5025–5274
paths 12 NA paths 21 NA
adjacent NA contiguous NA
subtree distance NA
window 5050–5299
paths 12 NA paths 21 NA
adjacent NA contiguous NA
subtree distance NA

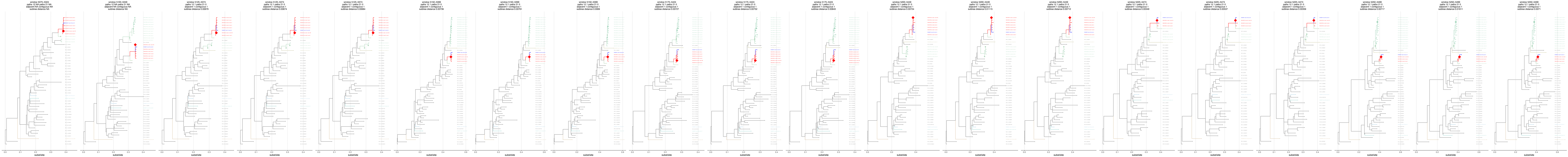
window 5075–5324
paths 12 NA paths 21 NA
adjacent NA contiguous NA
subtree distance NA
window 5100–5349
paths 12 NA paths 21 NA
adjacent NA contiguous NA
subtree distance NA
window 5125–5374
paths 12 1 paths 21 0
adjacent 1 contiguous 1
subtree distance 0.00679
window 5125–5374
paths 12 1 paths 21 0
adjacent 1 contiguous 1
subtree distance 0.00674
window 5125–5374
paths 12 1 paths 21 0
adjacent 1 contiguous 1
subtree distance 0.00684
window 5150–5399
paths 12 1 paths 21 0
adjacent 1 contiguous 1
subtree distance 0.00739
window 5150–5399
paths 12 1 paths 21 0
adjacent 1 contiguous 1
subtree distance 0.00761
window 5150–5399
paths 12 1 paths 21 0
adjacent 1 contiguous 1
subtree distance 0.0069
window 5175–5424
paths 12 1 paths 21 0
adjacent 1 contiguous 1
subtree distance 0.00727
window 5175–5424
paths 12 1 paths 21 0
adjacent 1 contiguous 1
subtree distance 0.00752
window 5175–5424
paths 12 1 paths 21 0
adjacent 1 contiguous 1
subtree distance 0.00843
window 5200–5449
paths 12 1 paths 21 0
adjacent 1 contiguous 1
subtree distance 0.00724
window 5200–5449
paths 12 1 paths 21 0
adjacent 1 contiguous 1
subtree distance 0.01115
window 5200–5449
paths 12 1 paths 21 0
adjacent 1 contiguous 1
subtree distance 0.00712
window 5225–5474
paths 12 1 paths 21 0
adjacent 1 contiguous 1
subtree distance 0.00349
window 5225–5474
paths 12 1 paths 21 0
adjacent 1 contiguous 1
subtree distance 0.00537
window 5225–5474
paths 12 1 paths 21 0
adjacent 1 contiguous 1
subtree distance 0.00359
window 5250–5499
paths 12 1 paths 21 0
adjacent 1 contiguous 1
subtree distance 0.00717
window 5250–5499
paths 12 1 paths 21 0
adjacent 1 contiguous 1
subtree distance 0.01611
window 5250–5499
paths 12 1 paths 21 0
adjacent 1 contiguous 1
subtree distance 0.0071

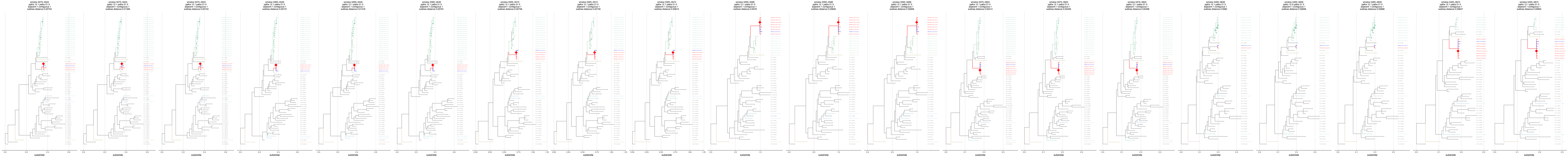
window 5275–5524
paths 12 1 paths 21 0
adjacent 1 contiguous 1
subtree distance 0.00752
window 5275–5524
paths 12 1 paths 21 0
adjacent 1 contiguous 1
subtree distance 0.01395
window 5275–5524
paths 12 1 paths 21 0
adjacent 1 contiguous 1
subtree distance 0.00743
window 5300–5549
paths 12 1 paths 21 0
adjacent 1 contiguous 1
subtree distance 0.00747
window 5300–5549
paths 12 1 paths 21 0
adjacent 1 contiguous 1
subtree distance 0.01479
window 5300–5549
paths 12 1 paths 21 0
adjacent 1 contiguous 1
subtree distance 0.00781
window 5325–5574
paths 12 1 paths 21 0
adjacent 1 contiguous 1
subtree distance 0.00758
window 5325–5574
paths 12 1 paths 21 0
adjacent 1 contiguous 1
subtree distance 0.02749
window 5325–5574
paths 12 1 paths 21 0
adjacent 1 contiguous 1
subtree distance 0.00815
window 5350–5599
paths 12 1 paths 21 0
adjacent 1 contiguous 1
subtree distance 0.0079
window 5350–5599
paths 12 1 paths 21 0
adjacent 1 contiguous 1
subtree distance 0.03629
window 5350–5599
paths 12 1 paths 21 0
adjacent 1 contiguous 1
subtree distance 0.00906
window 5375–5624
paths 12 1 paths 21 0
adjacent 1 contiguous 1
subtree distance 0.00414
window 5375–5624
paths 12 1 paths 21 0
adjacent 1 contiguous 1
subtree distance 0.00409
window 5375–5624
paths 12 1 paths 21 0
adjacent 1 contiguous 1
subtree distance 0.00468
window 5400–5649
paths 12 1 paths 21 0
adjacent 1 contiguous 1
subtree distance 0.0082
window 5400–5649
paths 12 0 paths 21 0
adjacent 1 contiguous 1
subtree distance 0.00836
window 5400–5649
paths 12 1 paths 21 0
adjacent 1 contiguous 1
subtree distance 0.00898
window 5425–5674
paths 12 1 paths 21 0
adjacent 1 contiguous 1
subtree distance 0.00847
window 5425–5674
paths 12 1 paths 21 0
adjacent 1 contiguous 1
subtree distance 0.00855
subst/site

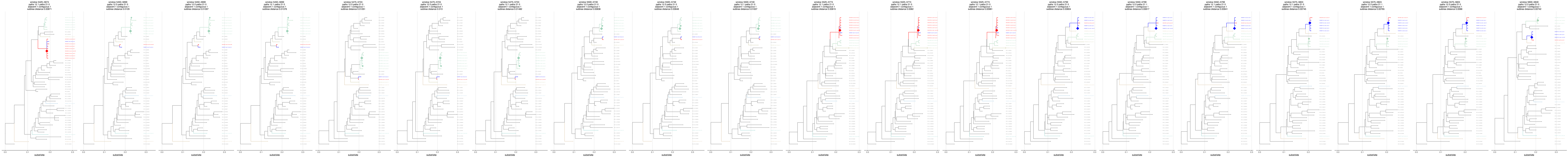
window 5425–5674
paths 12 1 paths 21 0
adjacent 1 contiguous 1
subtree distance 0.00871
window 5450–5699
paths 12 0 paths 21 0
adjacent 1 contiguous 1
subtree distance 0.0129
window 5450–5699
paths 12 0 paths 21 0
adjacent 1 contiguous 1
subtree distance 0.01297
window 5450–5699
paths 12 1 paths 21 0
adjacent 1 contiguous 1
subtree distance 0.01376
window 5475–5724
paths 12 0 paths 21 0
adjacent 1 contiguous 1
subtree distance 0.01261
window 5475–5724
paths 12 0 paths 21 0
adjacent 1 contiguous 1
subtree distance 0.013
window 5475–5724
paths 12 1 paths 21 0
adjacent 1 contiguous 1
subtree distance 0.01482
window 5500–5749
paths 12 0 paths 21 0
adjacent 1 contiguous 1
subtree distance 0.00838
window 5500–5749
paths 12 0 paths 21 0
adjacent 1 contiguous 1
subtree distance 0.00848
window 5500–5749
paths 12 1 paths 21 0
adjacent 1 contiguous 1
subtree distance 0.01007
window 5525–5774
paths 12 1 paths 21 0
adjacent 1 contiguous 1
subtree distance 0.00815
window 5525–5774
paths 12 1 paths 21 0
adjacent 1 contiguous 1
subtree distance 0.0082
window 5525–5774
paths 12 1 paths 21 0
adjacent 1 contiguous 1
subtree distance 0.00981
window 5550–5799
paths 12 0 paths 21 0
adjacent 1 contiguous 1
subtree distance 0.00804
window 5550–5799
paths 12 0 paths 21 1
adjacent 1 contiguous 1
subtree distance 0.00817
window 5550–5799
paths 12 1 paths 21 0
adjacent 1 contiguous 1
subtree distance 0.00941
window 5575–5824
paths 12 1 paths 21 0
adjacent 1 contiguous 1
subtree distance 0.00745
window 5575–5824
paths 12 0 paths 21 1
adjacent 1 contiguous 1
subtree distance 0.00765
window 5575–5824
paths 12 0 paths 21 0
adjacent 1 contiguous 1
subtree distance 0.0088
window 5600–5849
paths 12 0 paths 21 0
adjacent 1 contiguous 1
subtree distance 0.00742
subst/site

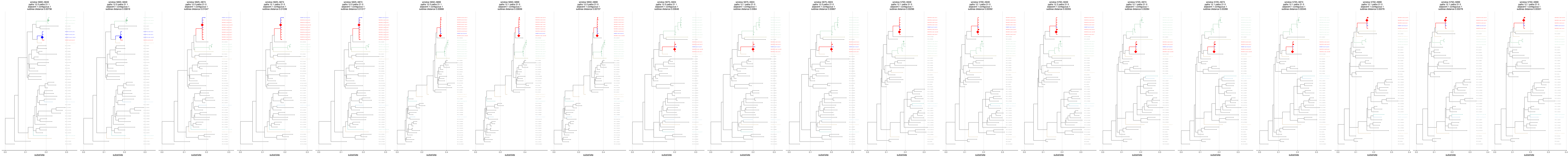

window 5600–5849
paths 12 0 paths 21 1
adjacent 1 contiguous 1
subtree distance 0.00738
window 5600–5849
paths 12 0 paths 21 1
adjacent 1 contiguous 1
subtree distance 0.00819
window 5625–5874
paths 12 0 paths 21 0
adjacent 1 contiguous 1
subtree distance 0.01047
window 5625–5874
paths 12 1 paths 21 0
adjacent 1 contiguous 1
subtree distance 0.01039
window 5625–5874
paths 12 0 paths 21 0
adjacent 1 contiguous 1
subtree distance 0.01317
window 5650–5899
paths 12 0 paths 21 1
adjacent 1 contiguous 1
subtree distance 0.00668
window 5650–5899
paths 12 1 paths 21 0
adjacent 1 contiguous 1
subtree distance 0.00639
window 5650–5899
paths 12 0 paths 21 0
adjacent 1 contiguous 1
subtree distance 0.00737
window 5675–5924
paths 12 0 paths 21 1
adjacent 1 contiguous 1
subtree distance 0.00646
window 5675–5924
paths 12 1 paths 21 0
adjacent 1 contiguous 1
subtree distance 0.0064
window 5675–5924
paths 12 0 paths 21 0
adjacent 1 contiguous 1
subtree distance 0.00667
window 5700–5949
paths 12 1 paths 21 0
adjacent 1 contiguous 1
subtree distance 0.00296
window 5700–5949
paths 12 1 paths 21 0
adjacent 1 contiguous 1
subtree distance 0.00292
window 5700–5949
paths 12 2 paths 21 0
adjacent 1 contiguous 1
subtree distance 0.00355
window 5725–5974
paths 12 1 paths 21 0
adjacent 1 contiguous 1
subtree distance 0.00284
window 5725–5974
paths 12 1 paths 21 0
adjacent 1 contiguous 1
subtree distance 0.00289
window 5725–5974
paths 12 1 paths 21 0
adjacent 1 contiguous 1
subtree distance 0.00345
window 5750–5999
paths 12 1 paths 21 0
adjacent 1 contiguous 1
subtree distance 0.00276
window 5750–5999
paths 12 1 paths 21 0
adjacent 1 contiguous 1
subtree distance 0.00278
window 5750–5999
paths 12 1 paths 21 0
adjacent 1 contiguous 1
subtree distance 0.00321

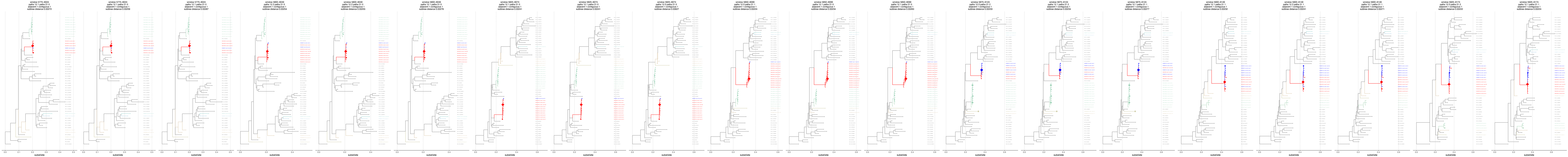
window 5775–6024
paths 12 1 paths 21 0
adjacent 1 contiguous 1
subtree distance 0.00272
window 5775–6024
paths 12 1 paths 21 0
adjacent 1 contiguous 1
subtree distance 0.00259
window 5775–6024
paths 12 1 paths 21 0
adjacent 1 contiguous 1
subtree distance 0.00287
window 5800–6049
paths 12 2 paths 21 0
adjacent 1 contiguous 1
subtree distance 0.00254
window 5800–6049
paths 12 2 paths 21 0
adjacent 1 contiguous 1
subtree distance 0.00255
window 5800–6049
paths 12 1 paths 21 0
adjacent 1 contiguous 1
subtree distance 0.00278
window 5825–6074
paths 12 1 paths 21 0
adjacent 1 contiguous 1
subtree distance 0.00241
window 5825–6074
paths 12 1 paths 21 0
adjacent 1 contiguous 1
subtree distance 0.00241
window 5825–6074
paths 12 0 paths 21 0
adjacent 1 contiguous 1
subtree distance 0.00627
window 5850–6099
paths 12 0 paths 21 1
adjacent 1 contiguous 1
subtree distance 0.00224
window 5850–6099
paths 12 1 paths 21 0
adjacent 1 contiguous 1
subtree distance 0.00234
window 5850–6099
paths 12 1 paths 21 0
adjacent 1 contiguous 1
subtree distance 0.00263
window 5875–6124
paths 12 0 paths 21 2
adjacent 1 contiguous 1
subtree distance 0.00233
window 5875–6124
paths 12 1 paths 21 2
adjacent 1 contiguous 1
subtree distance 0.00234
window 5875–6124
paths 12 1 paths 21 1
adjacent 1 contiguous 1
subtree distance 0.00277
window 5900–6149
paths 12 1 paths 21 1
adjacent 1 contiguous 1
subtree distance 0.00232
window 5900–6149
paths 12 2 paths 21 1
adjacent 1 contiguous 1
subtree distance 0.00241
window 5900–6149
paths 12 1 paths 21 1
adjacent 1 contiguous 1
subtree distance 0.00271
window 5925–6174
paths 12 0 paths 21 2
adjacent 1 contiguous 1
subtree distance 0.00222
window 5925–6174
paths 12 1 paths 21 1
adjacent 1 contiguous 1
subtree distance 0.00224
subst/site

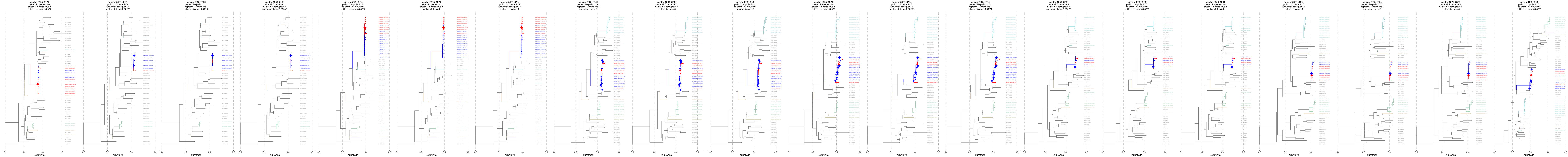
window 5925–6174
paths 12 1 paths 21 0
adjacent 1 contiguous 1
subtree distance 0.0027
window 5950–6199
paths 12 0 paths 21 1
adjacent 1 contiguous 1
subtree distance 0.00225
window 5950–6199
paths 12 0 paths 21 1
adjacent 1 contiguous 1
subtree distance 0.00216
window 5950–6199
paths 12 0 paths 21 1
adjacent 1 contiguous 1
subtree distance 0.00246
window 5975–6224
paths 12 0 paths 21 2
adjacent 1 contiguous 1
subtree distance 0.00227
window 5975–6224
paths 12 1 paths 21 2
adjacent 1 contiguous 1
subtree distance 0
window 5975–6224
paths 12 1 paths 21 1
adjacent 1 contiguous 1
subtree distance 0
window 6000–6249
paths 12 0 paths 21 8
adjacent 1 contiguous 1
subtree distance 0
window 6000–6249
paths 12 0 paths 21 7
adjacent 1 contiguous 1
subtree distance 0
window 6000–6249
paths 12 0 paths 21 4
adjacent 1 contiguous 1
subtree distance 0
window 6025–6274
paths 12 3 paths 21 4
adjacent 1 contiguous 1
subtree distance 0.0025
window 6025–6274
paths 12 2 paths 21 3
adjacent 1 contiguous 1
subtree distance 0.00253
window 6025–6274
paths 12 0 paths 21 3
adjacent 1 contiguous 1
subtree distance 0.00258
window 6050–6299
paths 12 0 paths 21 3
adjacent 1 contiguous 1
subtree distance 0.00525
window 6050–6299
paths 12 0 paths 21 3
adjacent 1 contiguous 1
subtree distance 0.00258
window 6050–6299
paths 12 0 paths 21 4
adjacent 1 contiguous 1
subtree distance 0
window 6075–6324
paths 12 0 paths 21 6
adjacent 1 contiguous 1
subtree distance 0
window 6075–6324
paths 12 0 paths 21 7
adjacent 1 contiguous 1
subtree distance 0
window 6075–6324
paths 12 2 paths 21 6
adjacent 1 contiguous 1
subtree distance 0
window 6100–6349
paths 12 2 paths 21 3
adjacent 1 contiguous 1
subtree distance 0.00265

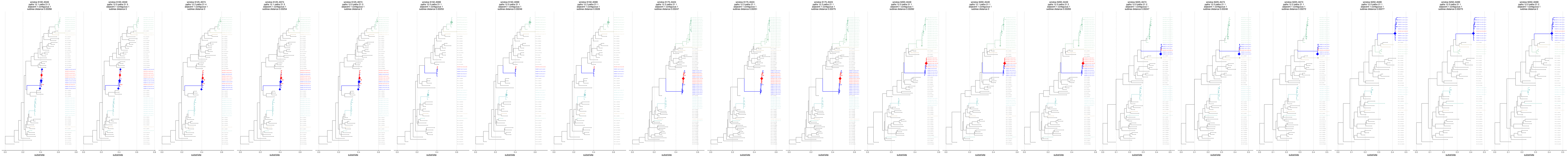
window 6100–6349
paths 12 1 paths 21 3
adjacent 1 contiguous 1
subtree distance 0.00268
window 6100–6349
paths 12 0 paths 21 5
adjacent 1 contiguous 1
subtree distance 0
window 6125–6374
paths 12 2 paths 21 4
adjacent 1 contiguous 1
subtree distance 0
window 6125–6374
paths 12 1 paths 21 3
adjacent 1 contiguous 1
subtree distance 0.00254
window 6125–6374
paths 12 0 paths 21 3
adjacent 1 contiguous 1
subtree distance 0
window 6150–6399
paths 12 0 paths 21 1
adjacent 1 contiguous 1
subtree distance 0.00253
window 6150–6399
paths 12 0 paths 21 1
adjacent 1 contiguous 1
subtree distance 0.00249
window 6150–6399
paths 12 2 paths 21 1
adjacent 1 contiguous 1
subtree distance 0.0028
window 6175–6424
paths 12 2 paths 21 1
adjacent 1 contiguous 1
subtree distance 0.00247
window 6175–6424
paths 12 3 paths 21 1
adjacent 1 contiguous 1
subtree distance 0.00251
window 6175–6424
paths 12 2 paths 21 1
adjacent 1 contiguous 1
subtree distance 0.00275
window 6200–6449
paths 12 0 paths 21 1
adjacent 1 contiguous 1
subtree distance 0.00262
window 6200–6449
paths 12 1 paths 21 1
adjacent 1 contiguous 1
subtree distance 0
window 6200–6449
paths 12 0 paths 21 2
adjacent 1 contiguous 1
subtree distance 0.00263
window 6225–6474
paths 12 0 paths 21 2
adjacent 1 contiguous 1
subtree distance 0.00247
window 6225–6474
paths 12 0 paths 21 1
adjacent 1 contiguous 1
subtree distance 0.00249
window 6225–6474
paths 12 2 paths 21 1
adjacent 1 contiguous 1
subtree distance 0.00272
window 6250–6499
paths 12 0 paths 21 1
adjacent 1 contiguous 1
subtree distance 0.00277
window 6250–6499
paths 12 0 paths 21 1
adjacent 1 contiguous 1
subtree distance 0.00273
window 6250–6499
paths 12 0 paths 21 2
adjacent 1 contiguous 1
subtree distance 0

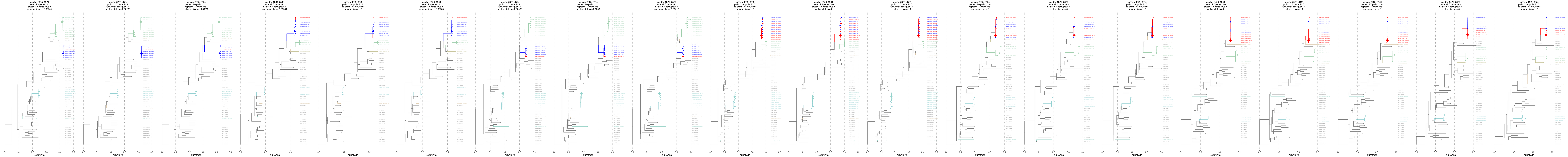
window 6275–6524
paths 12 0 paths 21 1
adjacent 1 contiguous 1
subtree distance 0.00249
window 6275–6524
paths 12 0 paths 21 1
adjacent 1 contiguous 1
subtree distance 0.00236
window 6275–6524
paths 12 0 paths 21 1
adjacent 1 contiguous 1
subtree distance 0.00259
window 6300–6549
paths 12 0 paths 21 1
adjacent 1 contiguous 1
subtree distance 0.00234
window 6300–6549
paths 12 0 paths 21 2
adjacent 1 contiguous 1
subtree distance 0
window 6300–6549
paths 12 0 paths 21 1
adjacent 1 contiguous 1
subtree distance 0.00265
window 6325–6574
paths 12 0 paths 21 1
adjacent 1 contiguous 1
subtree distance 0.00466
window 6325–6574
paths 12 0 paths 21 1
adjacent 1 contiguous 1
subtree distance 0.0048
window 6325–6574
paths 12 0 paths 21 1
adjacent 1 contiguous 1
subtree distance 0.00519
window 6350–6599
paths 12 6 paths 21 0
adjacent 1 contiguous 1
subtree distance 0
window 6350–6599
paths 12 5 paths 21 0
adjacent 1 contiguous 1
subtree distance 0
window 6350–6599
paths 12 5 paths 21 0
adjacent 1 contiguous 1
subtree distance 0
window 6375–6624
paths 12 6 paths 21 0
adjacent 1 contiguous 1
subtree distance 0
window 6375–6624
paths 12 4 paths 21 0
adjacent 1 contiguous 1
subtree distance 0
window 6375–6624
paths 12 6 paths 21 0
adjacent 1 contiguous 1
subtree distance 0
window 6400–6649
paths 12 7 paths 21 0
adjacent 1 contiguous 1
subtree distance 0
window 6400–6649
paths 12 7 paths 21 0
adjacent 1 contiguous 1
subtree distance 0
window 6400–6649
paths 12 7 paths 21 0
adjacent 1 contiguous 1
subtree distance 0
window 6425–6674
paths 12 6 paths 21 0
adjacent 1 contiguous 1
subtree distance 0
window 6425–6674
paths 12 6 paths 21 0
adjacent 1 contiguous 1
subtree distance 0

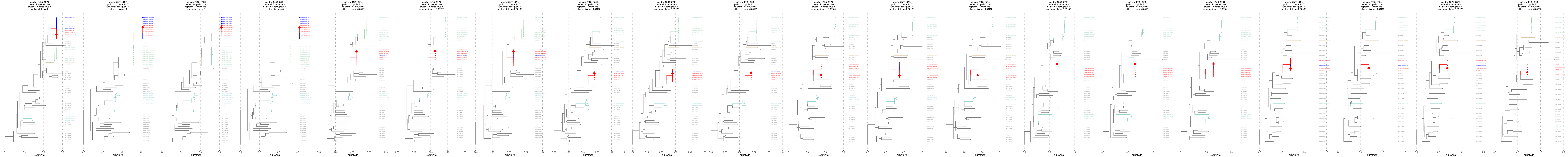
window 6425–6674
paths 12 6 paths 21 0
adjacent 1 contiguous 1
subtree distance 0
window 6450–6699
paths 12 5 paths 21 0
adjacent 1 contiguous 1
subtree distance 0
window 6450–6699
paths 12 4 paths 21 0
adjacent 1 contiguous 1
subtree distance 0
window 6450–6699
paths 12 3 paths 21 0
adjacent 1 contiguous 1
subtree distance 0
window 6475–6724
paths 12 1 paths 21 0
adjacent 1 contiguous 1
subtree distance 0.00181
window 6475–6724
paths 12 1 paths 21 0
adjacent 1 contiguous 1
subtree distance 0.00172
window 6475–6724
paths 12 1 paths 21 0
adjacent 1 contiguous 1
subtree distance 0.00193
window 6500–6749
paths 12 1 paths 21 0
adjacent 1 contiguous 1
subtree distance 0.00176
window 6500–6749
paths 12 1 paths 21 0
adjacent 1 contiguous 1
subtree distance 0.00177
window 6500–6749
paths 12 1 paths 21 0
adjacent 1 contiguous 1
subtree distance 0.00176
window 6525–6774
paths 12 1 paths 21 0
adjacent 1 contiguous 1
subtree distance 0.00169
window 6525–6774
paths 12 1 paths 21 0
adjacent 1 contiguous 1
subtree distance 0.00163
window 6525–6774
paths 12 1 paths 21 0
adjacent 1 contiguous 1
subtree distance 0.00162
window 6550–6799
paths 12 1 paths 21 0
adjacent 1 contiguous 1
subtree distance 0.00155
window 6550–6799
paths 12 1 paths 21 0
adjacent 1 contiguous 1
subtree distance 0.00151
window 6550–6799
paths 12 1 paths 21 0
adjacent 1 contiguous 1
subtree distance 0.00161
window 6575–6824
paths 12 1 paths 21 0
adjacent 1 contiguous 1
subtree distance 0.00493
window 6575–6824
paths 12 1 paths 21 0
adjacent 1 contiguous 1
subtree distance 0.00165
window 6575–6824
paths 12 1 paths 21 0
adjacent 1 contiguous 1
subtree distance 0.00175
window 6600–6849
paths 12 1 paths 21 0
adjacent 1 contiguous 1
subtree distance 0.00537
subst/site

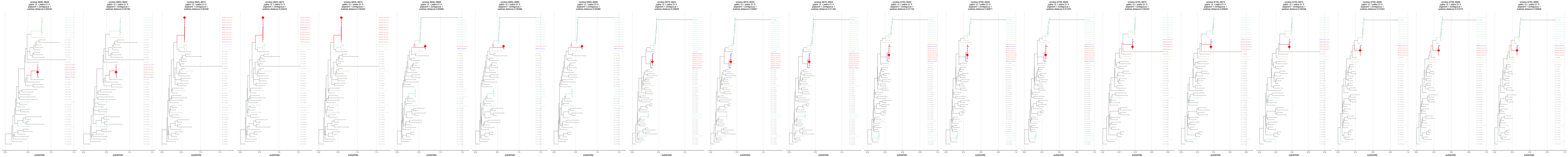
window 6600–6849
paths 12 1 paths 21 0
adjacent 1 contiguous 1
subtree distance 0.00549
window 6600–6849
paths 12 1 paths 21 0
adjacent 1 contiguous 1
subtree distance 0.00183
window 6625–6874
paths 12 1 paths 21 0
adjacent 1 contiguous 1
subtree distance 0.00439
window 6625–6874
paths 12 1 paths 21 0
adjacent 1 contiguous 1
subtree distance 0.00443
window 6625–6874
paths 12 1 paths 21 0
adjacent 1 contiguous 1
subtree distance 0.00424
window 6650–6899
paths 12 1 paths 21 0
adjacent 1 contiguous 1
subtree distance 0.00385
window 6650–6899
paths 12 1 paths 21 0
adjacent 1 contiguous 1
subtree distance 0.00386
window 6650–6899
paths 12 1 paths 21 0
adjacent 1 contiguous 1
subtree distance 0.00386
window 6675–6924
paths 12 1 paths 21 0
adjacent 1 contiguous 1
subtree distance 0.00836
window 6675–6924
paths 12 1 paths 21 0
adjacent 1 contiguous 1
subtree distance 0.00847
window 6675–6924
paths 12 1 paths 21 0
adjacent 1 contiguous 1
subtree distance 0.01163
window 6700–6949
paths 12 1 paths 21 0
adjacent 1 contiguous 1
subtree distance 0.01105
window 6700–6949
paths 12 1 paths 21 0
adjacent 1 contiguous 1
subtree distance 0.00871
window 6700–6949
paths 12 1 paths 21 0
adjacent 1 contiguous 1
subtree distance 0.01233
window 6725–6974
paths 12 1 paths 21 0
adjacent 1 contiguous 1
subtree distance 0.00243
window 6725–6974
paths 12 1 paths 21 0
adjacent 1 contiguous 1
subtree distance 0.00926
window 6725–6974
paths 12 1 paths 21 0
adjacent 1 contiguous 1
subtree distance 0.00246
window 6750–6999
paths 12 1 paths 21 0
adjacent 1 contiguous 1
subtree distance 0.01054
window 6750–6999
paths 12 1 paths 21 0
adjacent 1 contiguous 1
subtree distance 0.01073
window 6750–6999
paths 12 1 paths 21 0
adjacent 1 contiguous 1
subtree distance 0.00856

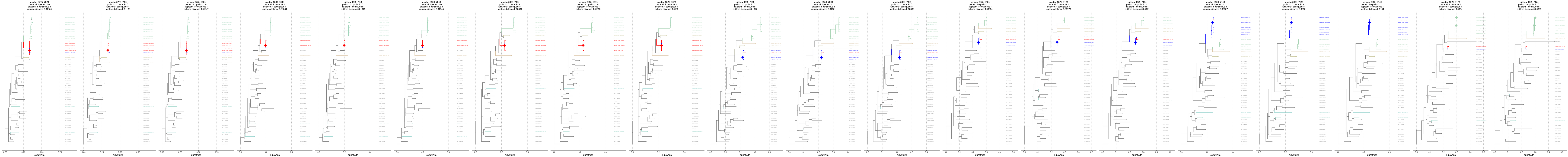
window 6775–7024
paths 12 1 paths 21 0
adjacent 1 contiguous 1
subtree distance 0.01164
subst/site
window 6775–7024
paths 12 1 paths 21 0
adjacent 1 contiguous 1
subtree distance 0.01183
subst/site
window 6775–7024
paths 12 1 paths 21 0
adjacent 1 contiguous 1
subtree distance 0.01247
subst/site
window 6800–7049
paths 12 2 paths 21 0
adjacent 1 contiguous 1
subtree distance 0.01034
subst/site
window 6800–7049
paths 12 1 paths 21 0
adjacent 1 contiguous 1
subtree distance 0.01016
subst/site
window 6800–7049
paths 12 1 paths 21 0
adjacent 1 contiguous 1
subtree distance 0.01345
subst/site
window 6825–7074
paths 12 0 paths 21 1
adjacent 1 contiguous 1
subtree distance 0.00801
subst/site
window 6825–7074
paths 12 1 paths 21 0
adjacent 1 contiguous 1
subtree distance 0.01059
subst/site
window 6825–7074
paths 12 2 paths 21 0
adjacent 1 contiguous 1
subtree distance 0.0132
subst/site
window 6850–7099
paths 12 2 paths 21 0
adjacent 1 contiguous 1
subtree distance 0.01047
subst/site
window 6850–7099
paths 12 0 paths 21 1
adjacent 1 contiguous 1
subtree distance 0.01021
subst/site
window 6850–7099
paths 12 1 paths 21 0
adjacent 1 contiguous 1
subtree distance 0.00309
subst/site
window 6875–7124
paths 12 0 paths 21 1
adjacent 1 contiguous 1
subtree distance 0.00804
subst/site
window 6875–7124
paths 12 0 paths 21 1
adjacent 1 contiguous 1
subtree distance 0.00773
subst/site
window 6875–7124
paths 12 0 paths 21 1
adjacent 1 contiguous 1
subtree distance 0.00941
subst/site
window 6900–7149
paths 12 0 paths 21 1
adjacent 1 contiguous 1
subtree distance 0.00827
subst/site
window 6900–7149
paths 12 0 paths 21 1
adjacent 1 contiguous 1
subtree distance 0.0082
subst/site
window 6900–7149
paths 12 0 paths 21 1
adjacent 1 contiguous 1
subtree distance 0.0104
subst/site
window 6925–7174
paths 12 1 paths 21 0
adjacent 1 contiguous 1
subtree distance 0.0054
subst/site
window 6925–7174
paths 12 0 paths 21 0
adjacent 1 contiguous 1
subtree distance 0.00823
subst/site

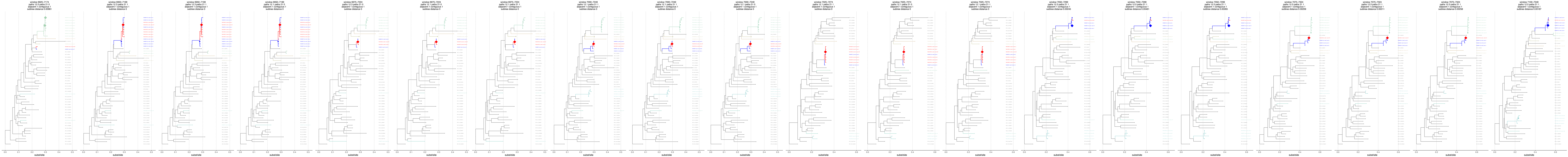
window 6925–7174
paths 12 0 paths 21 0
adjacent 1 contiguous 1
subtree distance 0.00981
window 6950–7199
paths 12 3 paths 21 1
adjacent 1 contiguous 1
subtree distance 0
window 6950–7199
paths 12 3 paths 21 1
adjacent 1 contiguous 1
subtree distance 0
window 6950–7199
paths 12 1 paths 21 0
adjacent 1 contiguous 1
subtree distance 0
window 6975–7224
paths 12 3 paths 21 0
adjacent 1 contiguous 1
subtree distance 0
window 6975–7224
paths 12 1 paths 21 0
adjacent 1 contiguous 1
subtree distance 0
window 6975–7224
paths 12 1 paths 21 1
adjacent 1 contiguous 1
subtree distance 0
window 7000–7249
paths 12 1 paths 21 1
adjacent 1 contiguous 1
subtree distance 0
window 7000–7249
paths 12 1 paths 21 1
adjacent 1 contiguous 1
subtree distance 0
window 7000–7249
paths 12 1 paths 21 0
adjacent 1 contiguous 1
subtree distance 0
window 7025–7274
paths 12 1 paths 21 1
adjacent 1 contiguous 1
subtree distance 0
window 7025–7274
paths 12 1 paths 21 0
adjacent 1 contiguous 1
subtree distance 0
window 7025–7274
paths 12 1 paths 21 1
adjacent 1 contiguous 1
subtree distance 0
window 7050–7299
paths 12 0 paths 21 1
adjacent 1 contiguous 1
subtree distance 0.00245
window 7050–7299
paths 12 0 paths 21 1
adjacent 1 contiguous 1
subtree distance 0.00481
window 7050–7299
paths 12 0 paths 21 1
adjacent 1 contiguous 1
subtree distance 0.00265
window 7075–7324
paths 12 0 paths 21 1
adjacent 1 contiguous 1
subtree distance 0.00209
window 7075–7324
paths 12 0 paths 21 1
adjacent 1 contiguous 1
subtree distance 0.00211
window 7075–7324
paths 12 0 paths 21 1
adjacent 1 contiguous 1
subtree distance 0.00258
window 7100–7349
paths 12 0 paths 21 1
adjacent 1 contiguous 1
subtree distance 0.00187
subst/site

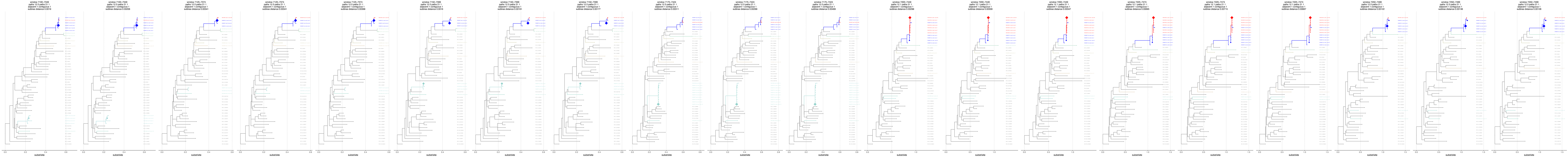
window 7100–7349
paths 12 0 paths 21 1
adjacent 1 contiguous 1
subtree distance 0.0019
window 7100–7349
paths 12 0 paths 21 1
adjacent 1 contiguous 1
subtree distance 0.00206
window 7125–7374
paths 12 0 paths 21 1
adjacent 1 contiguous 1
subtree distance 0.00341
window 7125–7374
paths 12 0 paths 21 1
adjacent 1 contiguous 1
subtree distance 0.00341
window 7125–7374
paths 12 0 paths 21 1
adjacent 1 contiguous 1
subtree distance 0.00368
window 7150–7399
paths 12 0 paths 21 1
adjacent 1 contiguous 1
subtree distance 0.0016
window 7150–7399
paths 12 0 paths 21 1
adjacent 1 contiguous 1
subtree distance 0.00161
window 7150–7399
paths 12 0 paths 21 1
adjacent 1 contiguous 1
subtree distance 0.00177
window 7175–7424
paths 12 0 paths 21 1
adjacent 1 contiguous 1
subtree distance 0.00311
window 7175–7424
paths 12 0 paths 21 1
adjacent 1 contiguous 1
subtree distance 0.00299
window 7175–7424
paths 12 0 paths 21 1
adjacent 1 contiguous 1
subtree distance 0.00469
window 7200–7449
paths 12 1 paths 21 1
adjacent 1 contiguous 1
subtree distance 0.00298
window 7200–7449
paths 12 1 paths 21 1
adjacent 1 contiguous 1
subtree distance 0.00309
window 7200–7449
paths 12 1 paths 21 1
adjacent 1 contiguous 1
subtree distance 0.00347
window 7225–7474
paths 12 1 paths 21 1
adjacent 1 contiguous 1
subtree distance 0.00695
window 7225–7474
paths 12 1 paths 21 1
adjacent 1 contiguous 1
subtree distance 0.00715
window 7225–7474
paths 12 1 paths 21 1
adjacent 1 contiguous 1
subtree distance 0.00667
window 7250–7499
paths 12 0 paths 21 1
adjacent 1 contiguous 1
subtree distance 0.00122
window 7250–7499
paths 12 0 paths 21 1
adjacent 1 contiguous 1
subtree distance 0.00136
window 7250–7499
paths 12 0 paths 21 1
adjacent 1 contiguous 1
subtree distance 0.00128

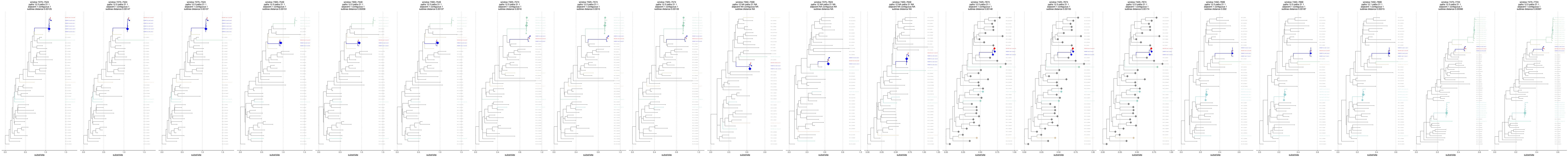
window 7275–7524
paths 12 0 paths 21 1
adjacent 1 contiguous 1
subtree distance 0.00125
window 7275–7524
paths 12 0 paths 21 1
adjacent 1 contiguous 1
subtree distance 0.00147
window 7275–7524
paths 12 0 paths 21 1
adjacent 1 contiguous 1
subtree distance 0.00137
window 7300–7549
paths 12 0 paths 21 1
adjacent 1 contiguous 1
subtree distance 0.00151
window 7300–7549
paths 12 0 paths 21 1
adjacent 1 contiguous 1
subtree distance 0.00209
window 7300–7549
paths 12 0 paths 21 1
adjacent 1 contiguous 1
subtree distance 0.00137
window 7325–7574
paths 12 0 paths 21 1
adjacent 1 contiguous 1
subtree distance 0.0016
window 7325–7574
paths 12 0 paths 21 1
adjacent 1 contiguous 1
subtree distance 0.0015
window 7325–7574
paths 12 0 paths 21 1
adjacent 1 contiguous 1
subtree distance 0.00133
window 7350–7599
paths 12 NA paths 21 NA
adjacent NA contiguous NA
subtree distance NA
window 7375–7624
paths 12 NA paths 21 NA
adjacent NA contiguous NA
subtree distance NA
window 7400–7649
paths 12 NA paths 21 NA
adjacent NA contiguous NA
subtree distance NA
window 7425–7674
paths 12 0 paths 21 1
adjacent 1 contiguous 1
subtree distance 0.00148
window 7425–7674
paths 12 0 paths 21 1
adjacent 1 contiguous 1
subtree distance 0.00155
window 7425–7674
paths 12 0 paths 21 1
adjacent 1 contiguous 1
subtree distance 0.00174
window 7450–7699
paths 12 0 paths 21 1
adjacent 1 contiguous 1
subtree distance 0.002
window 7450–7699
paths 12 0 paths 21 1
adjacent 1 contiguous 1
subtree distance 0.00187
window 7450–7699
paths 12 1 paths 21 1
adjacent 1 contiguous 1
subtree distance 0.00215
window 7475–7724
paths 12 0 paths 21 1
adjacent 1 contiguous 1
subtree distance 0.00396
window 7475–7724
paths 12 0 paths 21 1
adjacent 1 contiguous 1
subtree distance 0.00397

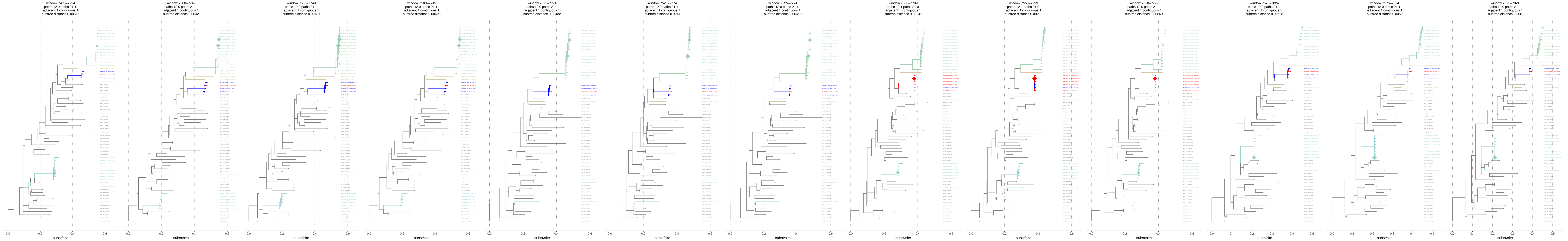

window 7475–7724
paths 12 0 paths 21 1
adjacent 1 contiguous 1
subtree distance 0.00355
window 7500–7749
paths 12 0 paths 21 1
adjacent 1 contiguous 1
subtree distance 0.0042
window 7500–7749
paths 12 0 paths 21 1
adjacent 1 contiguous 1
subtree distance 0.00431
window 7500–7749
paths 12 0 paths 21 1
adjacent 1 contiguous 1
subtree distance 0.00423
window 7525–7774
paths 12 0 paths 21 1
adjacent 1 contiguous 1
subtree distance 0.00432
window 7525–7774
paths 12 0 paths 21 1
adjacent 1 contiguous 1
subtree distance 0.0044
window 7525–7774
paths 12 0 paths 21 1
adjacent 1 contiguous 1
subtree distance 0.00419
window 7550–7799
paths 12 1 paths 21 0
adjacent 1 contiguous 1
subtree distance 0.00241
window 7550–7799
paths 12 1 paths 21 0
adjacent 1 contiguous 1
subtree distance 0.00239
window 7550–7799
paths 12 0 paths 21 1
adjacent 1 contiguous 1
subtree distance 0.00269
window 7575–7824
paths 12 0 paths 21 1
adjacent 1 contiguous 1
subtree distance 0.00523
window 7575–7824
paths 12 0 paths 21 1
adjacent 1 contiguous 1
subtree distance 0.0052
window 7575–7824
paths 12 0 paths 21 1
adjacent 1 contiguous 1
subtree distance 0.006
subst/site

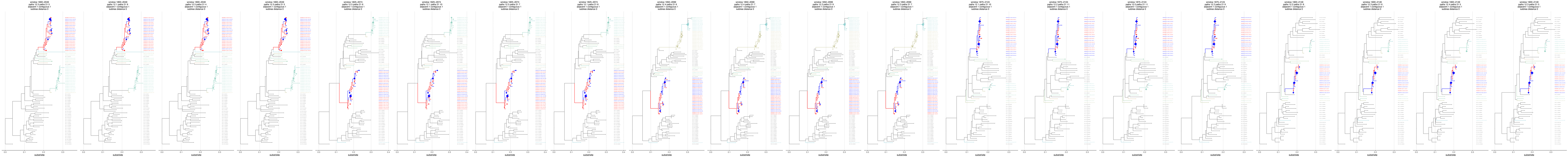
window 1800–2049
paths 12 5 paths 21 5
adjacent 1 contiguous 1
subtree distance 0
window 1800–2049
paths 12 1 paths 21 9
adjacent 1 contiguous 1
subtree distance 0
window 1800–2049
paths 12 5 paths 21 4
adjacent 1 contiguous 1
subtree distance 0
window 1800–2049
paths 12 5 paths 21 5
adjacent 1 contiguous 1
subtree distance 0
window 1825–2074
paths 12 5 paths 21 6
adjacent 1 contiguous 1
subtree distance 0
window 1825–2074
paths 12 1 paths 21 10
adjacent 1 contiguous 1
subtree distance 0
window 1825–2074
paths 12 5 paths 21 7
adjacent 1 contiguous 1
subtree distance 0
window 1825–2074
paths 12 1 paths 21 8
adjacent 1 contiguous 1
subtree distance 0
window 1850–2099
paths 12 4 paths 21 6
adjacent 1 contiguous 1
subtree distance 0
window 1850–2099
paths 12 4 paths 21 7
adjacent 1 contiguous 1
subtree distance 0
window 1850–2099
paths 12 2 paths 21 9
adjacent 1 contiguous 1
subtree distance 0
window 1850–2099
paths 12 3 paths 21 7
adjacent 1 contiguous 1
subtree distance 0
window 1875–2124
paths 12 1 paths 21 10
adjacent 1 contiguous 1
subtree distance 0
window 1875–2124
paths 12 2 paths 21 11
adjacent 1 contiguous 1
subtree distance 0
window 1875–2124
paths 12 0 paths 21 11
adjacent 1 contiguous 1
subtree distance 0
window 1875–2124
paths 12 2 paths 21 9
adjacent 1 contiguous 1
subtree distance 0
window 1900–2149
paths 12 2 paths 21 6
adjacent 1 contiguous 1
subtree distance 0
window 1900–2149
paths 12 3 paths 21 6
adjacent 1 contiguous 1
subtree distance 0
window 1900–2149
paths 12 4 paths 21 5
adjacent 1 contiguous 1
subtree distance 0
window 1900–2149
paths 12 3 paths 21 5
adjacent 1 contiguous 1
subtree distance 0
0.0
0.1
0.2
0.3
subst/site

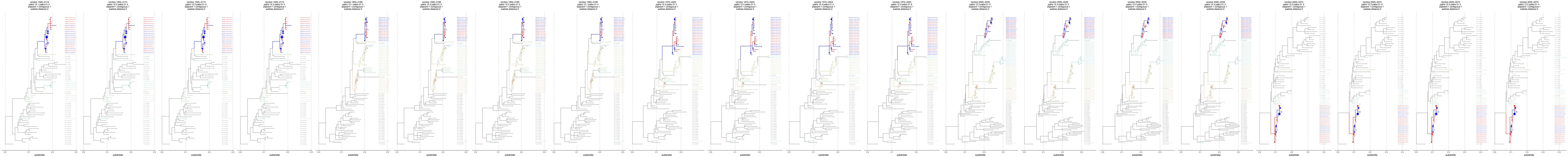
window 1925–2174
paths 12 1 paths 21 5
adjacent 1 contiguous 1
subtree distance 0
window 1925–2174
paths 12 2 paths 21 4
adjacent 1 contiguous 1
subtree distance 0
window 1925–2174
paths 12 2 paths 21 5
adjacent 1 contiguous 1
subtree distance 0
window 1925–2174
paths 12 2 paths 21 5
adjacent 1 contiguous 1
subtree distance 0
window 1950–2199
paths 12 1 paths 21 2
adjacent 1 contiguous 1
subtree distance 0
window 1950–2199
paths 12 2 paths 21 3
adjacent 1 contiguous 1
subtree distance 0
window 1950–2199
paths 12 2 paths 21 5
adjacent 1 contiguous 1
subtree distance 0
window 1950–2199
paths 12 1 paths 21 4
adjacent 1 contiguous 1
subtree distance 0
window 1975–2224
paths 12 4 paths 21 5
adjacent 1 contiguous 1
subtree distance 0
window 1975–2224
paths 12 4 paths 21 5
adjacent 1 contiguous 1
subtree distance 0
window 1975–2224
paths 12 4 paths 21 5
adjacent 1 contiguous 1
subtree distance 0
window 1975–2224
paths 12 3 paths 21 6
adjacent 1 contiguous 1
subtree distance 0
window 2000–2249
paths 12 3 paths 21 2
adjacent 1 contiguous 1
subtree distance 0
window 2000–2249
paths 12 4 paths 21 3
adjacent 1 contiguous 1
subtree distance 0
window 2000–2249
paths 12 4 paths 21 2
adjacent 1 contiguous 1
subtree distance 0
window 2000–2249
paths 12 4 paths 21 2
adjacent 1 contiguous 1
subtree distance 0
window 2025–2274
paths 12 5 paths 21 3
adjacent 1 contiguous 1
subtree distance 0
window 2025–2274
paths 12 5 paths 21 3
adjacent 1 contiguous 1
subtree distance 0
window 2025–2274
paths 12 5 paths 21 2
adjacent 1 contiguous 1
subtree distance 0
window 2025–2274
paths 12 5 paths 21 4
adjacent 1 contiguous 1
subtree distance 0
subst/site

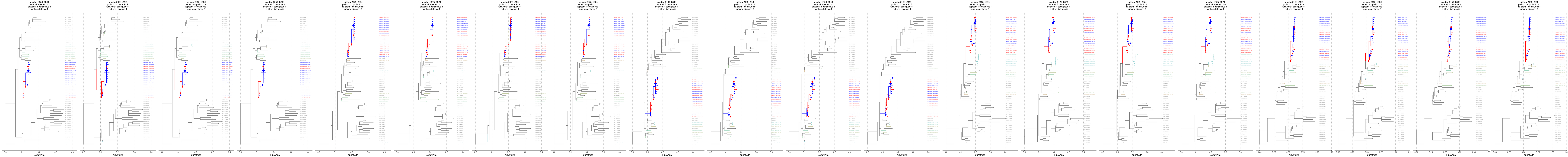
window 2050–2299
paths 12 4 paths 21 2
adjacent 1 contiguous 1
subtree distance 0
window 2050–2299
paths 12 4 paths 21 2
adjacent 1 contiguous 1
subtree distance 0
window 2050–2299
paths 12 4 paths 21 4
adjacent 1 contiguous 1
subtree distance 0
window 2050–2299
paths 12 6 paths 21 2
adjacent 1 contiguous 1
subtree distance 0
window 2075–2324
paths 12 3 paths 21 4
adjacent 1 contiguous 1
subtree distance 0
window 2075–2324
paths 12 4 paths 21 1
adjacent 1 contiguous 1
subtree distance 0
window 2075–2324
paths 12 5 paths 21 1
adjacent 1 contiguous 1
subtree distance 0
window 2075–2324
paths 12 4 paths 21 1
adjacent 1 contiguous 1
subtree distance 0
window 2100–2349
paths 12 3 paths 21 9
adjacent 1 contiguous 1
subtree distance 0
window 2100–2349
paths 12 3 paths 21 8
adjacent 1 contiguous 1
subtree distance 0
window 2100–2349
paths 12 2 paths 21 7
adjacent 1 contiguous 1
subtree distance 0
window 2100–2349
paths 12 3 paths 21 8
adjacent 1 contiguous 1
subtree distance 0
window 2125–2374
paths 12 2 paths 21 7
adjacent 1 contiguous 1
subtree distance 0
window 2125–2374
paths 12 3 paths 21 5
adjacent 1 contiguous 1
subtree distance 0
window 2125–2374
paths 12 3 paths 21 8
adjacent 1 contiguous 1
subtree distance 0
window 2125–2374
paths 12 3 paths 21 8
adjacent 1 contiguous 1
subtree distance 0
window 2150–2399
paths 12 3 paths 21 7
adjacent 1 contiguous 1
subtree distance 0
window 2150–2399
paths 12 2 paths 21 5
adjacent 1 contiguous 1
subtree distance 0
window 2150–2399
paths 12 4 paths 21 2
adjacent 1 contiguous 1
subtree distance 0
window 2150–2399
paths 12 4 paths 21 2
adjacent 1 contiguous 1
subtree distance 0
subst/site
