# Supplementary material for: Inferring HIV-1 transmission networks and sources of epidemic spread in Africa with deep-sequence phylogenetic analysis
Source: Nat Commun. 2019 Mar 29;10:1411. doi: 10.1038/s41467-019-09139-4 (PMC6441045; doi:10.1038/s41467-019-09139-4)
Supplement: Supplementary file 4 — Supplementary Data 2 [file 41467_2019_9139_MOESM4_ESM.pdf]

Deep sequence phylogenies
of male-female pair RkA05162M, RkA02020F (run 10)
for whom the phylogenetically inferred direction of transmission was inconsistent with clinical data.

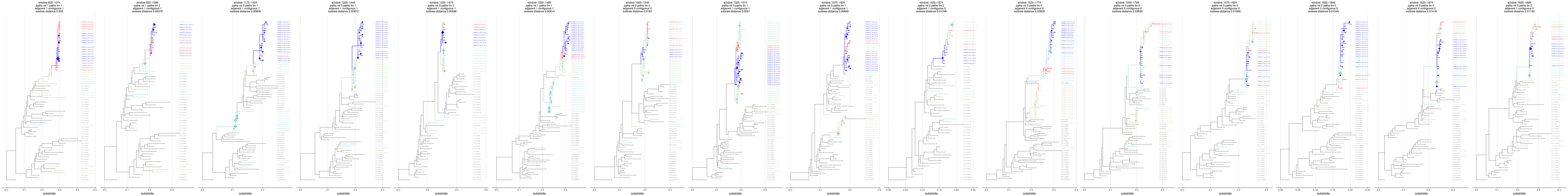
window 825–1074
paths mf 1 paths fm 1
adjacent 1 contiguous 1
subtree distance 0.005
window 850–1099
paths mf 1 paths fm 2
adjacent 1 contiguous 1
subtree distance 0.00379
window 1175–1424
paths mf 0 paths fm 1
adjacent 1 contiguous 1
subtree distance 0.00806
window 1200–1449
paths mf 0 paths fm 1
adjacent 1 contiguous 1
subtree distance 0.00872
window 1225–1474
paths mf 0 paths fm 2
adjacent 1 contiguous 1
subtree distance 0.00596
window 1250–1499
paths mf 1 paths fm 1
adjacent 1 contiguous 1
subtree distance 0.00614
window 1300–1549
paths mf 0 paths fm 4
adjacent 0 contiguous 0
subtree distance 0.0162
window 1325–1574
paths mf 0 paths fm 1
adjacent 1 contiguous 1
subtree distance 0.0057
window 1375–1624
paths mf 0 paths fm 1
adjacent 1 contiguous 1
subtree distance 0.00609
window 1425–1674
paths mf 0 paths fm 2
adjacent 1 contiguous 0
subtree distance 0.01248
window 1525–1774
paths mf 5 paths fm 0
adjacent 0 contiguous 0
subtree distance 0.03929
window 1550–1799
paths mf 0 paths fm 0
adjacent 0 contiguous 0
subtree distance 0.03635
window 1575–1824
paths mf 0 paths fm 0
adjacent 0 contiguous 0
subtree distance 0.01806
window 1600–1849
paths mf 0 paths fm 0
adjacent 0 contiguous 0
subtree distance 0.01549
window 1625–1874
paths mf 0 paths fm 0
adjacent 0 contiguous 0
subtree distance 0.01418
window 1650–1899
paths mf 0 paths fm 2
adjacent 1 contiguous 0
subtree distance 0.01135
subst/site

Deep sequence phylogenies
of male-female pair RkA00495M, RkA07422F (run 12)
for whom the phylogenetically inferred direction of transmission was inconsistent with clinical data.

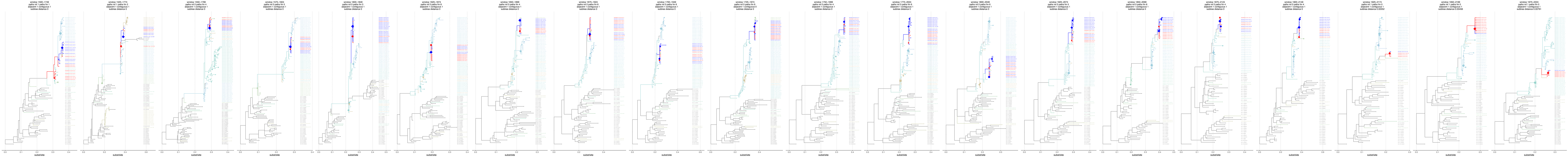
window 1500–1749
paths mf 1 paths fm 1
adjacent 1 contiguous 1
subtree distance 0
window 1525–1774
paths mf 1 paths fm 2
adjacent 1 contiguous 1
subtree distance 0
window 1550–1799
paths mf 0 paths fm 4
adjacent 1 contiguous 1
subtree distance 0
window 1575–1824
paths mf 0 paths fm 5
adjacent 1 contiguous 1
subtree distance 0
window 1600–1849
paths mf 0 paths fm 3
adjacent 1 contiguous 1
subtree distance 0
window 1625–1874
paths mf 0 paths fm 8
adjacent 1 contiguous 1
subtree distance 0
window 1650–1899
paths mf 0 paths fm 4
adjacent 1 contiguous 1
subtree distance 0
window 1675–1924
paths mf 0 paths fm 8
adjacent 1 contiguous 1
subtree distance 0
window 1700–1949
paths mf 0 paths fm 6
adjacent 1 contiguous 1
subtree distance 0
window 1725–1974
paths mf 0 paths fm 9
adjacent 1 contiguous 0
subtree distance 0
window 1750–1999
paths mf 0 paths fm 4
adjacent 1 contiguous 1
subtree distance 0
window 1775–2024
paths mf 0 paths fm 6
adjacent 1 contiguous 1
subtree distance 0
window 1800–2049
paths mf 0 paths fm 6
adjacent 1 contiguous 1
subtree distance 0
window 1825–2074
paths mf 0 paths fm 5
adjacent 1 contiguous 1
subtree distance 0
window 1850–2099
paths mf 0 paths fm 8
adjacent 1 contiguous 1
subtree distance 0
window 1875–2124
paths mf 0 paths fm 4
adjacent 1 contiguous 1
subtree distance 0
window 1900–2149
paths mf 0 paths fm 4
adjacent 1 contiguous 1
subtree distance 0
window 1925–2174
paths mf 1 paths fm 0
adjacent 1 contiguous 1
subtree distance 0.00352
window 1950–2199
paths mf 1 paths fm 0
adjacent 1 contiguous 1
subtree distance 0.00456
window 1975–2224
paths mf 1 paths fm 0
adjacent 1 contiguous 1
subtree distance 0.00791
subst/site

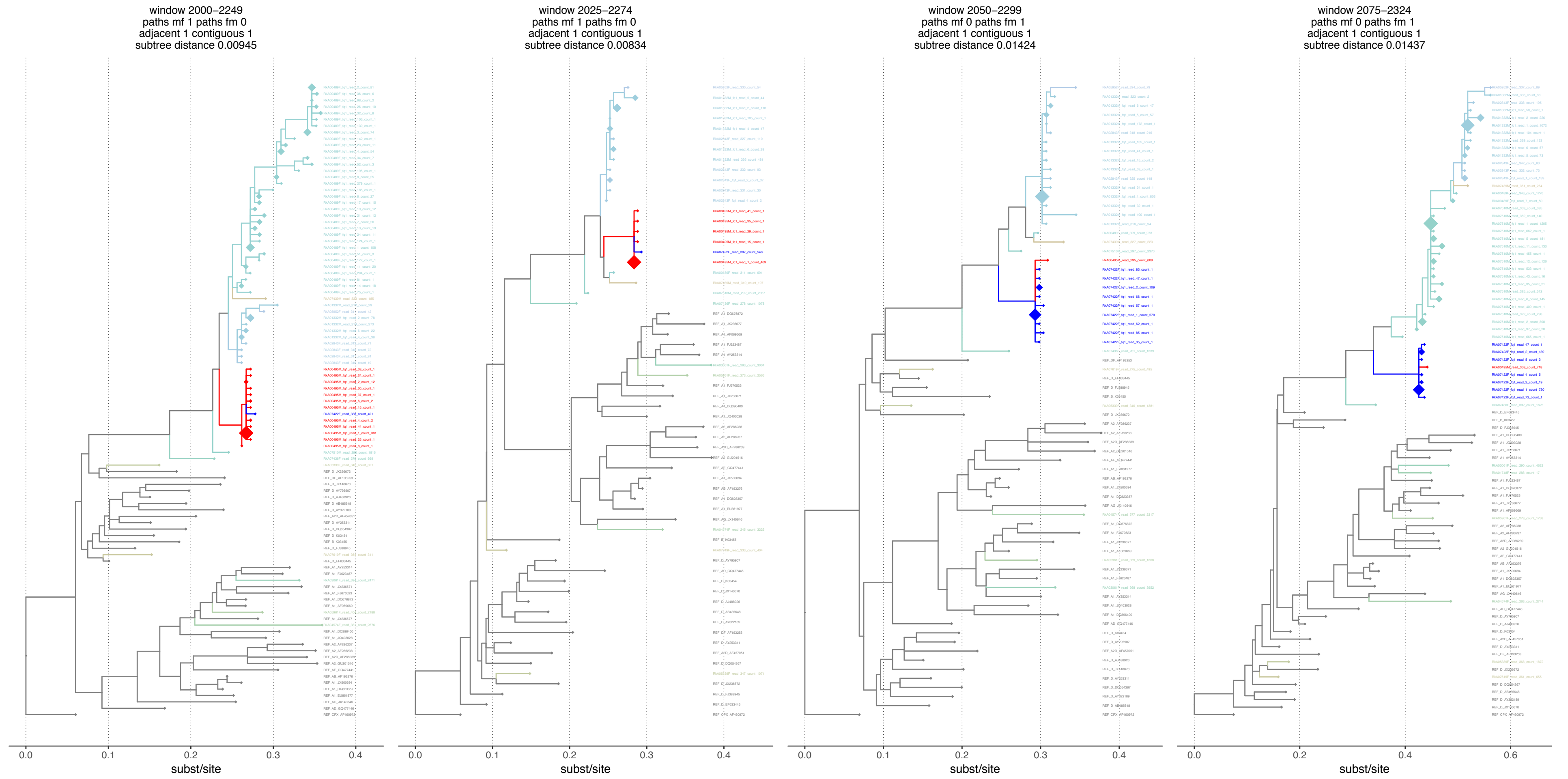
window 2000–2249
paths mf 1 paths fm 0
adjacent 1 contiguous 1
subtree distance 0.00945
window 2025–2274
paths mf 1 paths fm 0
adjacent 1 contiguous 1
subtree distance 0.00834
window 2050–2299
paths mf 0 paths fm 1
adjacent 1 contiguous 1
subtree distance 0.01424
window 2075–2324
paths mf 0 paths fm 1
adjacent 1 contiguous 1
subtree distance 0.01437
0.0
0.1
0.2
0.3
0.4
subst/site

Deep sequence phylogenies
of male-female pair RkA02808M, RkA00505F (run 17)
for whom the phylogenetically inferred direction of transmission was inconsistent with clinical data.

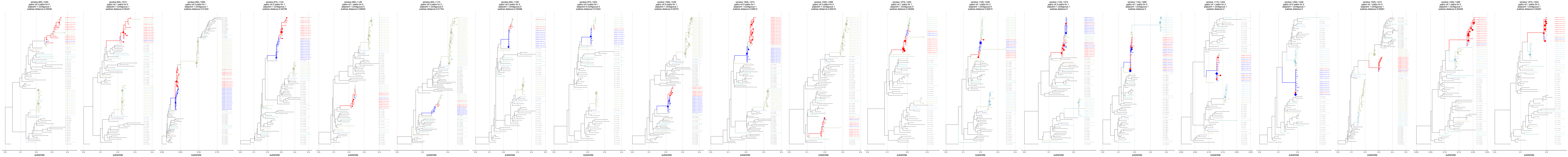
window 800–1049
paths mf 0 paths fm 0
adjacent 1 contiguous 1
subtree distance 0.03548
window 825–1074
paths mf 1 paths fm 0
adjacent 1 contiguous 1
subtree distance 0.00441
window 850–1099
paths mf 0 paths fm 1
adjacent 1 contiguous 1
subtree distance 0.01545
window 875–1124
paths mf 0 paths fm 1
adjacent 1 contiguous 1
subtree distance 0.00709
window 900–1149
paths mf 1 paths fm 0
adjacent 1 contiguous 0
subtree distance 0.00633
window 925–1174
paths mf 0 paths fm 3
adjacent 1 contiguous 0
subtree distance 0.01754
window 950–1199
paths mf 0 paths fm 2
adjacent 1 contiguous 0
subtree distance 0.00596
window 975–1224
paths mf 0 paths fm 1
adjacent 0 contiguous 0
subtree distance 0.01054
window 1000–1249
paths mf 0 paths fm 1
adjacent 1 contiguous 1
subtree distance 0.00791
window 1025–1274
paths mf 0 paths fm 1
adjacent 1 contiguous 1
subtree distance 0.00813
window 1050–1299
paths mf 2 paths fm 0
adjacent 1 contiguous 1
subtree distance 0.00519
window 1075–1324
paths mf 1 paths fm 1
adjacent 1 contiguous 0
subtree distance 0.00265
window 1100–1349
paths mf 1 paths fm 2
adjacent 1 contiguous 0
subtree distance 0.00274
window 1125–1374
paths mf 3 paths fm 1
adjacent 1 contiguous 1
subtree distance 0
window 1150–1399
paths mf 1 paths fm 5
adjacent 1 contiguous 1
subtree distance 0
window 1175–1424
paths mf 1 paths fm 3
adjacent 1 contiguous 1
subtree distance 0
window 1200–1449
paths mf 0 paths fm 2
adjacent 1 contiguous 1
subtree distance 0
window 1225–1474
paths mf 1 paths fm 0
adjacent 1 contiguous 1
subtree distance 0.00391
window 1250–1499
paths mf 1 paths fm 0
adjacent 1 contiguous 1
subtree distance 0.00458
window 1275–1524
paths mf 1 paths fm 0
adjacent 1 contiguous 1
subtree distance 0.00481

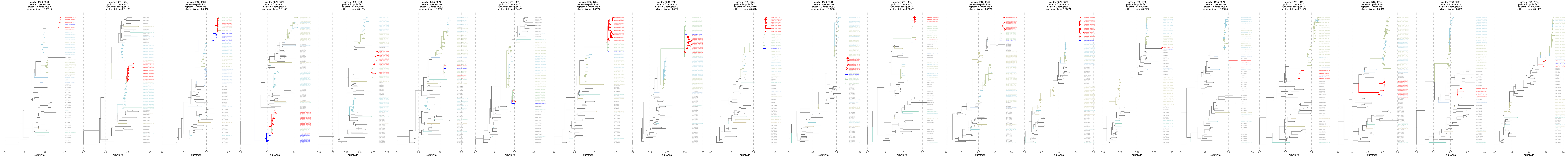

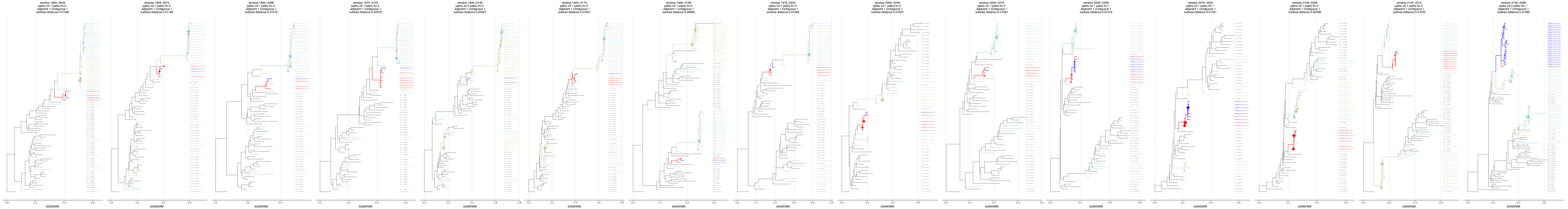
window 1800–2049
paths mf 1 paths fm 0
adjacent 1 contiguous 1
subtree distance 0.01348
window 1825–2074
paths mf 1 paths fm 0
adjacent 1 contiguous 1
subtree distance 0.01166
window 1850–2099
paths mf 1 paths fm 0
adjacent 1 contiguous 1
subtree distance 0.01619
window 1875–2124
paths mf 1 paths fm 0
adjacent 1 contiguous 1
subtree distance 0.02079
window 1900–2149
paths mf 0 paths fm 0
adjacent 1 contiguous 1
subtree distance 0.02427
window 1925–2174
paths mf 1 paths fm 0
adjacent 1 contiguous 1
subtree distance 0.01641
window 1950–2199
paths mf 1 paths fm 0
adjacent 1 contiguous 1
subtree distance 0.00894
window 1975–2224
paths mf 1 paths fm 0
adjacent 1 contiguous 1
subtree distance 0.01484
window 2000–2249
paths mf 1 paths fm 0
adjacent 1 contiguous 1
subtree distance 0.01007
window 2025–2274
paths mf 1 paths fm 0
adjacent 1 contiguous 1
subtree distance 0.01281
window 2050–2299
paths mf 1 paths fm 1
adjacent 1 contiguous 1
subtree distance 0.01316
window 2075–2324
paths mf 1 paths fm 1
adjacent 1 contiguous 1
subtree distance 0.01351
window 2100–2349
paths mf 1 paths fm 0
adjacent 1 contiguous 1
subtree distance 0.00398
window 2125–2374
paths mf 1 paths fm 0
adjacent 1 contiguous 1
subtree distance 0.01025
window 2150–2399
paths mf 0 paths fm 1
adjacent 1 contiguous 1
subtree distance 0.01395
subst/site

Deep sequence phylogenies
of male-female pair RkA06493M, RkA00425F (run 34)
for whom the phylogenetically inferred direction of transmission was inconsistent with clinical data.

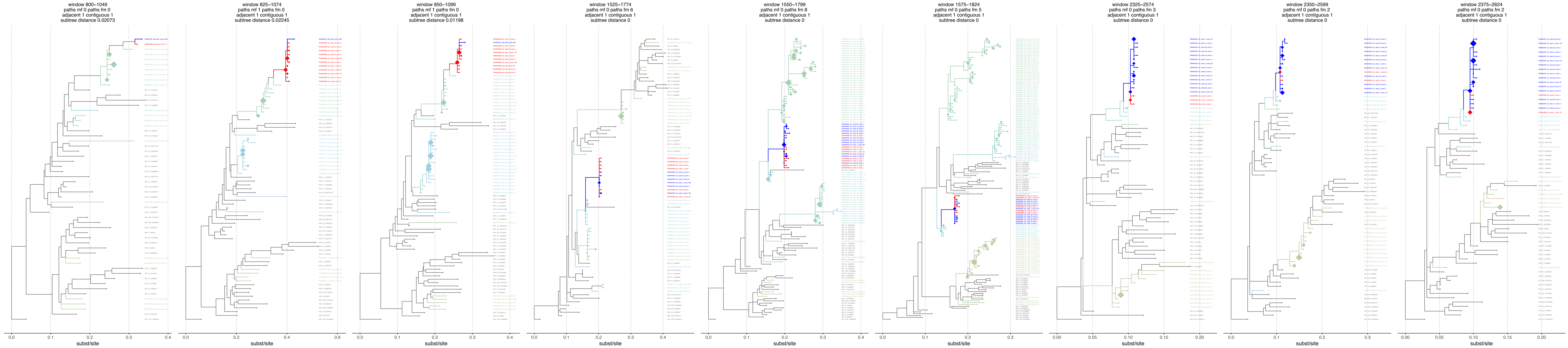
window 800–1049
paths mf 0 paths fm 0
adjacent 1 contiguous 0
subtree distance 0.02073
window 825–1074
paths mf 1 paths fm 0
adjacent 1 contiguous 1
subtree distance 0.02245
window 850–1099
paths mf 0 paths fm 0
adjacent 1 contiguous 1
subtree distance 0.01198
window 1525–1774
paths mf 0 paths fm 6
adjacent 1 contiguous 1
subtree distance 0
window 1550–1799
paths mf 0 paths fm 8
adjacent 1 contiguous 1
subtree distance 0
window 1575–1824
paths mf 0 paths fm 5
adjacent 1 contiguous 1
subtree distance 0
window 2325–2574
paths mf 0 paths fm 3
adjacent 1 contiguous 1
subtree distance 0
window 2350–2599
paths mf 0 paths fm 2
adjacent 1 contiguous 1
subtree distance 0
window 2375–2624
paths mf 0 paths fm 2
adjacent 1 contiguous 1
subtree distance 0
subst/site
subst/site
subst/site
subst/site
subst/site
subst/site
subst/site
subst/site
subst/site

Deep sequence phylogenies
of male-female pair RkA02803M, RkA00868F (run 42)
for whom the phylogenetically inferred direction of transmission was inconsistent with clinical data.

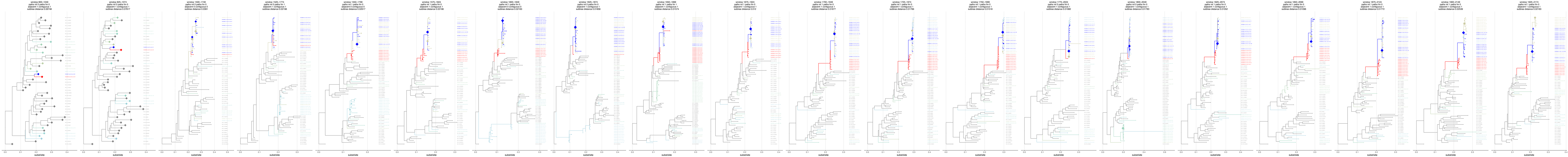

window 800–1049
paths mf 0 paths fm 0
adjacent 1 contiguous 1
subtree distance 0.06748
window 825–1074
paths mf 0 paths fm 0
adjacent 1 contiguous 1
subtree distance 0.07817
window 1500–1749
paths mf 0 paths fm 0
adjacent 0 contiguous 0
subtree distance 0.0391
window 1525–1774
paths mf 0 paths fm 1
adjacent 1 contiguous 1
subtree distance 0.02196
window 1550–1799
paths mf 1 paths fm 0
adjacent 0 contiguous 0
subtree distance 0.02917
window 1575–1824
paths mf 1 paths fm 0
adjacent 1 contiguous 1
subtree distance 0.02196
window 1600–1849
paths mf 1 paths fm 0
adjacent 0 contiguous 0
subtree distance 0.02163
window 1625–1874
paths mf 0 paths fm 0
adjacent 1 contiguous 1
subtree distance 0.01856
window 1650–1899
paths mf 1 paths fm 1
adjacent 1 contiguous 1
subtree distance 0.01574
window 1675–1924
paths mf 1 paths fm 0
adjacent 1 contiguous 1
subtree distance 0.012
window 1700–1949
paths mf 1 paths fm 0
adjacent 1 contiguous 1
subtree distance 0.01677
window 1725–1974
paths mf 1 paths fm 0
adjacent 1 contiguous 1
subtree distance 0.01766
window 1750–1999
paths mf 1 paths fm 0
adjacent 1 contiguous 1
subtree distance 0.01518
window 1775–2024
paths mf 0 paths fm 0
adjacent 1 contiguous 1
subtree distance 0.01982
window 1800–2049
paths mf 0 paths fm 0
adjacent 1 contiguous 1
subtree distance 0.01783
window 1825–2074
paths mf 1 paths fm 0
adjacent 1 contiguous 1
subtree distance 0.01052
window 1850–2099
paths mf 1 paths fm 0
adjacent 1 contiguous 1
subtree distance 0.01096
window 1875–2124
paths mf 1 paths fm 0
adjacent 1 contiguous 1
subtree distance 0.01772
window 1900–2149
paths mf 1 paths fm 0
adjacent 1 contiguous 1
subtree distance 0.02539
window 1925–2174
paths mf 1 paths fm 0
adjacent 1 contiguous 1
subtree distance 0.02163

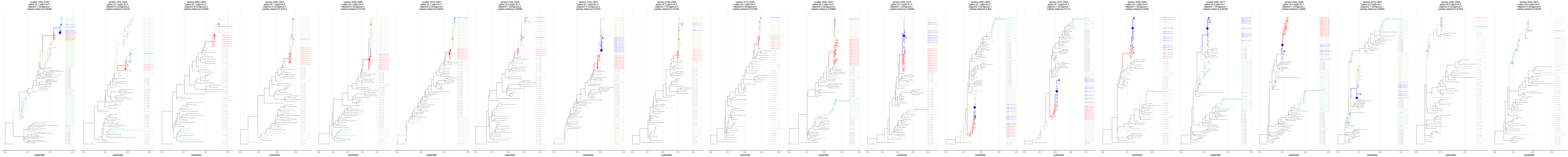

window 1950–2199
paths mf 1 paths fm 0
adjacent 1 contiguous 1
subtree distance 0.02266
window 1975–2224
paths mf 1 paths fm 0
adjacent 0 contiguous 0
subtree distance 0.02104
window 2000–2249
paths mf 1 paths fm 0
adjacent 0 contiguous 0
subtree distance 0.02393
window 2025–2274
paths mf 1 paths fm 0
adjacent 0 contiguous 0
subtree distance 0.01914
window 2050–2299
paths mf 1 paths fm 0
adjacent 0 contiguous 0
subtree distance 0.01454
window 2075–2324
paths mf 1 paths fm 0
adjacent 0 contiguous 0
subtree distance 0.04551
window 2100–2349
paths mf 2 paths fm 0
adjacent 1 contiguous 0
subtree distance 0.02698
window 2125–2374
paths mf 1 paths fm 0
adjacent 0 contiguous 0
subtree distance 0.01975
window 2150–2399
paths mf 2 paths fm 0
adjacent 1 contiguous 0
subtree distance 0.01249
window 2175–2424
paths mf 1 paths fm 0
adjacent 0 contiguous 0
subtree distance 0.01208
window 2200–2449
paths mf 1 paths fm 0
adjacent 0 contiguous 0
subtree distance 0.00856
window 2225–2474
paths mf 1 paths fm 0
adjacent 1 contiguous 1
subtree distance 0.00958
window 2250–2499
paths mf 1 paths fm 0
adjacent 1 contiguous 1
subtree distance 0.005
window 2275–2524
paths mf 1 paths fm 0
adjacent 1 contiguous 1
subtree distance 0.0113
window 2300–2549
paths mf 1 paths fm 0
adjacent 1 contiguous 1
subtree distance 0.0059
window 2325–2574
paths mf 0 paths fm 1
adjacent 1 contiguous 1
subtree distance 0.00709
window 2350–2599
paths mf 0 paths fm 1
adjacent 1 contiguous 1
subtree distance 0.00681
window 2375–2624
paths mf 0 paths fm 1
adjacent 1 contiguous 1
subtree distance 0.00825
window 2400–2649
paths mf 0 paths fm 0
adjacent 0 contiguous 0
subtree distance 0.01924
window 2425–2674
paths mf 0 paths fm 0
adjacent 0 contiguous 0
subtree distance 0.06435
subst/site

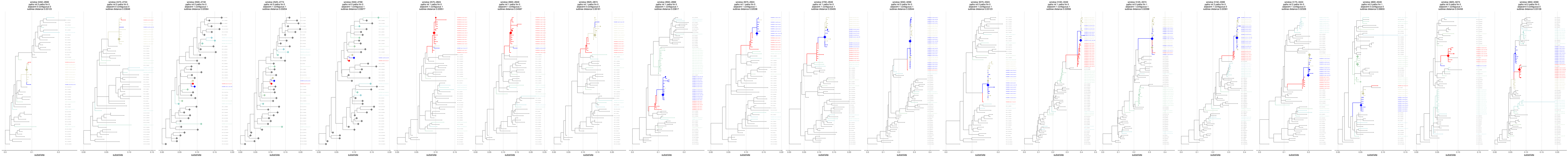
window 2450–2699
paths mf 0 paths fm 0
adjacent 0 contiguous 0
subtree distance 0.05123
window 2475–2724
paths mf 0 paths fm 0
adjacent 0 contiguous 0
subtree distance 0.03946
window 2500–2749
paths mf 0 paths fm 0
adjacent 1 contiguous 1
subtree distance 0.02564
window 2525–2774
paths mf 0 paths fm 0
adjacent 1 contiguous 1
subtree distance 0.03204
window 2550–2799
paths mf 0 paths fm 0
adjacent 1 contiguous 1
subtree distance 0.03977
window 2575–2824
paths mf 1 paths fm 0
adjacent 1 contiguous 1
subtree distance 0.02951
window 2600–2849
paths mf 1 paths fm 0
adjacent 1 contiguous 1
subtree distance 0.04587
window 2625–2874
paths mf 1 paths fm 0
adjacent 0 contiguous 0
subtree distance 0.06181
window 2650–2899
paths mf 1 paths fm 0
adjacent 1 contiguous 1
subtree distance 0.03817
window 2675–2924
paths mf 1 paths fm 0
adjacent 1 contiguous 1
subtree distance 0.02038
window 2700–2949
paths mf 1 paths fm 0
adjacent 1 contiguous 1
subtree distance 0.03405
window 3050–3299
paths mf 0 paths fm 0
adjacent 1 contiguous 1
subtree distance 0.02644
window 3075–3324
paths mf 0 paths fm 0
adjacent 1 contiguous 1
subtree distance 0.03123
window 3100–3349
paths mf 1 paths fm 0
adjacent 1 contiguous 1
subtree distance 0.02956
window 3125–3374
paths mf 0 paths fm 1
adjacent 1 contiguous 1
subtree distance 0.02268
window 3150–3399
paths mf 2 paths fm 0
adjacent 1 contiguous 0
subtree distance 0.02391
window 3175–3424
paths mf 2 paths fm 0
adjacent 1 contiguous 0
subtree distance 0.02326
window 4800–5049
paths mf 0 paths fm 1
adjacent 0 contiguous 0
subtree distance 0.06833
window 4825–5074
paths mf 0 paths fm 0
adjacent 1 contiguous 1
subtree distance 0.04456
window 4850–5099
paths mf 0 paths fm 0
adjacent 0 contiguous 0
subtree distance 0.03128

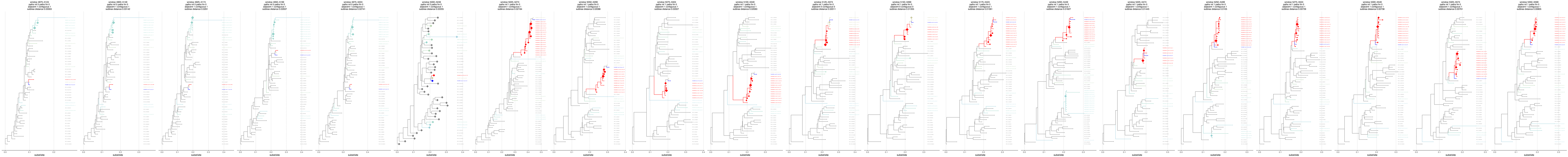
window 4875–5124
paths mf 0 paths fm 0
adjacent 1 contiguous 1
subtree distance 0.03303
window 4900–5149
paths mf 0 paths fm 0
adjacent 1 contiguous 1
subtree distance 0.03142
window 4925–5174
paths mf 0 paths fm 0
adjacent 1 contiguous 1
subtree distance 0.0291
window 4950–5199
paths mf 0 paths fm 0
adjacent 1 contiguous 1
subtree distance 0.02937
window 4975–5224
paths mf 0 paths fm 0
adjacent 1 contiguous 1
subtree distance 0.02741
window 5000–5249
paths mf 0 paths fm 0
adjacent 1 contiguous 1
subtree distance 0.02205
window 5025–5274
paths mf 1 paths fm 0
adjacent 1 contiguous 1
subtree distance 0.02194
window 5050–5299
paths mf 1 paths fm 0
adjacent 1 contiguous 1
subtree distance 0.02088
window 5075–5324
paths mf 1 paths fm 0
adjacent 1 contiguous 1
subtree distance 0.02007
window 5100–5349
paths mf 1 paths fm 0
adjacent 1 contiguous 1
subtree distance 0.02063
window 5125–5374
paths mf 1 paths fm 0
adjacent 0 contiguous 0
subtree distance 0.02011
window 5150–5399
paths mf 1 paths fm 0
adjacent 1 contiguous 1
subtree distance 0.01355
window 5175–5424
paths mf 1 paths fm 0
adjacent 1 contiguous 1
subtree distance 0.01361
window 5200–5449
paths mf 1 paths fm 0
adjacent 1 contiguous 1
subtree distance 0.01827
window 5225–5474
paths mf 1 paths fm 0
adjacent 1 contiguous 1
subtree distance 0.01445
window 5250–5499
paths mf 1 paths fm 0
adjacent 1 contiguous 1
subtree distance 0.0105
window 5275–5524
paths mf 1 paths fm 0
adjacent 1 contiguous 1
subtree distance 0.00729
window 5300–5549
paths mf 1 paths fm 0
adjacent 1 contiguous 1
subtree distance 0.00738
window 5325–5574
paths mf 1 paths fm 0
adjacent 1 contiguous 1
subtree distance 0.00761
window 5350–5599
paths mf 1 paths fm 0
adjacent 1 contiguous 1
subtree distance 0.00805
subst/site

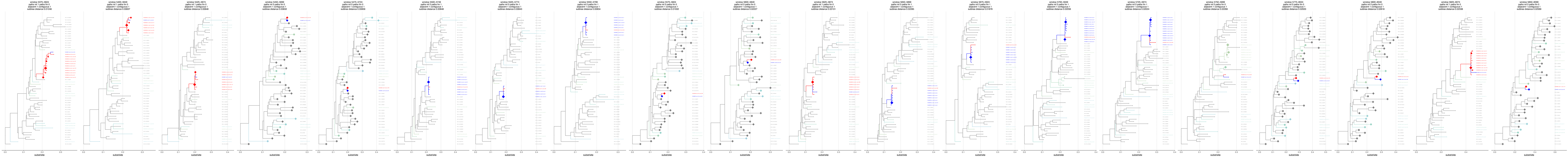
window 5375–5624
paths mf 1 paths fm 0
adjacent 1 contiguous 1
subtree distance 0.01246
window 5400–5649
paths mf 1 paths fm 0
adjacent 1 contiguous 1
subtree distance 0.00837
window 5425–5674
paths mf 1 paths fm 0
adjacent 1 contiguous 1
subtree distance 0.00848
window 5450–5699
paths mf 0 paths fm 0
adjacent 1 contiguous 1
subtree distance 0.00832
window 5475–5724
paths mf 0 paths fm 0
adjacent 1 contiguous 1
subtree distance 0.00856
window 5500–5749
paths mf 0 paths fm 1
adjacent 1 contiguous 1
subtree distance 0.00848
window 5525–5774
paths mf 0 paths fm 1
adjacent 1 contiguous 1
subtree distance 0.00843
window 5550–5799
paths mf 0 paths fm 1
adjacent 1 contiguous 1
subtree distance 0.00834
window 5575–5824
paths mf 0 paths fm 0
adjacent 1 contiguous 1
subtree distance 0.01932
window 5600–5849
paths mf 0 paths fm 0
adjacent 1 contiguous 1
subtree distance 0.02227
window 5625–5874
paths mf 1 paths fm 0
adjacent 1 contiguous 1
subtree distance 0.02101
window 5650–5899
paths mf 0 paths fm 1
adjacent 1 contiguous 1
subtree distance 0.02285
window 5675–5924
paths mf 0 paths fm 1
adjacent 1 contiguous 1
subtree distance 0.01878
window 5700–5949
paths mf 0 paths fm 1
adjacent 1 contiguous 1
subtree distance 0.02056
window 5725–5974
paths mf 0 paths fm 1
adjacent 1 contiguous 1
subtree distance 0.02024
window 5750–5999
paths mf 0 paths fm 0
adjacent 1 contiguous 1
subtree distance 0.02269
window 5775–6024
paths mf 0 paths fm 0
adjacent 1 contiguous 1
subtree distance 0.02933
window 5800–6049
paths mf 0 paths fm 0
adjacent 1 contiguous 1
subtree distance 0.02818
window 5825–6074
paths mf 1 paths fm 0
adjacent 1 contiguous 1
subtree distance 0.02338
window 5850–6099
paths mf 0 paths fm 0
adjacent 1 contiguous 1
subtree distance 0.02569

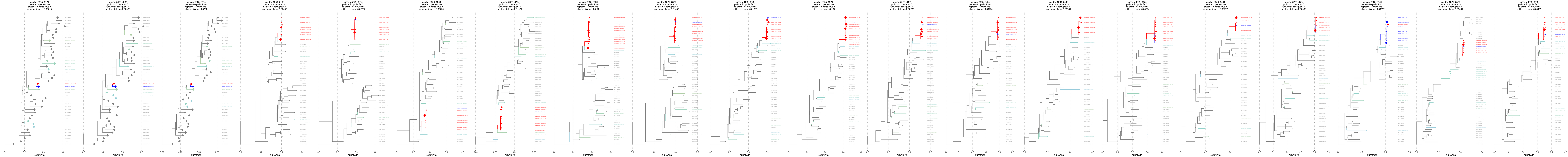

window 5875–6124
paths mf 0 paths fm 0
adjacent 1 contiguous 1
subtree distance 0.02718
window 5900–6149
paths mf 0 paths fm 0
adjacent 1 contiguous 1
subtree distance 0.02468
window 5925–6174
paths mf 0 paths fm 0
adjacent 1 contiguous 1
subtree distance 0.02621
window 5950–6199
paths mf 1 paths fm 0
adjacent 1 contiguous 1
subtree distance 0.02497
window 5975–6224
paths mf 1 paths fm 0
adjacent 1 contiguous 1
subtree distance 0.02697
window 6000–6249
paths mf 1 paths fm 0
adjacent 1 contiguous 1
subtree distance 0.02759
window 6025–6274
paths mf 1 paths fm 0
adjacent 1 contiguous 1
subtree distance 0.01921
window 6050–6299
paths mf 1 paths fm 0
adjacent 1 contiguous 1
subtree distance 0.01721
window 6075–6324
paths mf 1 paths fm 0
adjacent 1 contiguous 1
subtree distance 0.01458
window 6100–6349
paths mf 1 paths fm 0
adjacent 1 contiguous 1
subtree distance 0.01069
window 6125–6374
paths mf 1 paths fm 0
adjacent 1 contiguous 1
subtree distance 0.0107
window 6150–6399
paths mf 1 paths fm 0
adjacent 1 contiguous 1
subtree distance 0.00785
window 6175–6424
paths mf 1 paths fm 0
adjacent 1 contiguous 1
subtree distance 0.00774
window 6200–6449
paths mf 1 paths fm 0
adjacent 1 contiguous 1
subtree distance 0.00781
window 6225–6474
paths mf 1 paths fm 0
adjacent 1 contiguous 1
subtree distance 0.00774
window 6250–6499
paths mf 1 paths fm 0
adjacent 1 contiguous 1
subtree distance 0.00277
window 6275–6524
paths mf 1 paths fm 0
adjacent 1 contiguous 1
subtree distance 0.00493
window 6300–6549
paths mf 0 paths fm 1
adjacent 1 contiguous 1
subtree distance 0.00597
window 6325–6574
paths mf 1 paths fm 0
adjacent 1 contiguous 1
subtree distance 0.00461
window 6350–6599
paths mf 1 paths fm 0
adjacent 1 contiguous 1
subtree distance 0.00436

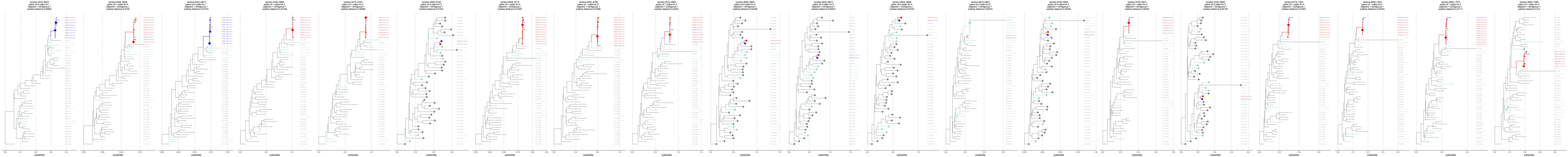
window 6375–6624
paths mf 0 paths fm 1
adjacent 1 contiguous 1
subtree distance 0.00697
window 6400–6649
paths mf 1 paths fm 0
adjacent 1 contiguous 1
subtree distance 0.0103
window 6425–6674
paths mf 0 paths fm 1
adjacent 1 contiguous 1
subtree distance 0.01275
window 6450–6699
paths mf 1 paths fm 0
adjacent 1 contiguous 1
subtree distance 0.01549
window 6475–6724
paths mf 1 paths fm 0
adjacent 1 contiguous 1
subtree distance 0.00859
window 6500–6749
paths mf 0 paths fm 0
adjacent 1 contiguous 1
subtree distance 0.01051
window 6525–6774
paths mf 1 paths fm 0
adjacent 1 contiguous 1
subtree distance 0.01067
window 6550–6799
paths mf 1 paths fm 0
adjacent 1 contiguous 1
subtree distance 0.01808
window 6575–6824
paths mf 1 paths fm 0
adjacent 1 contiguous 1
subtree distance 0.02561
window 6600–6849
paths mf 0 paths fm 0
adjacent 1 contiguous 1
subtree distance 0.02448
window 6625–6874
paths mf 0 paths fm 0
adjacent 1 contiguous 1
subtree distance 0.03031
window 6650–6899
paths mf 0 paths fm 0
adjacent 1 contiguous 1
subtree distance 0.03753
window 6675–6924
paths mf 0 paths fm 0
adjacent 1 contiguous 1
subtree distance 0.02079
window 6700–6949
paths mf 0 paths fm 0
adjacent 1 contiguous 1
subtree distance 0.02302
window 6725–6974
paths mf 1 paths fm 0
adjacent 1 contiguous 1
subtree distance 0.02204
window 6750–6999
paths mf 0 paths fm 0
adjacent 1 contiguous 1
subtree distance 0.02156
window 6775–7024
paths mf 1 paths fm 0
adjacent 1 contiguous 1
subtree distance 0.01801
window 6800–7049
paths mf 1 paths fm 0
adjacent 1 contiguous 1
subtree distance 0.00764
window 6825–7074
paths mf 1 paths fm 0
adjacent 1 contiguous 1
subtree distance 0.01711
window 6850–7099
paths mf 1 paths fm 0
adjacent 1 contiguous 1
subtree distance 0.014

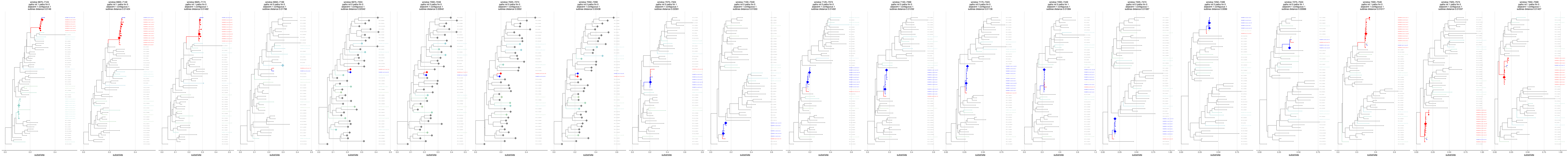
window 6875–7124
paths mf 1 paths fm 0
adjacent 1 contiguous 1
subtree distance 0.0146
window 6900–7149
paths mf 1 paths fm 0
adjacent 1 contiguous 1
subtree distance 0.01424
window 6925–7174
paths mf 1 paths fm 0
adjacent 1 contiguous 1
subtree distance 0.01492
window 6950–7199
paths mf 0 paths fm 0
adjacent 1 contiguous 1
subtree distance 0.01796
window 6975–7224
paths mf 0 paths fm 0
adjacent 1 contiguous 1
subtree distance 0.02342
window 7000–7249
paths mf 0 paths fm 0
adjacent 1 contiguous 1
subtree distance 0.02642
window 7025–7274
paths mf 0 paths fm 0
adjacent 1 contiguous 1
subtree distance 0.0302
window 7050–7299
paths mf 0 paths fm 0
adjacent 1 contiguous 1
subtree distance 0.02438
window 7075–7324
paths mf 0 paths fm 1
adjacent 1 contiguous 1
subtree distance 0.02081
window 7100–7349
paths mf 0 paths fm 0
adjacent 1 contiguous 1
subtree distance 0.01553
window 7125–7374
paths mf 0 paths fm 0
adjacent 1 contiguous 1
subtree distance 0.01402
window 7150–7399
paths mf 0 paths fm 0
adjacent 1 contiguous 1
subtree distance 0.01232
window 7175–7424
paths mf 0 paths fm 0
adjacent 1 contiguous 1
subtree distance 0.01136
window 7200–7449
paths mf 0 paths fm 1
adjacent 1 contiguous 1
subtree distance 0.01084
window 7225–7474
paths mf 0 paths fm 0
adjacent 1 contiguous 1
subtree distance 0.01567
window 7250–7499
paths mf 0 paths fm 0
adjacent 1 contiguous 1
subtree distance 0.0099
window 7275–7524
paths mf 0 paths fm 1
adjacent 1 contiguous 1
subtree distance 0.00999
window 7300–7549
paths mf 1 paths fm 0
adjacent 1 contiguous 1
subtree distance 0.01017
window 7325–7574
paths mf 1 paths fm 0
adjacent 1 contiguous 1
subtree distance 0.01037
window 7350–7599
paths mf 1 paths fm 0
adjacent 1 contiguous 1
subtree distance 0.01407

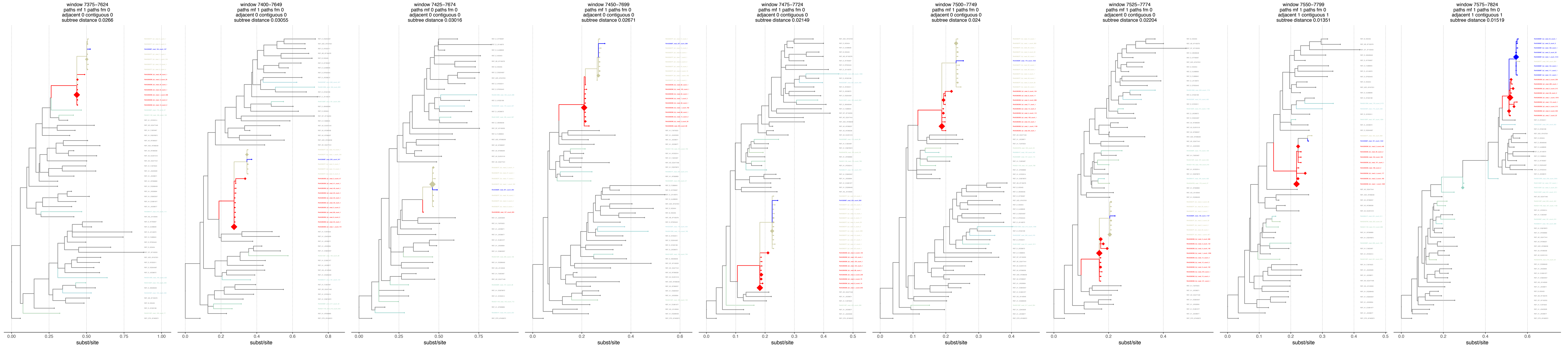
window 7375–7624
paths mf 1 paths fm 0
adjacent 0 contiguous 0
subtree distance 0.0266
subst/site
window 7400–7649
paths mf 1 paths fm 0
adjacent 0 contiguous 0
subtree distance 0.03055
subst/site
window 7425–7674
paths mf 0 paths fm 0
adjacent 0 contiguous 0
subtree distance 0.03016
subst/site
window 7450–7699
paths mf 1 paths fm 0
adjacent 0 contiguous 0
subtree distance 0.02671
subst/site
window 7475–7724
paths mf 1 paths fm 0
adjacent 0 contiguous 0
subtree distance 0.02149
subst/site
window 7500–7749
paths mf 1 paths fm 0
adjacent 0 contiguous 0
subtree distance 0.024
subst/site
window 7525–7774
paths mf 1 paths fm 0
adjacent 0 contiguous 0
subtree distance 0.02204
subst/site
window 7550–7799
paths mf 1 paths fm 0
adjacent 1 contiguous 1
subtree distance 0.01351
subst/site
window 7575–7824
paths mf 1 paths fm 0
adjacent 1 contiguous 1
subtree distance 0.01519
subst/site

Deep sequence phylogenies
of male-female pair RkA02918M, RkA02908F (run 83)
for whom the phylogenetically inferred direction of transmission was inconsistent with clinical data.

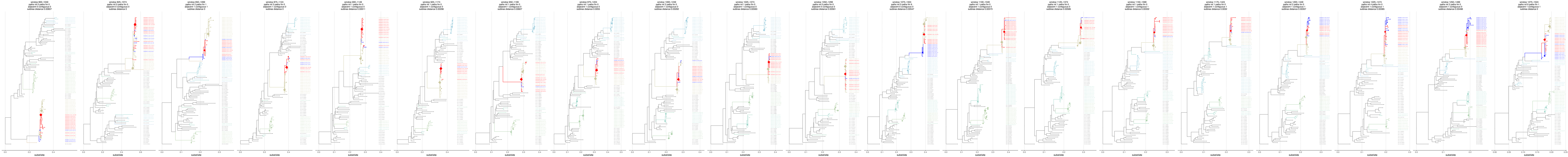
window 800–1049
paths mf 0 paths fm 0
adjacent 0 contiguous 0
subtree distance 0.00607
window 825–1074
paths mf 0 paths fm 0
adjacent 0 contiguous 0
subtree distance 0
window 850–1099
paths mf 2 paths fm 1
adjacent 1 contiguous 1
subtree distance 0
window 875–1124
paths mf 3 paths fm 0
adjacent 1 contiguous 0
subtree distance 0
window 900–1149
paths mf 1 paths fm 0
adjacent 1 contiguous 0
subtree distance 0.00911
window 925–1174
paths mf 1 paths fm 0
adjacent 1 contiguous 0
subtree distance 0.00296
window 950–1199
paths mf 1 paths fm 0
adjacent 1 contiguous 0
subtree distance 0.00827
window 975–1224
paths mf 1 paths fm 0
adjacent 1 contiguous 0
subtree distance 0.0055
window 1000–1249
paths mf 2 paths fm 0
adjacent 1 contiguous 0
subtree distance 0.00262
window 1025–1274
paths mf 1 paths fm 0
adjacent 1 contiguous 0
subtree distance 0.00563
window 1050–1299
paths mf 0 paths fm 0
adjacent 0 contiguous 0
subtree distance 0.00561
window 1075–1324
paths mf 0 paths fm 9
adjacent 0 contiguous 0
subtree distance 0.00547
window 1100–1349
paths mf 1 paths fm 0
adjacent 1 contiguous 0
subtree distance 0.00573
window 1125–1374
paths mf 1 paths fm 0
adjacent 1 contiguous 0
subtree distance 0.00585
window 1150–1399
paths mf 1 paths fm 0
adjacent 1 contiguous 1
subtree distance 0.00332
window 1175–1424
paths mf 1 paths fm 0
adjacent 1 contiguous 1
subtree distance 0.0032
window 1200–1449
paths mf 2 paths fm 0
adjacent 1 contiguous 1
subtree distance 0.00357
window 1225–1474
paths mf 4 paths fm 0
adjacent 1 contiguous 1
subtree distance 0.00385
window 1250–1499
paths mf 2 paths fm 0
adjacent 1 contiguous 0
subtree distance 0.00496
window 1275–1524
paths mf 6 paths fm 1
adjacent 1 contiguous 1
subtree distance 0

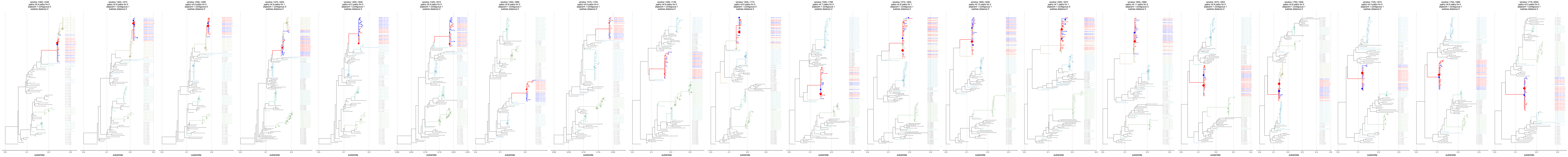
window 1300–1549
paths mf 6 paths fm 0
adjacent 1 contiguous 0
subtree distance 0
window 1325–1574
paths mf 9 paths fm 0
adjacent 1 contiguous 1
subtree distance 0
window 1350–1599
paths mf 6 paths fm 0
adjacent 1 contiguous 0
subtree distance 0
window 1375–1624
paths mf 9 paths fm 1
adjacent 1 contiguous 0
subtree distance 0
window 1400–1649
paths mf 5 paths fm 0
adjacent 1 contiguous 1
subtree distance 0
window 1425–1674
paths mf 6 paths fm 0
adjacent 1 contiguous 1
subtree distance 0
window 1450–1699
paths mf 8 paths fm 0
adjacent 1 contiguous 1
subtree distance 0
window 1475–1724
paths mf 6 paths fm 0
adjacent 1 contiguous 1
subtree distance 0
window 1500–1749
paths mf 8 paths fm 0
adjacent 1 contiguous 1
subtree distance 0
window 1525–1774
paths mf 6 paths fm 0
adjacent 1 contiguous 0
subtree distance 0
window 1550–1799
paths mf 7 paths fm 0
adjacent 1 contiguous 1
subtree distance 0
window 1575–1824
paths mf 8 paths fm 1
adjacent 1 contiguous 1
subtree distance 0
window 1600–1849
paths mf 10 paths fm 2
adjacent 1 contiguous 1
subtree distance 0
window 1625–1874
paths mf 7 paths fm 1
adjacent 1 contiguous 0
subtree distance 0
window 1650–1899
paths mf 11 paths fm 0
adjacent 1 contiguous 0
subtree distance 0
window 1675–1924
paths mf 8 paths fm 0
adjacent 1 contiguous 1
subtree distance 0
window 1700–1949
paths mf 8 paths fm 0
adjacent 1 contiguous 1
subtree distance 0
window 1725–1974
paths mf 4 paths fm 0
adjacent 1 contiguous 1
subtree distance 0
window 1750–1999
paths mf 6 paths fm 0
adjacent 1 contiguous 1
subtree distance 0
window 1775–2024
paths mf 5 paths fm 0
adjacent 1 contiguous 1
subtree distance 0

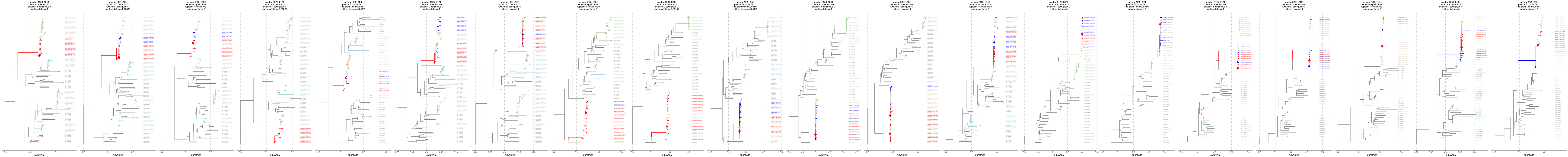
window 1800–2049
paths mf 3 paths fm 1
adjacent 1 contiguous 1
subtree distance 0
window 1825–2074
paths mf 4 paths fm 0
adjacent 1 contiguous 1
subtree distance 0.00305
window 1850–2099
paths mf 2 paths fm 0
adjacent 1 contiguous 1
subtree distance 0
window 1875–2124
paths mf 1 paths fm 0
adjacent 1 contiguous 1
subtree distance 0.00321
window 1900–2149
paths mf 1 paths fm 0
adjacent 1 contiguous 1
subtree distance 0.00339
window 1925–2174
paths mf 2 paths fm 0
adjacent 0 contiguous 0
subtree distance 0
window 1950–2199
paths mf 3 paths fm 0
adjacent 0 contiguous 0
subtree distance 0
window 1975–2224
paths mf 3 paths fm 0
adjacent 0 contiguous 0
subtree distance 0
window 2000–2249
paths mf 1 paths fm 0
adjacent 0 contiguous 0
subtree distance 0.00405
window 2025–2274
paths mf 0 paths fm 0
adjacent 0 contiguous 0
subtree distance 0.00425
window 2050–2299
paths mf 6 paths fm 0
adjacent 1 contiguous 0
subtree distance 0
window 2075–2324
paths mf 10 paths fm 0
adjacent 1 contiguous 0
subtree distance 0
window 2100–2349
paths mf 12 paths fm 1
adjacent 1 contiguous 0
subtree distance 0
window 2125–2374
paths mf 9 paths fm 0
adjacent 1 contiguous 0
subtree distance 0
window 2150–2399
paths mf 5 paths fm 1
adjacent 1 contiguous 0
subtree distance 0
window 2175–2424
paths mf 4 paths fm 0
adjacent 1 contiguous 1
subtree distance 0
window 2200–2449
paths mf 10 paths fm 0
adjacent 1 contiguous 0
subtree distance 0
window 2225–2474
paths mf 6 paths fm 0
adjacent 1 contiguous 1
subtree distance 0
window 2250–2499
paths mf 2 paths fm 2
adjacent 1 contiguous 1
subtree distance 0
window 2275–2524
paths mf 2 paths fm 1
adjacent 1 contiguous 1
subtree distance 0
subst/site

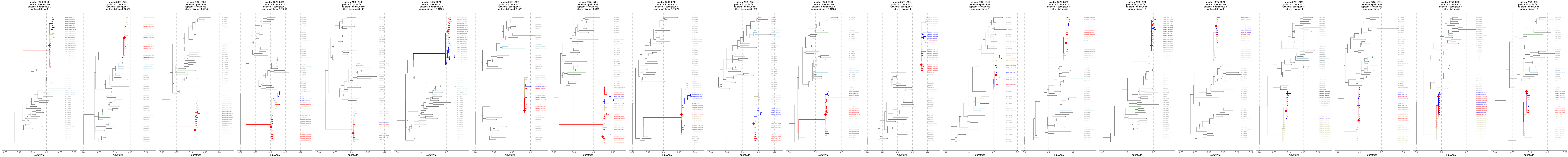
window 2300–2549
paths mf 2 paths fm 0
adjacent 1 contiguous 0
subtree distance 0
window 2325–2574
paths mf 1 paths fm 0
adjacent 1 contiguous 1
subtree distance 0.00574
window 2350–2599
paths mf 1 paths fm 0
adjacent 1 contiguous 1
subtree distance 0.01246
window 2375–2624
paths mf 2 paths fm 0
adjacent 1 contiguous 0
subtree distance 0.01296
window 2400–2649
paths mf 1 paths fm 0
adjacent 1 contiguous 0
subtree distance 0.01401
window 2425–2674
paths mf 0 paths fm 1
adjacent 1 contiguous 1
subtree distance 0.01624
window 2450–2699
paths mf 2 paths fm 0
adjacent 1 contiguous 1
subtree distance 0.01528
window 2475–2724
paths mf 2 paths fm 0
adjacent 1 contiguous 1
subtree distance 0.00781
window 2500–2749
paths mf 2 paths fm 0
adjacent 1 contiguous 1
subtree distance 0.01671
window 2525–2774
paths mf 2 paths fm 0
adjacent 1 contiguous 1
subtree distance 0.01622
window 2550–2799
paths mf 2 paths fm 0
adjacent 1 contiguous 1
subtree distance 0.01638
window 2575–2824
paths mf 4 paths fm 0
adjacent 1 contiguous 1
subtree distance 0
window 2600–2849
paths mf 5 paths fm 0
adjacent 1 contiguous 1
subtree distance 0
window 2625–2874
paths mf 3 paths fm 0
adjacent 1 contiguous 1
subtree distance 0
window 2650–2899
paths mf 4 paths fm 0
adjacent 1 contiguous 1
subtree distance 0
window 2675–2924
paths mf 6 paths fm 0
adjacent 1 contiguous 1
subtree distance 0
window 2700–2949
paths mf 5 paths fm 0
adjacent 1 contiguous 1
subtree distance 0
window 2725–2974
paths mf 9 paths fm 0
adjacent 1 contiguous 1
subtree distance 0
window 2750–2999
paths mf 4 paths fm 0
adjacent 1 contiguous 1
subtree distance 0
window 2775–3024
paths mf 5 paths fm 0
adjacent 1 contiguous 1
subtree distance 0

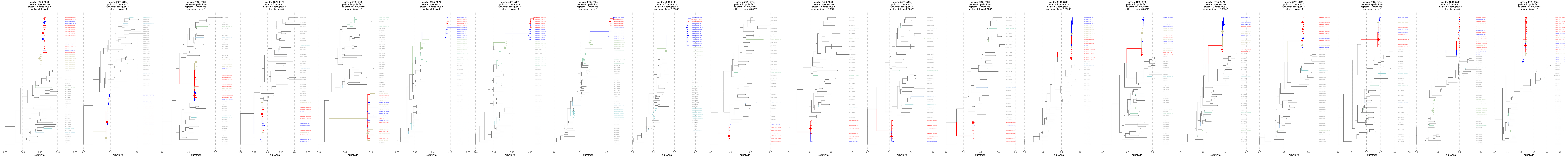
window 2800–3049
paths mf 4 paths fm 0
adjacent 1 contiguous 1
subtree distance 0
window 2825–3074
paths mf 3 paths fm 0
adjacent 1 contiguous 0
subtree distance 0
window 2850–3099
paths mf 4 paths fm 0
adjacent 1 contiguous 1
subtree distance 0
window 2875–3124
paths mf 3 paths fm 1
adjacent 1 contiguous 1
subtree distance 0
window 4800–5049
paths mf 0 paths fm 0
adjacent 0 contiguous 0
subtree distance 0
window 4825–5074
paths mf 2 paths fm 1
adjacent 1 contiguous 1
subtree distance 0
window 4850–5099
paths mf 1 paths fm 1
adjacent 1 contiguous 1
subtree distance 0
window 4875–5124
paths mf 1 paths fm 1
adjacent 1 contiguous 1
subtree distance 0
window 4900–5149
paths mf 0 paths fm 2
adjacent 1 contiguous 1
subtree distance 0.00537
window 5375–5624
paths mf 1 paths fm 0
adjacent 1 contiguous 1
subtree distance 0.00825
window 5400–5649
paths mf 2 paths fm 0
adjacent 1 contiguous 1
subtree distance 0.00791
window 5425–5674
paths mf 1 paths fm 0
adjacent 1 contiguous 1
subtree distance 0.00829
window 5450–5699
paths mf 1 paths fm 0
adjacent 1 contiguous 1
subtree distance 0.0082
window 6100–6349
paths mf 2 paths fm 0
adjacent 0 contiguous 0
subtree distance 0.00262
window 6150–6399
paths mf 2 paths fm 0
adjacent 0 contiguous 0
subtree distance 0.00248
window 6175–6424
paths mf 2 paths fm 0
adjacent 0 contiguous 0
subtree distance 0.00247
window 6200–6449
paths mf 0 paths fm 0
adjacent 0 contiguous 0
subtree distance 0
window 6225–6474
paths mf 3 paths fm 0
adjacent 1 contiguous 1
subtree distance 0
window 6300–6549
paths mf 3 paths fm 1
adjacent 1 contiguous 1
subtree distance 0
window 6325–6574
paths mf 3 paths fm 1
adjacent 1 contiguous 1
subtree distance 0

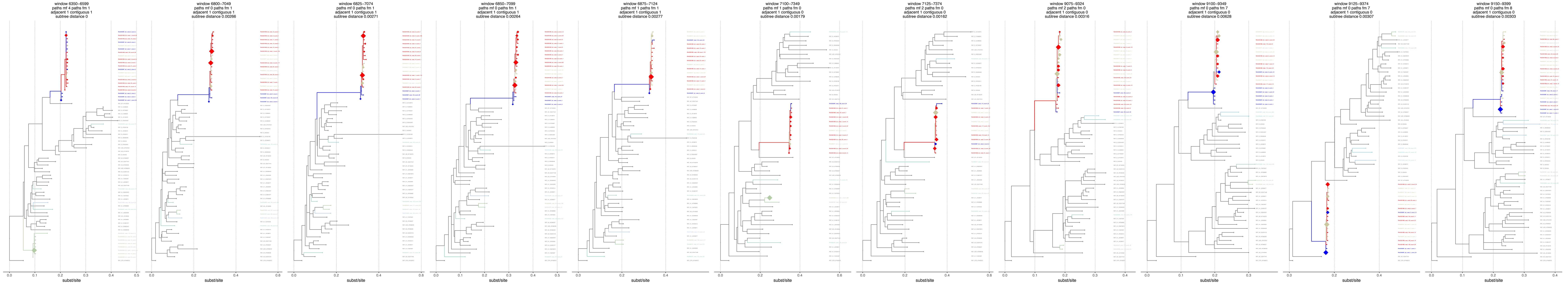

window 6350–6599
paths mf 4 paths fm 1
adjacent 1 contiguous 1
subtree distance 0
window 6800–7049
paths mf 0 paths fm 1
adjacent 1 contiguous 1
subtree distance 0.00266
window 6825–7074
paths mf 0 paths fm 1
adjacent 1 contiguous 1
subtree distance 0.00271
window 6850–7099
paths mf 0 paths fm 1
adjacent 1 contiguous 1
subtree distance 0.00264
window 6875–7124
paths mf 1 paths fm 1
adjacent 1 contiguous 1
subtree distance 0.00277
window 7100–7349
paths mf 1 paths fm 0
adjacent 1 contiguous 0
subtree distance 0.00179
window 7125–7374
paths mf 2 paths fm 0
adjacent 1 contiguous 0
subtree distance 0.00162
window 9075–9324
paths mf 2 paths fm 0
adjacent 1 contiguous 0
subtree distance 0.00316
window 9100–9349
paths mf 0 paths fm 7
adjacent 1 contiguous 0
subtree distance 0.00628
window 9125–9374
paths mf 0 paths fm 7
adjacent 1 contiguous 0
subtree distance 0.00307
window 9150–9399
paths mf 0 paths fm 8
adjacent 1 contiguous 0
subtree distance 0.00303
subst/site

Deep sequence phylogenies
of male-female pair RkA07475M, RkA03573F (run 204)
for whom the phylogenetically inferred direction of transmission was inconsistent with clinical data.

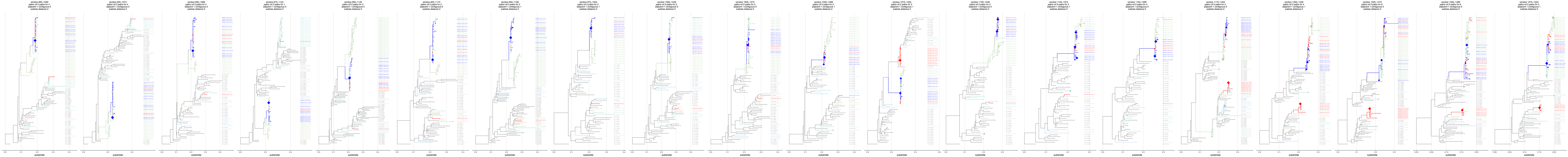
window 800–1049
paths mf 0 paths fm 4
adjacent 1 contiguous 0
subtree distance 0
window 825–1074
paths mf 0 paths fm 4
adjacent 1 contiguous 0
subtree distance 0
window 850–1099
paths mf 0 paths fm 4
adjacent 1 contiguous 0
subtree distance 0
window 875–1124
paths mf 0 paths fm 1
adjacent 1 contiguous 0
subtree distance 0
window 900–1149
paths mf 0 paths fm 3
adjacent 1 contiguous 0
subtree distance 0
window 925–1174
paths mf 0 paths fm 2
adjacent 1 contiguous 0
subtree distance 0
window 950–1199
paths mf 0 paths fm 2
adjacent 1 contiguous 1
subtree distance 0
window 975–1224
paths mf 0 paths fm 3
adjacent 1 contiguous 1
subtree distance 0
window 1000–1249
paths mf 0 paths fm 3
adjacent 1 contiguous 0
subtree distance 0
window 1025–1274
paths mf 0 paths fm 3
adjacent 1 contiguous 0
subtree distance 0
window 1050–1299
paths mf 0 paths fm 3
adjacent 1 contiguous 1
subtree distance 0
window 1075–1324
paths mf 0 paths fm 3
adjacent 1 contiguous 1
subtree distance 0
window 1100–1349
paths mf 0 paths fm 2
adjacent 1 contiguous 0
subtree distance 0
window 1125–1374
paths mf 0 paths fm 3
adjacent 1 contiguous 1
subtree distance 0
window 1150–1399
paths mf 0 paths fm 4
adjacent 1 contiguous 0
subtree distance 0
window 1175–1424
paths mf 0 paths fm 3
adjacent 1 contiguous 0
subtree distance 0
window 1200–1449
paths mf 0 paths fm 4
adjacent 1 contiguous 1
subtree distance 0
window 1225–1474
paths mf 0 paths fm 6
adjacent 1 contiguous 0
subtree distance 0
window 1250–1499
paths mf 0 paths fm 8
adjacent 1 contiguous 0
subtree distance 0
window 1275–1524
paths mf 0 paths fm 5
adjacent 1 contiguous 1
subtree distance 0

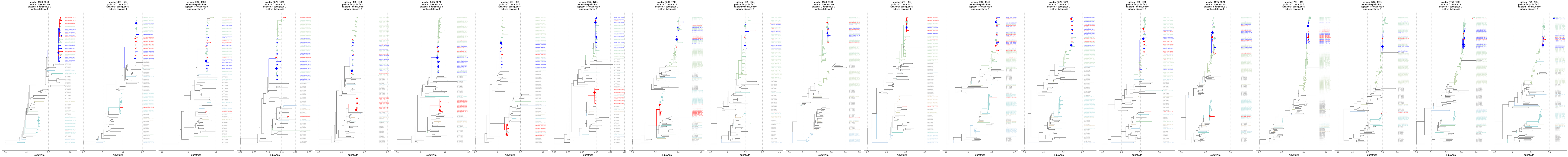
window 1300–1549
paths mf 0 paths fm 6
adjacent 1 contiguous 0
subtree distance 0
subst/site
window 1325–1574
paths mf 0 paths fm 6
adjacent 1 contiguous 0
subtree distance 0
subst/site
window 1350–1599
paths mf 0 paths fm 6
adjacent 1 contiguous 0
subtree distance 0
subst/site
window 1375–1624
paths mf 0 paths fm 2
adjacent 1 contiguous 0
subtree distance 0
subst/site
window 1400–1649
paths mf 0 paths fm 4
adjacent 1 contiguous 1
subtree distance 0
subst/site
window 1425–1674
paths mf 0 paths fm 3
adjacent 1 contiguous 1
subtree distance 0
subst/site
window 1450–1699
paths mf 0 paths fm 3
adjacent 1 contiguous 1
subtree distance 0
subst/site
window 1475–1724
paths mf 0 paths fm 1
adjacent 1 contiguous 0
subtree distance 0
subst/site
window 1500–1749
paths mf 0 paths fm 5
adjacent 1 contiguous 0
subtree distance 0
subst/site
window 1525–1774
paths mf 0 paths fm 0
adjacent 0 contiguous 0
subtree distance 0
subst/site
window 1550–1799
paths mf 0 paths fm 0
adjacent 0 contiguous 0
subtree distance 0
subst/site
window 1575–1824
paths mf 0 paths fm 0
adjacent 0 contiguous 0
subtree distance 0
subst/site
window 1600–1849
paths mf 0 paths fm 0
adjacent 1 contiguous 0
subtree distance 0
subst/site
window 1625–1874
paths mf 0 paths fm 7
adjacent 1 contiguous 0
subtree distance 0
subst/site
window 1650–1899
paths mf 0 paths fm 7
adjacent 1 contiguous 0
subtree distance 0
subst/site
window 1675–1924
paths mf 1 paths fm 4
adjacent 1 contiguous 0
subtree distance 0
subst/site
window 1700–1949
paths mf 0 paths fm 8
adjacent 1 contiguous 0
subtree distance 0
subst/site
window 1725–1974
paths mf 0 paths fm 3
adjacent 1 contiguous 0
subtree distance 0
subst/site
window 1750–1999
paths mf 0 paths fm 4
adjacent 1 contiguous 0
subtree distance 0
subst/site
window 1775–2024
paths mf 0 paths fm 3
adjacent 1 contiguous 0
subtree distance 0
subst/site

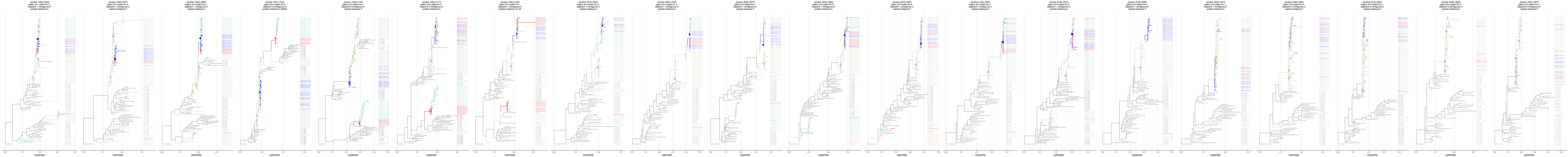

window 1800–2049
paths mf 1 paths fm 3
adjacent 1 contiguous 0
subtree distance 0
window 1825–2074
paths mf 0 paths fm 3
adjacent 1 contiguous 0
subtree distance 0
window 1850–2099
paths mf 0 paths fm 5
adjacent 1 contiguous 0
subtree distance 0
window 1875–2124
paths mf 0 paths fm 0
adjacent 0 contiguous 0
subtree distance 0.00355
window 1900–2149
paths mf 0 paths fm 0
adjacent 0 contiguous 0
subtree distance 0
window 1925–2174
paths mf 0 paths fm 0
adjacent 0 contiguous 0
subtree distance 0
window 1950–2199
paths mf 3 paths fm 1
adjacent 1 contiguous 0
subtree distance 7e−05
window 1975–2224
paths mf 0 paths fm 3
adjacent 1 contiguous 0
subtree distance 0
window 2000–2249
paths mf 0 paths fm 4
adjacent 1 contiguous 0
subtree distance 0
window 2025–2274
paths mf 0 paths fm 4
adjacent 1 contiguous 0
subtree distance 0
window 2050–2299
paths mf 0 paths fm 9
adjacent 1 contiguous 0
subtree distance 0
window 2075–2324
paths mf 0 paths fm 6
adjacent 1 contiguous 0
subtree distance 0
window 2100–2349
paths mf 0 paths fm 3
adjacent 1 contiguous 0
subtree distance 0
window 2125–2374
paths mf 0 paths fm 6
adjacent 1 contiguous 0
subtree distance 0
window 2150–2399
paths mf 0 paths fm 6
adjacent 1 contiguous 0
subtree distance 0
window 2325–2574
paths mf 0 paths fm 1
adjacent 1 contiguous 0
subtree distance 0
window 2350–2599
paths mf 0 paths fm 0
adjacent 0 contiguous 0
subtree distance 0
window 2375–2624
paths mf 0 paths fm 0
adjacent 0 contiguous 0
subtree distance 0
window 2400–2649
paths mf 0 paths fm 0
adjacent 0 contiguous 0
subtree distance 0
window 2425–2674
paths mf 0 paths fm 0
adjacent 0 contiguous 0
subtree distance 0

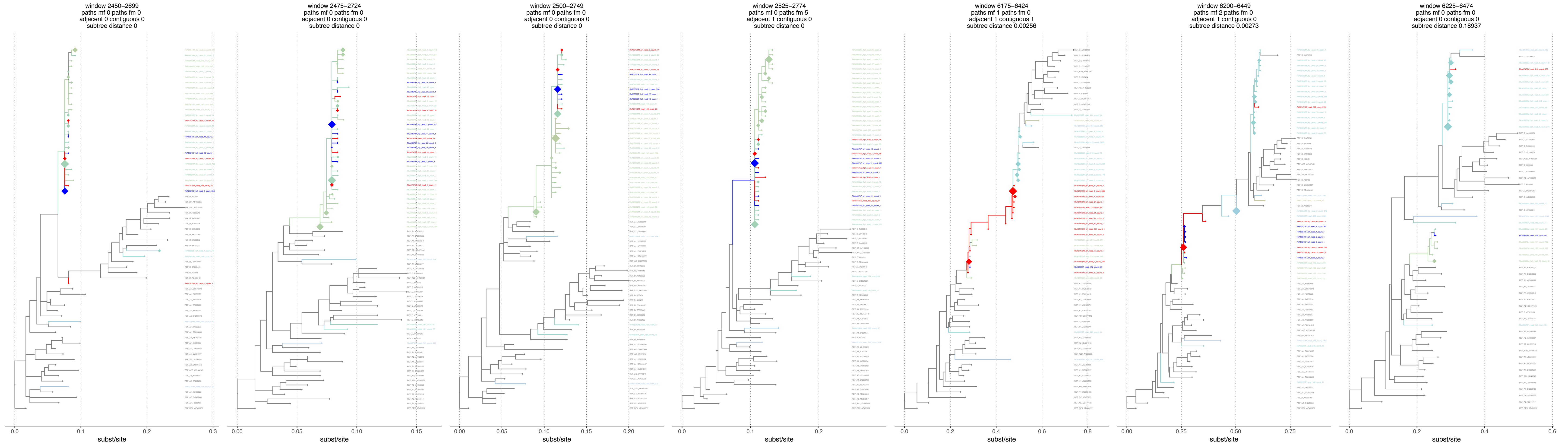
window 2450–2699
paths mf 0 paths fm 0
adjacent 0 contiguous 0
subtree distance 0
subst/site
window 2475–2724
paths mf 0 paths fm 0
adjacent 0 contiguous 0
subtree distance 0
subst/site
window 2500–2749
paths mf 0 paths fm 0
adjacent 0 contiguous 0
subtree distance 0
subst/site
window 2525–2774
paths mf 0 paths fm 5
adjacent 1 contiguous 0
subtree distance 0
subst/site
window 6175–6424
paths mf 1 paths fm 0
adjacent 1 contiguous 1
subtree distance 0.00256
subst/site
window 6200–6449
paths mf 2 paths fm 0
adjacent 1 contiguous 0
subtree distance 0.00273
subst/site
window 6225–6474
paths mf 0 paths fm 0
adjacent 0 contiguous 0
subtree distance 0.18937
subst/site

Deep sequence phylogenies
of male-female pair RkA00507M, RkA05021F (run 273)
for whom the phylogenetically inferred direction of transmission was inconsistent with clinical data.

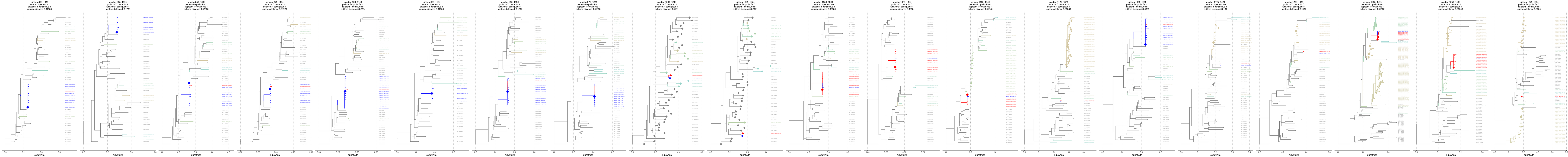
window 800–1049
paths mf 0 paths fm 1
adjacent 1 contiguous 1
subtree distance 0.01903
window 825–1074
paths mf 0 paths fm 1
adjacent 1 contiguous 1
subtree distance 0.01788
window 850–1099
paths mf 0 paths fm 1
adjacent 1 contiguous 1
subtree distance 0.02026
window 875–1124
paths mf 0 paths fm 1
adjacent 1 contiguous 1
subtree distance 0.01729
window 900–1149
paths mf 0 paths fm 1
adjacent 1 contiguous 1
subtree distance 0.02204
window 925–1174
paths mf 0 paths fm 1
adjacent 1 contiguous 1
subtree distance 0.01856
window 950–1199
paths mf 0 paths fm 1
adjacent 1 contiguous 1
subtree distance 0.01204
window 975–1224
paths mf 0 paths fm 1
adjacent 1 contiguous 1
subtree distance 0.01888
window 1000–1249
paths mf 0 paths fm 0
adjacent 1 contiguous 1
subtree distance 0.0181
window 1025–1274
paths mf 0 paths fm 0
adjacent 1 contiguous 1
subtree distance 0.02041
window 1050–1299
paths mf 1 paths fm 0
adjacent 1 contiguous 1
subtree distance 0.00889
window 1075–1324
paths mf 1 paths fm 0
adjacent 1 contiguous 1
subtree distance 0.0098
window 1100–1349
paths mf 1 paths fm 0
adjacent 1 contiguous 1
subtree distance 0.01346
window 1125–1374
paths mf 0 paths fm 0
adjacent 1 contiguous 1
subtree distance 0.00889
window 1150–1399
paths mf 0 paths fm 1
adjacent 1 contiguous 1
subtree distance 0.00805
window 1175–1424
paths mf 0 paths fm 0
adjacent 1 contiguous 1
subtree distance 0.0101
window 1200–1449
paths mf 0 paths fm 0
adjacent 1 contiguous 1
subtree distance 0.01937
window 1225–1474
paths mf 1 paths fm 0
adjacent 1 contiguous 1
subtree distance 0.01522
window 1250–1499
paths mf 1 paths fm 0
adjacent 1 contiguous 1
subtree distance 0.00549
window 1275–1524
paths mf 0 paths fm 0
adjacent 1 contiguous 1
subtree distance 0.0204

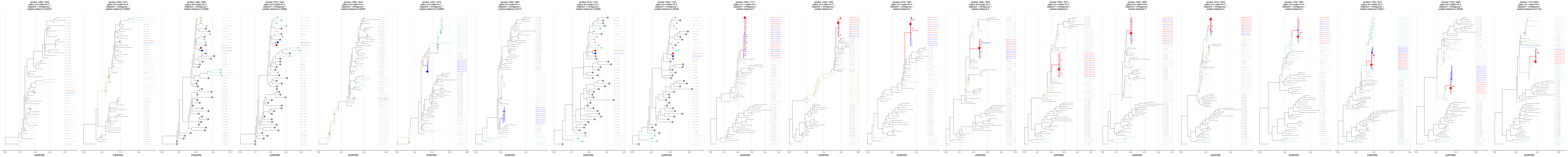

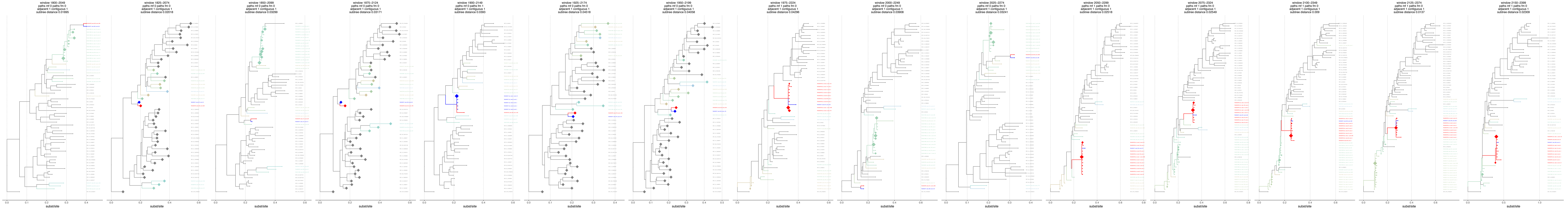
window 1800–2049
paths mf 0 paths fm 0
adjacent 1 contiguous 1
subtree distance 0.01895
window 1825–2074
paths mf 0 paths fm 0
adjacent 1 contiguous 1
subtree distance 0.02018
window 1850–2099
paths mf 0 paths fm 0
adjacent 1 contiguous 1
subtree distance 0.03299
window 1875–2124
paths mf 0 paths fm 0
adjacent 1 contiguous 1
subtree distance 0.03171
window 1900–2149
paths mf 0 paths fm 1
adjacent 1 contiguous 1
subtree distance 0.0393
window 1925–2174
paths mf 0 paths fm 0
adjacent 1 contiguous 1
subtree distance 0.04518
window 1950–2199
paths mf 0 paths fm 0
adjacent 1 contiguous 1
subtree distance 0.04058
window 1975–2224
paths mf 0 paths fm 0
adjacent 1 contiguous 1
subtree distance 0.04296
window 2000–2249
paths mf 0 paths fm 0
adjacent 1 contiguous 1
subtree distance 0.03908
window 2025–2274
paths mf 0 paths fm 0
adjacent 1 contiguous 1
subtree distance 0.03241
window 2050–2299
paths mf 1 paths fm 0
adjacent 1 contiguous 1
subtree distance 0.02518
window 2075–2324
paths mf 1 paths fm 0
adjacent 1 contiguous 1
subtree distance 0.02549
window 2100–2349
paths mf 1 paths fm 0
adjacent 1 contiguous 1
subtree distance 0.009
window 2125–2374
paths mf 1 paths fm 0
adjacent 1 contiguous 1
subtree distance 0.0137
window 2150–2399
paths mf 1 paths fm 0
adjacent 1 contiguous 1
subtree distance 0.02309
