# Supplementary material for: Inferring HIV-1 transmission networks and sources of epidemic spread in Africa with deep-sequence phylogenetic analysis
Source: Nat Commun. 2019 Mar 29;10:1411. doi: 10.1038/s41467-019-09139-4 (PMC6441045; doi:10.1038/s41467-019-09139-4)
Supplement: Supplementary file 5 — Reporting Summary [file 41467_2019_9139_MOESM5_ESM.pdf]

nature research

Corresponding author(s): Oliver Ratmann

Reporting Summary

Nature Research wishes to improve the reproducibility of the work that we publish. This form provides structure for consistency and transparency in reporting. For further information on Nature Research policies, see Authors & Referees and the Editorial Policy Checklist.

Statistical parameters

When statistical analyses are reported, confirm that the following items are present in the relevant location (e.g. figure legend, table legend, main text, or Methods section).

n/a	Confirmed	
☐	☒	The exact sample size (n) for each experimental group/condition, given as a discrete number and unit of measurement
☐	☒	An indication of whether measurements were taken from distinct samples or whether the same sample was measured repeatedly
☒	☐	The statistical test(s) used AND whether they are one- or two-sided<br>*Only common tests should be described solely by name; describe more complex techniques in the Methods section.*
☒	☐	A description of all covariates tested
☐	☒	A description of any assumptions or corrections, such as tests of normality and adjustment for multiple comparisons
☐	☒	A full description of the statistics including central tendency (e.g. means) or other basic estimates (e.g. regression coefficient) AND variation (e.g. standard deviation) or associated estimates of uncertainty (e.g. confidence intervals)
☒	☐	For null hypothesis testing, the test statistic (e.g. F, t, r) with confidence intervals, effect sizes, degrees of freedom and P value noted<br>*Give P values as exact values whenever suitable.*
☐	☒	For Bayesian analysis, information on the choice of priors and Markov chain Monte Carlo settings
☒	☐	For hierarchical and complex designs, identification of the appropriate level for tests and full reporting of outcomes
☐	☒	Estimates of effect sizes (e.g. Cohen's d, Pearson's r), indicating how they were calculated
☒	☐	Clearly defined error bars<br>*State explicitly what error bars represent (e.g. SD, SE, CI)*

Our web collection on statistics for biologists may be useful.

Software and code

Policy information about availability of computer code

Data collection	Assembly of HIV-1 reads:<br>- Deep sequencing reads were assembled with the SHIVER sequence assembly software, https://github.com/ChrisHIV/shiver; version 1.0.0.<br>- By default contigs were generated with IVA as part of SHIVER, http://sanger-pathogens.github.io/iva/, version XXX.<br>- Where no contigs could be generated with IVA as part of SHIVER: contigs were generated with SPAdes and metaSPAdes, http://cab.spbu.ru/software/spades/, version 3.10.
Data analysis	Read selection and deep sequence phylogenetic analysis of the population-based sample:<br>- Phyloscanner, https://github.com/BDI-pathogens/phyloscanner, version 1.1.2<br>- Phyloscanner.R.utilities, https://github.com/olli0601/Phyloscanner.R.utilities, version 0.7

For manuscripts utilizing custom algorithms or software that are central to the research but not yet described in published literature, software must be made available to editors/reviewers upon request. We strongly encourage code deposition in a community repository (e.g. GitHub). See the Nature Research guidelines for submitting code & software for further information.

Data

Policy information about availability of data

All manuscripts must include a data availability statement. This statement should provide the following information, where applicable:

- Accession codes, unique identifiers, or web links for publicly available datasets
- A list of figures that have associated raw data
- A description of any restrictions on data availability

The deep-sequence phylogenies and basic individual-level data analysed during the current study are available in the Dryad repository DOI: 10.5061/dryad.7h46hg2. HIV-1 reads are available on reasonable request to PANGEA-HIV. Additional individual-level data are available on reasonable request to RHSP.

The following data are available on Dryad:

Data set S1. Reconstructed deep-sequence phylogenies underlying inferred HIV-1 transmission networks in Rakai District, Uganda (compressed newick files).
Data set S2. Individual-level data (gender, sequencing date) (csv file).
Data set S3. Pairs with epidiomologic evidence into the direction of transmission, assuming they are linked (csv file).

Field-specific reporting

Please select the best fit for your research. If you are not sure, read the appropriate sections before making your selection.

☐ Life sciences ☐ Behavioural & social sciences ☒ Ecological, evolutionary & environmental sciences

For a reference copy of the document with all sections, see nature.com/authors/policies/ReportingSummary-flat.pdf

Ecological, evolutionary & environmental sciences study design

All studies must disclose on these points even when the disclosure is negative.

Study description	Deep-sequence phylogenetic analysis of HIV-1 virus from a population-based sample of 2,652 infected individuals in Rakai District, Uganda.
Research sample	The study was undertaken in a mostly rural district in south-central Uganda, bordering Tanzania to the south and Lake Victoria to the east. Individuals of the general population aged 14-49 years were cross-sectionally enrolled in 40 communities, as previously described (https://www.ncbi.nlm.nih.gov/pubmed/27470029). Virus from infected individuals was isolated, and deep-sequenced using standard protocols. The estimated sampling fraction was 47% among eligible, HIV-1 infected and infectious individuals. The key features of this study (general population, cross-sectional, high sampling fraction) were designed specifically to infer HIV-1 transmission networks of the general population, and to establish what epidemiologic inferences can be made from observed patterns in deep-sequence phylogenies.
Sampling strategy	Cross-sectional sample of the general population aged 14-49 years.
Data collection	Data collection included the following components: 1- community mobilization event; 2- census of all households in communities; 3- enrollment of eligible household members; 4- follow-up to enroll household members; 5- written informed consent; 6- interview; 7- collection of serum sample for HIV testing, future laboratory studies and viral sequencing; 8- participants offered results and counseling; 9- shipment of samples for sequencing to the UK; 10- RNA extraction; 11- deep-sequencing. Data collection has been in described in full detail here: https://www.ncbi.nlm.nih.gov/pubmed/27470029; https://www.ncbi.nlm.nih.gov/pmc/articles/PMC3502977/; https://www.ncbi.nlm.nih.gov/pmc/articles/PMC5597042/
Timing and spatial scale	Timing: from August 2011 to January 2015, encompassing two surveillance rounds of the Rakai Community Cohort Study. Spatial Scale: 40 communities of the Rakai Community Cohort Study. Please see further: https://www.ncbi.nlm.nih.gov/pubmed/27470029
Data exclusions	1,264 individuals self-reported using antiretrovirals at their first visit, and were not considered further as sequencing is challenging when virus is suppressed by treatment. Participant-reported use of ART in the cohort has been validated previously by the detection of antiretroviral drugs in plasma, showed high specificity (99% [96%-100%]) and high sensitivity (77% [70%-83%]), and suggests that most individuals who self-reported ART use were not infectious during the observation period (https://www.ncbi.nlm.nih.gov/pubmed/29194115).<br><br>Sequence samples from 1,226 (31.6%) of the remaining 3,878 individuals were not of sufficient quality for analysis and were excluded. Specifically, for phylogeny reconstruction, only paired-end merged reads of at least 250 base pairs (bp) in length were used, and subsequent deep-sequence inferences were performed on individuals whose reads covered the HIV-1 genome at a depth of at least 30 reads for 750bp or more. Thus, samples from 2,652 individuals were used for molecular epidemiological analyses, corresponding to an estimated 47.0% of eligible, infected and infectious individuals in RCCS communities.<br><br>Exclusions based on read length (250bp) were specified prior to phylogeny reconstruction to avoid significant data loss, see figure S1B. Exclusions based on minimum sequencing depth were varied in sensitivity analyses, see tables S4 to S6.
Reproducibility	Analysis is based on a population-based sample. Based on this sample, results described in sections "Direct transmission between two individuals cannot be proven from deep-sequence data alone", "No phylogenetic evidence of sub-epidemics amongst men

	having sex with men", "Direction of transmission can be frequently inferred in transmission networks reconstructed from deep-sequence data", "Inferred direction of transmission in source-recipient pairs has a low false-discovery rate" were reproducible and robust across a broad range of input specifications of the phyloscanner method. Please see tables S4 to S6.
Randomization	To validate phylogenetic inference on the direction of transmission, a validation panel of linked pairs with known direction of transmission within the population-based sample was used (n=36), and a further panel of linked pairs with suggested direction of transmission (n=35). No covariates of linked pairs were adjusted for, due to limited sample size.
Blinding	No blinding was performed to validate phylogenetic inference on the direction of transmission. Criteria for inferring the direction of transmission were pre-specified prior to analysis. Please see supplementary text S4 for full details.

Did the study involve field work? ☐ Yes ☒ No

Reporting for specific materials, systems and methods

Materials & experimental systems

n/a	Involved in the study
☐	☐ Unique biological materials
☐	☐ Antibodies
☐	☐ Eukaryotic cell lines
☐	☐ Palaeontology
☐	☐ Animals and other organisms
☐	☒ Human research participants

Methods

n/a	Involved in the study
☐	☐ ChIP-seq
☐	☐ Flow cytometry
☐	☐ MRI-based neuroimaging

Unique biological materials

Policy information about availability of materials

Obtaining unique materials	*Describe any restrictions on the availability of unique materials OR confirm that all unique materials used are readily available from the authors or from standard commercial sources (and specify these sources).*

Antibodies

Antibodies used	*Describe all antibodies used in the study; as applicable, provide supplier name, catalog number, clone name, and lot number.*
Validation	*Describe the validation of each primary antibody for the species and application, noting any validation statements on the manufacturer's website, relevant citations, antibody profiles in online databases, or data provided in the manuscript.*

Eukaryotic cell lines

Policy information about cell lines

Cell line source(s)	*State the source of each cell line used.*
Authentication	*Describe the authentication procedures for each cell line used OR declare that none of the cell lines used were authenticated.*
Mycoplasma contamination	*Confirm that all cell lines tested negative for mycoplasma contamination OR describe the results of the testing for mycoplasma contamination OR declare that the cell lines were not tested for mycoplasma contamination.*
Commonly misidentified lines (See ICLAC register)	*Name any commonly misidentified cell lines used in the study and provide a rationale for their use.*

Palaeontology

Specimen provenance	*Provide provenance information for specimens and describe permits that were obtained for the work (including the name of the issuing authority, the date of issue, and any identifying information).*
Specimen deposition	*Indicate where the specimens have been deposited to permit free access by other researchers.*

Dating methods

If new dates are provided, describe how they were obtained (e.g. collection, storage, sample pretreatment and measurement), where they were obtained (i.e. lab name), the calibration program and the protocol for quality assurance OR state that no new dates are provided.

☐ Tick this box to confirm that the raw and calibrated dates are available in the paper or in Supplementary Information.

Animals and other organisms

Policy information about studies involving animals; ARRIVE guidelines recommended for reporting animal research

Laboratory animals	*For laboratory animals, report species, strain, sex and age OR state that the study did not involve laboratory animals.*
Wild animals	*Provide details on animals observed in or captured in the field; report species, sex and age where possible. Describe how animals were caught and transported and what happened to captive animals after the study (if killed, explain why and describe method; if released, say where and when) OR state that the study did not involve wild animals.*
Field-collected samples	*For laboratory work with field-collected samples, describe all relevant parameters such as housing, maintenance, temperature, photoperiod and end-of-experiment protocol OR state that the study did not involve samples collected from the field.*

Human research participants

Policy information about studies involving human research participants

Population characteristics	Eligible individuals who provide written informed consent were administered a survey on their demographs, sexual behaviors and health-care seeking practices. Individuals were also asked to name their co-habitating sexual partners in order to identify couples, and to provide a serum sample for HIV-1 testing, future laboratory studies, and viral sequencing. Please see for further details https://www.nejm.org/doi/full/10.1056/NEJMoa1702150.
Recruitment	Briefly, the Rakai Community Cohort Study conducts a census in all communities to identify eligble indivduals two weeks before the survey. Eligible individuals include those able to give consent and between the ages of 15 and 49 years. Eligible individuals who provide written informed consent are administered a survey on their demographs, sexual behaviors and health-care seeking practices. Individuals are also asked to name their co-habitating sexual partners in order to identify couples, and to provide a serum sample for HIV-1 testing and future laboratory studies, including HIV-1 viral sequencing. Data for this particular study were collected between 2011 and 2015 from 40 agrarian, trading and fishing communities. Please see for further details https://www.nejm.org/doi/full/10.1056/NEJMoa1702150.

ChIP-seq

Data deposition

☐ Confirm that both raw and final processed data have been deposited in a public database such as GEO.

☐ Confirm that you have deposited or provided access to graph files (e.g. BED files) for the called peaks.

Data access links<br>*May remain private before publication.*	*For "Initial submission" or "Revised version" documents, provide reviewer access links. For your "Final submission" document, provide a link to the deposited data.*
Files in database submission	*Provide a list of all files available in the database submission.*
Genome browser session<br>(e.g. UCSC)	*Provide a link to an anonymized genome browser session for "Initial submission" and "Revised version" documents only, to enable peer review. Write "no longer applicable" for "Final submission" documents.*

Methodology

Replicates	*Describe the experimental replicates, specifying number, type and replicate agreement.*
Sequencing depth	*Describe the sequencing depth for each experiment, providing the total number of reads, uniquely mapped reads, length of reads and whether they were paired- or single-end.*
Antibodies	*Describe the antibodies used for the ChIP-seq experiments; as applicable, provide supplier name, catalog number, clone name, and lot number.*
Peak calling parameters	*Specify the command line program and parameters used for read mapping and peak calling, including the ChIP, control and index files used.*
Data quality	*Describe the methods used to ensure data quality in full detail, including how many peaks are at FDR 5% and above 5-fold enrichment.*
Software	*Describe the software used to collect and analyze the ChIP-seq data. For custom code that has been deposited into a community repository, provide accession details.*

Flow Cytometry

Plots

Confirm that:

- [ ] The axis labels state the marker and fluorochrome used (e.g. CD4-FITC).
- [ ] The axis scales are clearly visible. Include numbers along axes only for bottom left plot of group (a 'group' is an analysis of identical markers).
- [ ] All plots are contour plots with outliers or pseudocolor plots.
- [ ] A numerical value for number of cells or percentage (with statistics) is provided.

Methodology

Sample preparation	*Describe the sample preparation, detailing the biological source of the cells and any tissue processing steps used.*
Instrument	*Identify the instrument used for data collection, specifying make and model number.*
Software	*Describe the software used to collect and analyze the flow cytometry data. For custom code that has been deposited into a community repository, provide accession details.*
Cell population abundance	*Describe the abundance of the relevant cell populations within post-sort fractions, providing details on the purity of the samples and how it was determined.*
Gating strategy	*Describe the gating strategy used for all relevant experiments, specifying the preliminary FSC/SSC gates of the starting cell population, indicating where boundaries between "positive" and "negative" staining cell populations are defined.*

- [ ] Tick this box to confirm that a figure exemplifying the gating strategy is provided in the Supplementary Information.

Magnetic resonance imaging

Experimental design

Design type	*Indicate task or resting state; event-related or block design.*
Design specifications	*Specify the number of blocks, trials or experimental units per session and/or subject, and specify the length of each trial or block (if trials are blocked) and interval between trials.*
Behavioral performance measures	*State number and/or type of variables recorded (e.g. correct button press, response time) and what statistics were used to establish that the subjects were performing the task as expected (e.g. mean, range, and/or standard deviation across subjects).*

Acquisition

Imaging type(s)	*Specify: functional, structural, diffusion, perfusion.*
Field strength	*Specify in Tesla*
Sequence & imaging parameters	*Specify the pulse sequence type (gradient echo, spin echo, etc.), imaging type (EPI, spiral, etc.), field of view, matrix size, slice thickness, orientation and TE/TR/flip angle.*
Area of acquisition	*State whether a whole brain scan was used OR define the area of acquisition, describing how the region was determined.*

Diffusion MRI ☐ Used ☐ Not used

Preprocessing

Preprocessing software	*Provide detail on software version and revision number and on specific parameters (model/functions, brain extraction, segmentation, smoothing kernel size, etc.).*
Normalization	*If data were normalized/standardized, describe the approach(es): specify linear or non-linear and define image types used for transformation OR indicate that data were not normalized and explain rationale for lack of normalization.*
Normalization template	*Describe the template used for normalization/transformation, specifying subject space or group standardized space (e.g. original Talairach, MNI305, ICBM152) OR indicate that the data were not normalized.*
Noise and artifact removal	*Describe your procedure(s) for artifact and structured noise removal, specifying motion parameters, tissue signals and physiological signals (heart rate, respiration).*
Volume censoring	*Define your software and/or method and criteria for volume censoring, and state the extent of such censoring.*

Statistical modeling & inference

Model type and settings

Specify type (mass univariate, multivariate, RSA, predictive, etc.) and describe essential details of the model at the first and second levels (e.g. fixed, random or mixed effects; drift or auto-correlation).

Effect(s) tested

Define precise effect in terms of the task or stimulus conditions instead of psychological concepts and indicate whether ANOVA or factorial designs were used.

Specify type of analysis: ☐ Whole brain ☐ ROI-based ☐ Both

Statistic type for inference
(See Eklund et al. 2016)

Specify voxel-wise or cluster-wise and report all relevant parameters for cluster-wise methods.

Correction

Describe the type of correction and how it is obtained for multiple comparisons (e.g. FWE, FDR, permutation or Monte Carlo).

Models & analysis

n/a	Involved in the study
☐	☐ Functional and/or effective connectivity
☐	☐ Graph analysis
☐	☐ Multivariate modeling or predictive analysis

Functional and/or effective connectivity

Report the measures of dependence used and the model details (e.g. Pearson correlation, partial correlation, mutual information).

Graph analysis

Report the dependent variable and connectivity measure, specifying weighted graph or binarized graph, subject- or group-level, and the global and/or node summaries used (e.g. clustering coefficient, efficiency, etc.).

Multivariate modeling and predictive analysis

Specify independent variables, features extraction and dimension reduction, model, training and evaluation metrics.
